# Supplementary material for: Protocol for hypofractionated adaptive radiotherapy to the bladder within a multicentre phase II randomised trial: radiotherapy planning and delivery guidance
Source: BMJ Open. 2020 May 26;10(5):e037134. doi: 10.1136/bmjopen-2020-037134 (PMC7259864; doi:10.1136/bmjopen-2020-037134)
Supplement: Supplementary data [file bmjopen-2020-037134supp001.pdf]

HYBRID

A multicentre randomised phase II study of **HY**pofractionated **B**ladder **R**adiotherapy with or without **I**mage guided a**D**aptive planning

PROTOCOL

Version: 3.1

Dated: 13/08/2015

Chief Investigator:	Professor Robert Huddart
Sponsor:	The Institute of Cancer Research
Approval:	Cancer Research UK: Clinical Trials Awards & Advisory Committee (CTAAC)
Funders:	Cancer Research UK
Coordinating Trials Unit:	ICR Clinical Trials and Statistics Unit (ICR-CTSU)<br>The Institute of Cancer Research

Main REC Reference Number:	13/LO/1350
ICR-CTSU Protocol Number:	ICR-CTSU/2013/10039
ISRCTN:	ISRCTN18815596
ClinicalTrials.gov Identifier	NCT01810757
CRUK Reference Number:	CRUK/12/055

The HYBRID trial has been scientifically approved
by Cancer Research UK's Clinical Trials Awards & Advisory Committee (CTAAC)
The HYBRID trial is part of the National Institute for
Health Research Clinical Research Network Trial Portfolio

ICR The Institute of Cancer Research

CANCER RESEARCH UK

This protocol is a controlled document and should not be copied, distributed or reproduced without the written permission of the ICR-CTSU

HYBRID Protocol
ICR-CTSU

ADMINISTRATION

Scientific Coordination

Professor Robert Huddart (Chief Investigator)
Academic Radiotherapy/Division of Radiotherapy and Imaging
The Royal Marsden NHS Foundation Trust/The Institute Of Cancer Research
London
UK

Tel: 0208 661 3457
Robert.Huddart@icr.ac.uk

Shaista Hafeez (Clinical Coordinator)
The Royal Marsden NHS Foundation Trust/The Institute Of Cancer Research
London
UK

Tel: 0208 661 1143
Shaista.Hafeez@rmh.nhs.uk

Trial Coordination

ICR-CTSU (a UKCRC registered NCRI cancer clinical trials unit) is responsible for the day to day conduct of the trial.

ICR Clinical Trials & Statistics Unit (ICR-CTSU),
Division of Clinical Studies,
The Institute of Cancer Research,
Sir Richard Doll Building,
Cotswold Road,
Sutton, Surrey SM2 5NG

ICR-CTSU Scientific Lead : Emma Hall
Tel: 020 8722 4013
Emma.Hall@icr.ac.uk

ICR-CTSU Statistician: Clare Griffin/Helen Mossop
Tel: 020 8722 4062
HYBRID-icrctsu@icr.ac.uk

ICR-CTSU Senior Trial Manager: Rebecca Lewis
Tel: 020 8722 4081
HYBRID-icrctsu@icr.ac.uk

HYBRID Trial Manager: Joanna Illambas
Tel: 020 8722 4295
HYBRID-icrctsu@icr.ac.uk

Any questions relating to this protocol should be addressed in the first instance to the HYBRID Trial Manager within ICR-CTSU:

Email: HYBRID-icrctsu@icr.ac.uk
General enquiries: 020 8722 4238
Fax: 020 8770 7876

HYBRID Protocol
ICR-CTSU

PROTOCOL DEVELOPMENT GROUP

Professor Robert Huddart	Chief Investigator	The Institute of Cancer Research/Royal Marsden NHSFT
Dr Emma Hall	Deputy Director	ICR-CTSU, The Institute of Cancer Research, London
Dr Ann Henry	Consultant Oncologist	Leeds University Teaching Hospitals
Dr Vincent Khoo	Consultant Oncologist	Royal Marsden NHS Foundation Trust
Dr John Staffurth	Consultant Oncologist	Velindre NHS Trust
Dr Isabel Syndikus	Consultant Oncologist	Clatterbridge Centre for Oncology
Dr Vibeke Hansen	Physicist	Royal Marsden NHS Foundation Trust
Dr Helen McNair	Research Radiographer	Royal Marsden NHS Foundation Trust
Dr Shaista Hafeez	Research Fellow	Institute of Cancer Research/ Royal Marsden NHSFT
Clare Griffin	Statistician	Institute of Cancer Research
Rebecca Lewis	Senior Trials Manager	Institute of Cancer Research

The Trial Management Group (TMG) will be constituted from members of the Protocol Development Group and Principal Investigators from a subset of participating centres. A copy of the current membership of the TMG can be obtained from the HYBRID Trial Manager at ICR-CTSU.

Protocol Authorised by:

Name & Role	Signature	Date
Professor Robert Huddart<br>(Chief Investigator)		13/08/2015

This protocol describes the HYBRID trial and provides information about procedures for entering participants into this trial. The protocol should not be used as a guide for the treatment of patients outside of this trial.

Every care was taken in the preparation of this protocol, but corrections or amendments may be necessary. Protocol amendments will be circulated to participating sites as they occur, but sites entering patients for the first time are advised to contact ICR-CTSU to confirm they have the most recent version.

Hafeez S, *et al. BMJ Open* 2020; 10:e037134. doi: 10.1136/bmjopen-2020-037134

CONTENTS

1. INTRODUCTION ... 10
1.1. Background ... 10
1.1.1. Standard treatment for muscle invasive bladder cancer ... 10
1.1.2. Management of muscle invasive bladder cancer in patients unfit for standard treatment ... 10
1.1.3. Evidence for hypofractionated radiotherapy in bladder cancer ... 10
1.2. Challenges to ensuring accurate bladder radiotherapy delivery ... 11
1.3. Image guided radiotherapy (IGRT) ... 11
1.3.1. Evidence for adaptive-planned image guided radiotherapy (IGRT) ... 11
1.4. Known risks and benefits of adaptive hypofractionated radiotherapy ... 12
1.4.1. Potential benefits ... 12
1.4.2. Potential risks ... 12
1.5. Study rationale ... 13
2. TRIAL OBJECTIVES ... 13
2.1. Primary objective ... 13
2.2. Secondary objectives ... 13
2.3. Exploratory objectives ... 13
3. TRIAL DESIGN ... 13
4. STUDY ENDPOINTS ... 14
4.1. Primary endpoint ... 14
4.2. Secondary endpoints ... 14
4.3. Exploratory endpoints ... 14
5. PATIENT SELECTION & ELIGIBILITY ... 15
5.1. Number of participants ... 15
5.2. Source of participants ... 15
5.3. Inclusion criteria ... 15
5.4. Exclusion criteria ... 15
5.5. Lifestyle guidelines ... 15
6. SCREENING ... 15
6.1. Screening log ... 15
6.2. Procedure for obtaining informed consent ... 16
6.3. Participation in other research ... 16
7. RANDOMISATION ... 16
8. TRIAL ASSESSMENTS ... 17
8.1. Pre-randomisation assessments ... 17
8.2. Pre-treatment assessments ... 17
8.3. On-treatment assessments ... 17
8.4. Post radiotherapy assessments ... 17
8.4.1. 4 weeks from last radiotherapy fraction ... 17

8.4.2. 3 months from last radiotherapy fraction 17
8.4.3. 6 and 12 months (from last radiotherapy fraction) 17
8.4.4. 24 months (from last radiotherapy fraction) 18
8.4.5. Annually thereafter 18
8.5. Procedure at disease progression/recurrence 18
8.6. Withdrawal from treatment or follow-up 18
9. SCHEDULE OF ASSESSMENTS 19
10. TREATMENT 20
10.1. Standard pre-trial treatment 20
10.2. Treatment timelines 20
10.3. Radiotherapy planning and delivery 20
10.4. Treatment scheduling and gaps 20
10.5. Supportive care guidelines 20
10.6. Concomitant therapy 20
11. RADIOTHERAPY QUALITY ASSURANCE (QA) 21
12. SAFETY REPORTING 21
12.1. Adverse event (AE) 21
12.2. Serious adverse event (SAE) 21
12.3. Serious Adverse Reaction (SAR) 22
12.3.1. Definitions of causality 22
12.4. Related Unexpected SAE 22
12.5. Reporting Adverse Events to ICR-CTSU 22
12.6. Reporting Serious Adverse Events to ICR-CTSU 23
12.7. Serious Adverse Events exempt from expedited reporting 23
12.8. Review of Serious Adverse Events 23
12.9. Expedited Reporting of Related Unexpected SAEs 24
12.10. Follow up of Serious Adverse Events 24
12.11. Annual reporting of safety considerations 24
13. STATISTICAL CONSIDERATIONS 24
13.1. Statistical design and sample size justification 24
13.2. Treatment allocation 25
13.3. Primary endpoint definition 25
13.4. Secondary endpoint definitions 25
13.4.1. Local disease control at 3 months 25
13.4.2. Control rate of presenting symptoms 25
13.4.3. Patient reported outcomes (PRO) 25
13.4.4. Late toxicity 25
13.4.5. Time to local disease progression 25

13.4.6. Overall survival ... 26
13.4.7. Proportion of fractions benefiting from adaptive planning ... 26
13.4.8. Appropriate identification and correction of fractions requiring adaptive planning ... 26
13.5. Exploratory endpoints ... 26
13.6. Analysis plan ... 26
13.7. Interim analyses and stopping rules ... 27
14. TRIAL MANAGEMENT ... 27
14.1. Trial Management Group (TMG) ... 27
14.2. Trial Steering Committee (TSC) ... 27
14.3. Independent Data Monitoring Committee (IDMC) ... 27
15. RESEARCH GOVERNANCE ... 28
15.1. Sponsor responsibilities ... 28
16. TRIAL ADMINISTRATION & LOGISTICS ... 28
16.1. Site activation ... 28
16.2. Investigator training ... 28
16.3. Data acquisition ... 28
16.4. Central data monitoring ... 28
16.5. On-site monitoring ... 29
16.6. Completion of the study and definition of study end date ... 29
16.7. Archiving ... 29
17. PATIENT PROTECTION AND ETHICAL CONSIDERATIONS ... 29
17.1. Trial approvals ... 29
17.2. Trial conduct ... 29
17.3. Informed consent ... 29
17.4. Patient confidentiality ... 29
17.5. Data Protection Act (DPA) ... 30
17.6. Liability ... 30
18. FINANCIAL MATTERS ... 30
19. PUBLICATION POLICY ... 30
20. ASSOCIATED STUDIES ... 30
20.1. Patient reported outcome measures study ... 30
21. REFERENCES ... 31
A1. WHO performance status ... 33
A2. RTOG/EORTC late radiation morbidity scoring schema ... 33
A3. PATIENT REPORTED OUTCOMES STUDY ... 34
A3.1 Background ... 34
A3.2 Hypothesis ... 34
A3.3 Quality of life measures ... 34

A3.4 Study design 34
A3.5 Timing of data collection 35
A3.6 Compliance 35
A3.7 Statistical considerations 35
A4. GLOSSARY 36

Hafeez S, *et al. BMJ Open* 2020; 10:e037134. doi: 10.1136/bmjopen-2020-037134

HYBRID TRIAL SUMMARY

PROTOCOL TITLE	A multicentre randomised phase II study of **Hy**pofractionated **B**ladder **R**adiotherapy with or without **I**mage guided a**D**aptive planning
TARGET DISEASE	Muscle invasive bladder cancer
STUDY OBJECTIVES	The primary objective of HYBRID is to assess whether adaptive radiotherapy techniques when delivered at multiple centres can lead to a reduction in the level of acute non-genitourinary side effects experienced by patients with muscle invasive bladder cancer unsuitable for daily radical radiotherapy.
STUDY DESIGN	Multicentre phase II randomised controlled trial
TRIAL POPULATION	Patients with muscle invasive bladder cancer who are not suitable for cystectomy or daily radiotherapy
RECRUITMENT TARGET	62
TRIAL TREATMENT	Hypofractionated radiotherapy - all patients will be planned to receive six 6 Gray (Gy) fractions of radiotherapy delivered weekly (total dose: 36Gy) and will be randomised between either standard or adaptive planning.
PRIMARY ENDPOINT	Acute non-genitourinary grade 3 or greater toxicity (assessed using Common Terminology Criteria for Adverse Events (CTCAE) v.4)
SECONDARY ENDPOINTS	• Local disease control at 3 months<br>• Control rate of presenting symptoms (change from pre-radiotherapy CTCAE grades)<br>• Patient reported outcomes<br>• Late toxicity<br>• Time to local disease progression<br>• Overall survival<br>• Proportion of fractions benefiting from adaptive planning
EXPLORATORY ENDPOINTS	• Appropriate identification and correction of fractions requiring adaptive planning<br>• Dose Volume Histogram analysis of clinical target volume (CTV) coverage using anisotropic margins<br>• Concordance of clinician and patient reported toxicity measures
FOLLOW UP	Participants will be assessed for acute toxicity at each treatment visit and will complete a Patient Reported Outcomes (PRO) questionnaire at fraction 6. Participants will subsequently be assessed at the following intervals:<br>**4 weeks from end of radiotherapy:**<br>Assessment of acute toxicity (CTCAE v.4)<br>**3 months from end of radiotherapy:**<br>Assessment of acute toxicity (CTCAE v.4) and biopsy of tumour bed

under general anaesthetic (if not possible, flexible cystoscopy with visual inspection of tumour bed and urine cytology). PRO questionnaire.

6 & 12 months from end of radiotherapy:

Late toxicity assessment (CTCAE v.4 and RTOG) and flexible cystoscopy to visually assess local control (if not possible, pelvic CT scan and urine cytology). PRO questionnaire at 6 month visit only.

24 months from end of radiotherapy:

Late toxicity assessment (CTCAE v.4 and RTOG)

Assessment of disease control by clinical examination and flexible cystoscopy if possible.

Hafeez S, *et al. BMJ Open* 2020; 10:e037134. doi: 10.1136/bmjopen-2020-037134

TRIAL SCHEMA

62 patients with pT2-T4a N0 M0 bladder carcinoma unsuitable for standard daily radiotherapy

↓

Due to receive six 6Gy fractions of radiotherapy delivered weekly
(total dose: 36Gy over 6 weeks)

↓

RANDOMISE

1:1

Group 1:
Standard planning (control)

Planning CT scan post void. Clinical target volume (CTV): whole bladder plus any area of extravesical spread.

One 3D conformal plan will be generated with 1.5cm expansion margin.

Pre-treatment Cone Beam CT will be used to verify CTV coverage.

Group 2:
Adaptive planning (experimental)

Planning CT scan post void. CTV: whole bladder plus any area of extravesical spread.

Three 3D conformal plans will be generated:
1. Small; 2. Medium; 3. Large

Pre-treatment Cone Beam CT will be used to select appropriate plan, this will be confirmed by a second trained observer.

↓

Follow up

On treatment:
- Acute toxicity assessment at each fraction (Common Terminology Criteria for Adverse Events (CTCAE) v.4)
- Patient reported outcomes (modified Inflammatory Bowel Disease Questionnaire (IBDQ), King's Health Questionnaire (KHQ) and EQ5D) at fraction 6

4 weeks after last treatment:
- Acute toxicity assessment (CTCAE v.4)

3 months after last treatment:
- Acute toxicity assessment (CTCAE v.4)
- GA cystoscopy with tumour bed biopsy. (If not possible, flexible cystoscopy with visual inspection of tumour bed and urine cytology)
- Patient reported outcomes (IBDQ, KHQ, EQ5D)

6 and 12 months:
- Late toxicity assessment (CTCAE v.4 and RTOG)
- Flexible cystoscopy (or if not possible, urine cytology and CT scan of pelvis)
- Patient reported outcomes at 6 months only (IBDQ, KHQ, EQ5D)

24 months:
- Late toxicity assessment (CTCAE v.4 and RTOG)
- Assessment of disease control (clinical examination with flexible cystoscopy if possible)

1. INTRODUCTION

1.1. Background

1.1.1. Standard treatment for muscle invasive bladder cancer

Bladder cancer is the seventh most common UK cancer with 10,324 cases diagnosed in 2010 (1) . Muscle invasive bladder cancer accounts for 25% of new tumours and is associated with poor survival (<50% at 5 years (2)). For patients of good performance status, standard management would involve surgical excision of the bladder (radical cystectomy) or a course of radical radiotherapy given daily over 4-7 weeks (3).

Incidence of muscle invasive bladder cancer increases with age and many patients are not fit enough for major surgery, with its associated high treatment related mortality (4-6). National Institute for Health and Clinical Excellence (NICE) guidelines (3) recommend that: 'Radical radiotherapy is appropriate for patients who are not sufficiently fit for surgery or who wish to avoid cystectomy'. In the UK, radical radiotherapy for muscle invasive bladder cancer is delivered daily either to a total dose of 64 Gray (Gy) in 32 fractions (f) over 6.5 weeks or 55 Gy in 20f over 4 weeks.

Radiotherapy is an established treatment for muscle invasive bladder cancer, providing long term local control and allowing the patient to preserve their intrinsic bladder function (2, 7, 8). Technological advances such as three dimensional conformal planning now permit radiation dose to be shaped around the target, avoiding organs at risk (9). By employing reduced safety margins around the target volume, dose to organs at risk can be further limited. These highly precise methods rely on the target being in the same position each time radiotherapy is delivered.

1.1.2. Management of muscle invasive bladder cancer in patients unfit for standard treatment

Data collected as part of the SPARE trial (CRUK/07/011, ISRCTN61126465) suggest that around 70% of patients presenting with muscle invasive bladder cancer are unsuitable for standard radical therapy (surgery or daily radiotherapy) (10). This population presents a management dilemma, with an unmet clinical need. Despite relatively poor performance status many such patients would have normal life expectancy of several years but left untreated would experience (or be at risk of experiencing) significant disease related symptoms such as haematuria, urinary frequency, dysuria, pelvic pain, urinary incontinence and urinary obstruction.

There is relatively little published literature on radical treatment options for patients unfit for standard daily treatment. In current practice, patients are normally treated with hypofractionated radiotherapy where fewer but larger fractions are given at each visit. Despite this palliative intent, a proportion of patients survive for two or more years (11), thus treating with a higher biological effective dose with the aim of achieving local control could be expected to improve both survival and quality of life.

1.1.3. Evidence for hypofractionated radiotherapy in bladder cancer

The only multicentre randomised study of hypofractionated radiotherapy in muscle invasive bladder cancer (MRCBA09) investigated palliative regimens with low effective biological doses, randomising 500 patients between 35Gy in 10f over 2 weeks or 21Gy in 3f over 1 week (11). The trial included patients of poorer prognosis than those who will be included in HYBRID; despite this both treatment groups achieved symptom control or improvement in over 65% of participants and local control was achieved in 18/33 (55%) and 14/37 (38%) participants in the 35Gy and 21Gy groups respectively.

Other groups have reported single centre retrospective series of hypofractionated weekly radiotherapy, most often using 6Gy per fraction to a dose of 30-36 Gy over 5-6 weeks(12-14). This fractionation schedule delivers a higher biological dose to the tumour than the BA09 schedules with late toxicity theoretically similar to that seen for a standard 2Gy/f schedule (11). The 2Gy equivalent dose using an α/β ratio of 10 for 35Gy/10f, 21Gy/3f and 36Gy/6f are 39.75, 29.75 and 48Gy respectively. Retrospective reports suggest that the 36Gy/6f schedule is well tolerated, with acute toxicity less than that of standard fractionated treatment and acceptable (but variable) late toxicity. The best estimate taken from across these studies

might be Radiation Therapy Oncology Group (RTOG) grade 3-4 late toxicity rate of 5-10%. However, because of the retrospective collection of many of these data, reports may be subject to significant bias and potential underreporting of adverse outcomes, so prospective verification is important to confirm toxicity experienced by patients. Three month local control rates of >60% have been reported and again would benefit from prospective verification. Survival is similar to that which would be expected in this population, but all studies had a proportion of long term survivors. An attraction of a schedule where treatment is delivered weekly is that close monitoring of acute toxicity can be undertaken and if necessary radiotherapy can be modified to fit the patient's tolerance of the treatment. Prospective multicentre assessment of the 36Gy in 6 fractions weekly schedule has not been performed.

1.2. Challenges to ensuring accurate bladder radiotherapy delivery

A course of standard radiotherapy is planned using a CT scan taken when the patient has an empty bladder. Currently, each fraction of radiotherapy is delivered based on the target position on a snapshot CT image taken at the time of planning and knowledge of the bladder position with each treatment fraction is unknown. It is assumed that this initial scan is representative of bladder position throughout the course of treatment and one plan is generated by the radiotherapy physics department for use throughout treatment. Safety margins of 1.5-2cm are added to generate the planning target volume (PTV) to account for uncertainty introduced by microscopic disease not visible on the CT scan, errors in patient set up and day-to-day variation in bladder filling.

The bladder is a mobile, deformable structure however and bladder volume can vary markedly during a course of radiotherapy, despite delivering treatment to a perceived empty bladder (15-21). Movement of the bladder wall by more than 1.5cm has been documented in up to 60% of patients, resulting in inadequate coverage by radiotherapy fields in 33% (19). A study at the Royal Marsden Hospital (RMH) (22) reported that up to 57% of treatment may be delivered with some element of geographic miss (where the radiotherapy does not "hit" the tumour volume), despite employing safety margins of 1.5cm around the empty bladder (23). Geographical miss leads to the possibility of reduced tumour control, but larger margins would increase the treated volume and the amount of normal tissue exposed to high dose radiation, potentially leading to increased toxicity. Set-up uncertainties may be reduced through ensuring consistency of patient position, but bladder volume variation and displacement may still exist. An alternative method of assessing this and correcting for it is required.

1.3. Image guided radiotherapy (IGRT)

Recent advances enable images of soft tissue to be obtained within the radiotherapy treatment room using cone beam CT (CBCT) and other technologies. Although of lower resolution than the original planning CT scan, these can be used both to match bony anatomy automatically and to visualise bladder position, thus helping to ensure that the PTV is correctly delivered. The bladder is predicted to be one of the key tumour sites to achieve major benefits from IGRT techniques (13, 19) and in 2012 the National Radiotherapy Implementation Group recommended that volumetric imaging should be used for treatment verification for every patient receiving hypofractionated radiotherapy to the bladder (24).

1.3.1. Evidence for adaptive-planned image guided radiotherapy (IGRT)

Studies of CBCT have ascertained the extent of geographic miss in bladder cancer patients (16, 18, 21, 23). In an imaging study in the patient population included in HYBRID, 42/83 (51%) fractions had some displacement of the cone beam target volume (PTVcb) outside of the planned PTV. An average of 8% (and up to a maximum of 35%) of the bladder PTV was missed (23). This study included retrospective treatment planning which showed that an adaptive radiotherapy approach would have achieved complete coverage in 73% of treatment fractions, with substantial improvements seen in the remainder. Geographic misses have a particularly high impact on hypofractionated schedules as one fraction of treatment delivered off target represents a significant proportion (15-20%) of the total dose. However increased margins would cause exposure of more normal tissue to large doses per fraction and could lead to increased toxicity, particularly in this less fit patient group. The challenge is to use this imaging technology and to implement IGRT for patient benefit. Two potential methodologies have been suggested:

- Composite plan - offline assessments are used to produce a composite plan covering the range of possible bladder positions which are then covered by a smaller margin. This technique is not applicable in the context of hypofractionated radiotherapy as too few fractions are delivered to produce the composite plan.
- 'Plan of the day' methodology - at the planning stage, a series of treatment plans, rather than one, are created to cover a range of bladder filling and positional variability. A CBCT taken prior to treatment can be compared against the original planning scan to ascertain the possibility of any geographic miss after set up correction. If variation in bladder volume is demonstrated, an alternative plan can be selected to ensure the whole target is treated. This methodology reduces geographic misses, often allows selection of plans with smaller margins and is deliverable by radiography staff (23).

The 'plan of the day' concept has been evaluated in a non-randomised single centre phase II feasibility study at the RMH (Adaptive predictive planning for hypofractionated bladder radiotherapy: APPLY; ISRCTN80815524). Twenty-five patients unfit for radical daily radiotherapy were recruited to this study of hypofractionated adaptive radiotherapy (as radical or palliative treatment). Three treatment plans were generated for each participant; small, medium (standard) and large. 24 out of 25 patients completed their planned treatments and adaptive plans were selected for delivery of 55% of fractions (49% small, 6% large). Compared to standard planning this resulted in a 45% average reduction in PTV. Concordance between online and blinded offline plan selection was 91% (126/139), and there was 99% coverage of the CTV by the 95% isodose curve (25). With a median follow-up of 8.4 months in the 20 patients with localised disease at presentation, 9 (45%) patients were well with no sign of bladder carcinoma recurrence and 11 (55%) had died, 6 from bladder carcinoma and 5 from other causes. 5/20 (25%), 1/20 (5%) and 2/20 (10%) patients experienced CTCAE grade 3 acute genitourinary, gastrointestinal and other toxicities, respectively. 14 patients were assessable 6 months or more post-radiotherapy and of these, 4 and 1 patients experienced late grade 2 genitourinary and gastrointestinal toxicity, respectively. There were no grade 3 or higher late toxicities reported (26).

Adaptive planning does not reduce the volume of bladder irradiated but it is expected to reduce the volume of non-bladder tissue irradiated. As such, it is the non-genitourinary toxicity that is of primary interest with the expectation that there will be less toxicity associated with adaptive planning. Grade 3 and above is considered to be the toxicity level of interest as toxicities below this grade can be controlled with minimal intervention and have little impact on activities of daily living. Grade 3 toxicities are severe or medically significant but not immediately life-threatening, cause hospitalisation and can be disabling, limiting the self care capabilities of patients.

1.4. Known risks and benefits of adaptive hypofractionated radiotherapy

1.4.1. Potential benefits

It is anticipated that the use of adaptive radiotherapy techniques will improve the accuracy of treatment. This should lead to benefits for patients in terms of both reduced exposure of normal tissue to high doses of radiotherapy and an associated reduction in non-genitourinary side effects and also a reduced risk of geographic misses.

1.4.2. Potential risks

Hypofractionated radiotherapy uses larger doses per fraction and this may alter the side effect profile compared to standard radiotherapy. Toxicity data will be reviewed by the Independent Data Monitoring Committee throughout the trial and any concerns will be raised with the Trial Management Group as appropriate. Adaptive radiotherapy is more complex due to the need to select the most appropriate plan and thus extends treatment delivery time – treatment delivery time will be collected as part of the HYBRID dataset.

In addition, the use of CBCT leads to radiation exposure which is additional to that of standard radiotherapy delivered without the use of IGRT techniques. The risks of any resulting carcinogenic effect from CBCT are

Hafeez S, *et al. BMJ Open* 2020; 10:e037134. doi: 10.1136/bmjopen-2020-037134

considered minimal as the exposure represents <1% of the therapeutic radiation dose and the life expectancy of this group of patients is likely to be less than 10 years.

1.5. Study rationale

Patients with muscle invasive bladder cancer who are unable to receive daily radiotherapy often experience genitourinary symptoms which interfere with their daily life due to the burden of disease within their bladder. Although unsuitable for daily radiotherapy, these patients may otherwise have a life expectancy of several years. Treatment with hypofractionated radiotherapy at a dose of 36 Gy in 6 fractions would provide these patients the opportunity to attain local control of their tumour, with an associated reduction in symptoms. When hypofractionation is used, the precise delivery of each treatment is arguably even more important than in standard fractionation schedules, both to ensure maximal tumour control and minimise toxicity, as each treatment represents 15-20% of the total dose.

Improving radiotherapy quality is of clear importance in bladder cancer treatment. It is now important to assess whether the pilot work relating to adaptive radiotherapy conducted as part of single centre studies can be successfully translated into radiotherapy practice across the UK and to prospectively assess the benefits for patients as part of a multicentre randomised trial.

HYBRID is a multicentre study of the adaptive radiotherapy methodology in patients receiving weekly bladder radiotherapy and will provide the opportunity to standardise treatment for this patient group, allow the collection of prospective multicentre data on the 36 Gy in 6 fractions regimen, test the feasibility of delivering adaptive methodology in a number of NHS sites and assess whether this methodology provides patients with the opportunity to reduce radiotherapy related side effects.

2. TRIAL OBJECTIVES

2.1. Primary objective

The primary objective of HYBRID is to assess whether adaptive radiotherapy techniques when delivered at multiple centres can lead to a reduction in the level of acute non-genitourinary toxicity experienced by patients with muscle invasive bladder cancer unsuitable for daily radical radiotherapy.

2.2. Secondary objectives

Secondary objectives of HYBRID are to establish the local disease control rates of hypofractionated bladder radiotherapy as measured at 3 months, and assess time to local disease progression and the overall survival time of patients who have received hypofractionated radiotherapy. HYBRID will also investigate the control rate of presenting symptoms, the effect of hypofractionated treatment on late radiotherapy side effects and assess patient reported outcomes. The proportion of fractions benefiting from adaptive planning will also be assessed.

2.3. Exploratory objectives

HYBRID will measure the appropriate identification and correction of fractions requiring adaptive planning, the dose volume histogram analysis of CTV coverage using anisotropic margins and will explore the concordance of clinician and patient reported toxicity measures.

3. TRIAL DESIGN

HYBRID is a multicentre randomised controlled phase II trial in patients with localised muscle invasive bladder cancer who are unsuitable for daily radical radiotherapy treatment.

All patients will be planned to receive six 6Gy fractions of image guided radiotherapy delivered weekly (total dose: 36Gy) and will be randomised to standard or adaptive planning.

Participants allocated to the standard planning group will have one radiotherapy plan generated and this will be used to deliver all 6 treatments, with a cone beam CT scan prior to treatment delivery which can be used by the local investigator to adjust treatment delivery according to local practice.

Participants allocated to adaptive planning will have three radiotherapy plans generated; small, medium and large. A cone beam CT taken prior to each treatment delivery will be used to select the most appropriate plan of the day.

Acute toxicity data will be collected throughout treatment and at 4 weeks and 3 months from the end of radiotherapy. Local control will be assessed by cystoscopy at 3, 6 and 12 months. Late toxicity and survival data will be collected at 6, 12 and 24 months, after this time only basic routine follow-up data will be collected. Participants will be asked to complete a patient reported outcomes questionnaire prior to treatment, at the end of treatment and at 3 and 6 months.

4. STUDY ENDPOINTS

4.1. Primary endpoint

- Acute non-genitourinary grade 3 or greater toxicity (CTCAE v.4) occurring during radiotherapy and up to 3 months following treatment completion

4.2. Secondary endpoints

- Local disease control rate at 3 months
- Control rate of presenting symptoms – change in CTCAE grades from pre-radiotherapy to 3 months following treatment completion
- Patient reported outcomes
- Late toxicity
- Time to local disease progression
- Overall survival
- Proportion of fractions benefiting from adaptive planning
- Appropriate identification and correction of fractions requiring adaptive planning by retrospective independent central review of scan and treatment data

4.3. Exploratory endpoints

- Dose Volume Histogram analysis of CTV coverage using anisotropic margins
- Concordance of clinician and patient reported toxicity measures at each time point where both were assessed

5. PATIENT SELECTION & ELIGIBILITY

5.1. Number of participants

The aim is to recruit 62 participants, 31 to each treatment allocation.

5.2. Source of participants

Participants will be recruited from approximately 10 participating sites in the UK. Patients will be approached about participation in HYBRID if they are considered at multi-disciplinary team meetings to be unfit for daily radical radiotherapy and fulfil the eligibility criteria.

5.3. Inclusion criteria

1. Written informed consent
2. Age ≥18 years
3. Histologically or cytologically confirmed bladder carcinoma
4. Bladder cancer staged T2-T4a N0 M0
5. Unsuitable for radical cystectomy or daily fractionated radiotherapy for any reason (including performance status, co-morbidity, patient refusal)
6. Expected survival >6 months
7. WHO performance status 0-3 (Appendix 1)
8. Willing to undergo post treatment cystoscopy
9. Able to attend for post treatment follow up

5.4. Exclusion criteria

1. Nodal or metastatic disease
2. Concurrent malignancy within 2 years of randomisation (not including non melanomatous skin carcinoma, previous non muscle invasive bladder tumours, NCCN low risk prostate cancer (T1/T2a, Gleason 6 PSA <10), in situ carcinoma of any site)
3. Previous pelvic radiotherapy
4. Urinary catheter in-situ
5. Any other contra-indication to radiotherapy (e.g. inflammatory bowel disease)

5.5. Lifestyle guidelines

It is highly unlikely that the patient population included in HYBRID will be at risk of pregnancy or fathering a child. However if this is a possibility for any individual patient, this should be discussed and the patient should be advised to use barrier protection and avoid conception for 12 months after treatment.

6. SCREENING

6.1. Screening log

All participating centres will be required to keep a detailed log of all patients with muscle invasive bladder cancer discussed at multi-disciplinary team meetings who are considered unsuitable for cystectomy or daily radiotherapy treatment. This log will capture the following information:

- Date patient identified
- Number of patients approached/accepting/declining participation/ineligible
- Screening outcome

- Trial ID (if applicable)
- Reasons for ineligibility / not approaching / declining as applicable

This information will be used to monitor recruitment activity. No patient identifiable data will be collected at this stage.

6.2. Procedure for obtaining informed consent

The Principal Investigator (or designated individual) must ensure that each trial patient is fully informed about the nature and objectives of the trial and possible risks associated with participation. Participants should be given the current REC approved HYBRID patient information sheet for their consideration. Patients should only be asked to consent to the study after they have had sufficient time to consider the trial, and the opportunity to ask any further questions.

No protocol required assessments should be conducted until the HYBRID consent form has been signed and dated by both the patient and the Investigator, unless they are performed routinely as part of standard patient care.

Patients who consent to HYBRID will be asked to consent to participate in the Patient Reported Outcomes (PRO) sub-study. Patients should be made aware that participation in the PRO sub-study is entirely voluntary. Refusal to participate in the PRO sub-study will not result in ineligibility to participate in the main clinical trial and will not impact the medical care received.

Confirmation of the patient's consent and the informed consent process must be documented in the patient's medical notes. A copy of the signed consent form should be provided to the patient and the original retained in the investigator site file, which must be available for verification by ICR-CTSU study staff.

6.3. Participation in other research

Patients who fulfil the eligibility criteria will be given the opportunity to participate in HYBRID even if they have participated in other research prior to recruitment.

HYBRID participants will not be permitted to participate in any trials whilst they are being treated within HYBRID or for 3 months afterwards.

Participation in other research will be considered on a case by case basis by the Trial Management Group.

7. RANDOMISATION

Patients must be randomised centrally by the trials unit (ICR-CTSU) before trial treatment can commence.

Patients should be randomised by telephoning ICR-CTSU on:

020 8643 7150

09.00-17.00 (UK time) Monday to Friday

Randomisation should take place ideally within 4 and within a maximum of 6 weeks prior to the planned start date of radiotherapy. An eligibility and randomisation checklist must be completed prior to randomisation.

The following information will be required at randomisation:

- Name of hospital, consultant and person randomising patient
- Confirmation that patient has given written informed consent for trial and for any sub-studies;
- Confirmation that patient is eligible for the trial by completion of the eligibility checklist
- Patient's full name, hospital number, date of birth, postcode and NHS/CHI number

The caller will be given the patient's unique randomisation number (Trial ID) and treatment allocation (see section 14.2).

ICR-CTSU will send written confirmation of trial entry to the data management contact at the recruiting centre.

8. TRIAL ASSESSMENTS

8.1. Pre-randomisation assessments

The following assessments should be conducted prior to randomisation:

- Histological confirmation of bladder cancer
- Radiological assessment of muscle invasive bladder cancer. Ideally within 4 weeks and within a maximum of 6 weeks prior to randomisation. To include a minimum of CT of abdomen and pelvis with chest x-ray (CT of chest, abdomen and pelvis; or MRI pelvis and CT chest and abdomen are also acceptable, according to local practice).

8.2. Pre-treatment assessments

The following assessments should be conducted within 14 days prior to the start of treatment:

- Assessment of baseline symptoms (CTCAE v. 4)
- Full blood count, urea and electrolytes
- Patient reported outcomes (IBDQ, KHQ and EQ5D)

8.3. On-treatment assessments

The following assessments should be conducted weekly (ideally prior to delivery of radiotherapy)

- Acute toxicity assessment (CTCAE v.4)

  At fractions 2, 4 and 6

- Full blood count, urea and electrolytes

  At fraction 6:

- Patient reported outcomes (IBDQ, KHQ and EQ5D)

8.4. Post radiotherapy assessments

8.4.1. 4 weeks from last radiotherapy fraction

- Acute toxicity assessment (CTCAE v.4)

8.4.2. 3 months from last radiotherapy fraction

- Acute toxicity assessment (CTCAE v.4)
- Cystoscopy under general anaesthetic, with tumour bed biopsy (if not possible, flexible cystoscopy with visual inspection of tumour bed and urine cytology. If flexible cystoscopy is not possible please contact ICR-CTSU for advice).
- Patient reported outcomes (IBDQ, KHQ and EQ5D)

8.4.3. 6 and 12 months (from last radiotherapy fraction)

- Late toxicity assessment (CTCAE v.4 and RTOG (Appendix 2))
- Flexible cystoscopy with visual inspection of tumour bed (if not possible, urine cytology and CT scan of pelvis)
- Patient reported outcomes at 6 months only (IBDQ, KHQ and EQ5D)

8.4.4. 24 months (from last radiotherapy fraction)

- Late toxicity assessment (CTCAE v.4 and RTOG)
- Assessment of disease status, by clinical examination and flexible cystoscopy if possible.

8.4.5. Annually thereafter

Patients will not be required to undergo any trial specific investigations; however data will be requested annually from standard follow up visits relating to:

- Assessment of disease status
- Survival

8.5. Procedure at disease progression/recurrence

Participants should be treated according to local clinical judgement at disease progression/recurrence.

8.6. Withdrawal from treatment or follow-up

Participants may withdraw from trial treatment at any time at their own request, or they may be withdrawn at the discretion of the Principal Investigator. Reasons for withdrawal may include:

- Disease progression
- Unacceptable toxicity
- Co-morbidities

Participants who discontinue treatment should continue to be followed up. If a patient withdraws from further follow-up a trial deviation form should be submitted to ICR-CTSU stating whether the patient has withdrawn consent for information to be sent to the ICR-CTSU or whether they simply no longer wish to attend trial follow up visits. In the very rare event that a patient requests that their data is removed from the study entirely, the implications of this should be discussed with the patient first to ensure that this is their intent and, if confirmed, ICR-CTSU should be notified in writing. If this request is received after results have been published the course of action will be agreed between the Sponsor and Independent Data Monitoring and Trial Steering Committees.

Hafeez S, *et al. BMJ Open* 2020; 10:e037134. doi: 10.1136/bmjopen-2020-037134

9. SCHEDULE OF ASSESSMENTS

Visit/Assessment	Pre-randomisation	Up to 14 days pre-treatment	On treatment (before each fraction)	4 weeks after completion of radiotherapy	3 months after completion of radiotherapy	6 months after completion of radiotherapy	12 months after completion of radiotherapy	24 months after completion of radiotherapy	Annually thereafter
Histological confirmation of bladder cancer	X								
Radiological assessment of bladder cancer (minimum CT abdomen and pelvis and chest x-ray)	X[1]								
Acute toxicity assessment (CTCAE v.4)		X	X	X	X				
Full blood count, urea and electrolytes		X	X[2]						
Patient reported outcomes questionnaire (IBDQ, KHQ and EQ5D)		X	X[3]		X	X			
Cystoscopy under general anaesthetic with tumour bed biopsy *(if not possible, flexible cystoscopy with visual inspection of tumour bed and urine cytology)*					X				
Late toxicity assessment (CTCAE v.4 and RTOG)						X	X	X	
Flexible cystoscopy with visual inspection of tumour bed *(if not possible, urine cytology and pelvic CT scan)*						X	X		
Assessment of disease status								X	X

Footnotes

1. Baseline radiological assessment should take place ideally within 4 weeks and within a maximum of 6 weeks prior to randomisation
2. Full blood count, urea and electrolytes prior to fractions 2, 4 and 6 only
3. PRO questionnaire at fraction 6 only

10. TREATMENT

Details of radiotherapy planning are given in the accompanying HYBRID radiotherapy planning and delivery guidelines, supplied by ICR-CTSU, which must be used as the primary source for radiotherapy planning within HYBRID.

10.1. Standard pre-trial treatment

All participants should have a transurethral resection of bladder tumour (if possible) or tumour biopsy prior to trial entry.

10.2. Treatment timelines

Radiotherapy should commence within 6 weeks following randomisation (to allow sufficient time for planning).

10.3. Radiotherapy planning and delivery

All participants will be planned to receive six 6Gy fractions of radiotherapy delivered weekly (total dose: 36Gy). Participants will be allocated at random to either standard or adaptive planning. Radiotherapy planning and outlining should be carried out in accordance with the guidelines in the current version of the radiotherapy planning and delivery guidelines, available in the HYBRID site investigator file and on request from ICR-CTSU (HYBRID-icrctsu@icr.ac.uk).

10.4. Treatment scheduling and gaps

Treatment can start on any day of the week and each fraction should be given on the same day of the week at weekly intervals +/- 2 day(s).

A gap of up to 9 (standard 7 days plus 2) days is acceptable in the event of machine service or breakdown. If the treatment machine is unavailable for more than 2 days, please contact the HYBRID trial manager.

10.5. Supportive care guidelines

In the event of patient catheterisation during the course of treatment it is expected that the participant will continue and complete radiotherapy in accordance with their allocated treatment group. As the bladder requires emptying prior to treatment delivery, the catheter must be on free flow in circumstances where there is a leg bag or voided in circumstances where there is a flip-valve.

Participants’ symptoms should be managed according to local practice, although the following are suggestions for patient care:

Anaemia: Patients should be maintained by transfusion with haemoglobin above 11 grams. Iron deficiency should be treated with iron supplementation.

Dysuria/Frequency: Check for evidence of infection and treat if present with appropriate antibiotics, anticholinergics (eg oxybutynin, tolterodine), NSAIDs, analgesics.

Diarrhoea: Loperamide or opioid

Proctitis: steroid suppository +/- local anaesthetics (e.g. sheriproct, proctosedyl)

10.6. Concomitant therapy

All medication considered necessary for the patients’ welfare and which is not expected to interfere with the evaluation of the treatment may be given at the discretion of the investigator.

Concomitant chemotherapy and/or Carbogen Nicotinamide are not considered standard of care for this patient population but may be used cautiously at investigators’ discretion, however ICR-CTSU should be contacted in advance to gain approval from the Trial Management Group.

11. RADIOTHERAPY QUALITY ASSURANCE (QA)

A comprehensive QA programme for the HYBRID trial will be designed and implemented by the NCRI Radiotherapy Clinical Trials Quality Assurance (NCRI RTTQA) group. This will include pre-trial and on-trial components. Selection of appropriate treatment plans for the adaptive planning group will be independently monitored as part of the ongoing radiotherapy QA process. A member of the NCRI RTTQA group will visit all participating centres during the treatment delivery to the first patient receiving adaptive radiotherapy at each site. Subsequent plan selection will be subject to retrospective blind review by members of the NCRI RTTQA group, and the outcome of this will be fedback to centres in order to maintain and reinforce standard selection parameters across all participating sites.

11.1. Pre-trial quality assurance programme

The following will need to be completed by participating centres prior to site activation.

1. Facility questionnaire
2. Process document
3. Benchmark outlining case
4. Benchmark planning case
5. IGRT competency programme - on-line training package, practical workshop and independent competency check

11.2. On-trial quality assurance programme

1. On site independent plan selection review. This will be completed for the first adaptive radiotherapy participant at each investigator site by the NCRI RTTQA group prior to the site recruiting a subsequent participant.
2. Remote retrospective plan selection review for all adaptive radiotherapy patients
3. Plan and image data collection

12. SAFETY REPORTING

12.1. Adverse event (AE)

Any untoward medical occurrence in a patient or clinical trial subject administered a research procedure; events do not necessarily have a causal relationship with the procedure.

12.2. Serious adverse event (SAE)

An SAE is any untoward medical occurrence that occurs after the commencement of study treatment and:

- results in death;
- is life-threatening;
- requires hospitalisation or prolongation of existing hospitalisation;
- results in persistent or significant disability or incapacity;
- consists of a congenital anomaly or birth defect;
- is otherwise considered medically significant by the investigator; or
- is any non-genitourinary adverse event grade 3 or higher (CTCAE v4) occurring up to 3 months after completion of radiotherapy

Important adverse events that are not immediately life-threatening or do not result in death or hospitalisation, but may jeopardise the subject or require intervention to prevent one of the other outcomes listed in the definition above, may also be considered serious.

Progression of the indicated disease and death due to progression of the indicated disease are not considered SAEs.

Pregnancy or aid in the conception of a child whilst participating in a trial is not itself considered an SAE but should be followed up for congenital anomalies.

12.3. Serious Adverse Reaction (SAR)

A serious adverse reaction is an SAE that is suspected as having a causal relationship to the research procedure, as assessed by the investigator responsible for the care of the patient. A suspected causal relationship is defined as possibly, probably or definitely related (see definitions of causality table).

12.3.1. Definitions of causality

Relationship	Description
Unrelated	There is no evidence of any causal relationship with the trial procedure
Unlikely	There is little evidence to suggest there is a causal relationship (e.g. the event did not occur within a reasonable time after the trial procedure. There is another reasonable explanation for the event (e.g. the patient’s clinical condition, other concomitant treatment)
Possible	There is some evidence to suggest a causal relationship (e.g. because the event occurs within a reasonable time after the trial procedure. However, the influence of other factors may have contributed to the event (e.g. the patient’s clinical condition, other concomitant treatments)
Probable	There is evidence to suggest a causal relationship, and the influence of other factors is unlikely
Definitely	There is clear evidence to suggest a causal relationship, and other possible contributing factors can be ruled out
Not assessable	There is insufficient or incomplete evidence to make a clinical judgement of the causal relationship.

12.4. Related Unexpected SAE

A serious adverse reaction that is assessed by the Chief Investigator or nominative representative as unexpected.

12.5. Reporting Adverse Events to ICR-CTSU

Any toxicity, sign or symptom that occurs after randomisation should be considered an AE.

All AEs must be reported on the relevant toxicity, sign or symptom CRF.

The severity of AEs should be graded according to CTCAE v4 criteria. For each toxicity/sign/symptom, the highest grade observed since the last visit should be reported.

Whenever one or more toxicity/sign/symptom corresponds to a disease or a well-defined syndrome only the main disease/syndrome should be reported.

Hafeez S, *et al. BMJ Open* 2020; 10:e037134. doi: 10.1136/bmjopen-2020-037134

12.6. Reporting Serious Adverse Events to ICR-CTSU

Any SAE (except those listed below) that occurs from the start of radiotherapy and up to 30 days following the last day of radiotherapy must be reported.

In addition, any non-genitourinary adverse event grade 3 or higher (CTCAE v.4) and occurring up to 3 months after completion of radiotherapy must be reported.

All SAEs should be reported to ICR-CTSU within 24 hours of the Principal Investigator (or designated representative) becoming aware of the event, by completing the HYBRID SAE form and faxing to:

The ICR-CTSU safety desk
Fax no: **0208 722 4368**
For the attention of the HYBRID Trial team

As much information as possible, including the Principal Investigator's assessment of causality, must be reported to ICR-CTSU in the first instance. Additional follow up information should be reported as soon as it is available.

All SAE forms must be completed, signed and dated by the Principal Investigator or designated representative.

The Site SAE log should be completed and the SAE form filed in the Site Investigator File.

12.7. Serious Adverse Events exempt from expedited reporting

12.7.1. Expected genitourinary events:

The following adverse events are exempt from expedited reporting if grade **≤3**:

- Haematuria
- Dysuria/frequency
- Bladder spasms or pain
- Urinary tract infection
- Urinary/clot retention

12.7.2. Expected non-genitourinary events

The following adverse events are exempt from expedited reporting if grade **≤2**:

- Admission for transfusion secondary to bleeding from bladder tumour or anaemiaNausea/vomitingDiarrhoea

12.8. Abdominal painReview of Serious Adverse Events

The Chief Investigator (or designated representative) will assess all reported SAEs for causality and expectedness (NB. The Chief Investigator cannot down-grade the Principal Investigator's assessment of causality.)

Sites should respond as soon as possible to requests from the Chief Investigator or designated representative (via ICR-CTSU) for further information that may be required for final assessment of an SAE.

12.9. Expedited Reporting of Related Unexpected SAEs

If an SAE is identified as being related and unexpected by the Chief Investigator it will be reported by ICR-CTSU to the main REC within 15 days of being notified of the event.

The Principal Investigators at all actively recruiting sites will be informed of any related unexpected SAEs occurring within the trial at regular intervals.

12.10. Follow up of Serious Adverse Events

SAEs should be followed up until clinical recovery is complete or until the condition has stabilised. SAE outcomes should be reported to ICR-CTSU using the relevant section of the SAE form as soon as the Principal Investigator becomes aware of the outcome.

12.11. Annual reporting of safety considerations

Any safety concerns will be reported to the main REC by ICR-CTSU as part of the annual progress report at the end of the reporting year. This will be defined as the anniversary of the date when the study received a favourable opinion from the Main REC.

13. STATISTICAL CONSIDERATIONS

13.1. Statistical design and sample size justification

The sample size is based on the primary endpoint of acute non-genitourinary grade 3 or greater toxicity (toxicity being defined as adverse events related to study treatment). An A'Hern exact phase II design has been used to rule out an upper limit of toxicity for each planning method separately. Using data from the APPLY study it is expected that the acute non-genitourinary CTCAE grade 3+ rate will be 10% in patients receiving adaptive planning. The study has been designed to rule out a 30% upper limit of grade 3+ non-genitourinary toxicity within each planning method. With 80% power and 5% alpha (one-sided) this requires 28 patients in each group. If 5 or more patients within either planning method experience a non-GU grade 3+ toxicity then toxicity will be assumed to be too high. To be assessable for acute toxicity patients are required to have received at least 1 fraction of radiotherapy. If any patients stop treatment early for a reason clearly unrelated to toxicity the TSC will advise on evaluability. Recruitment will continue until there are 28 evaluable patients in each planning method group. A 10% non-evaluable rate has been accounted for in the target sample size of 62 patients. Recruitment beyond 62 patients, if more than 10% are non-evaluable, will be reviewed by the TSC.

There are a number of important secondary endpoints which the study will have sufficient statistical power to address. The overall toxicity of a 6Gy 6 fraction schedule will be assessed by combining the standard and adaptive planning groups. Using data from BC2001 and the APPLY study it is estimated that the any grade 3+ acute toxicity rate will be 25%. With 62 patients, there will be 80% power (5% alpha) to rule out an upper limit of 40% any grade 3+ acute toxicity with hypofractionated radiotherapy.

Local disease control will be assessed by combining the standard and adaptive planning groups to assess the effectiveness or otherwise of 36Gy/6f. The expected rate of local disease control at 3 months is 60%. If the true rate is 40% weekly hypofractionated RT would be judged ineffective. With 62 patients there is 87% power (5% one-sided alpha) to rule out less than 40% control rate, allowing for 25% non-evaluable patients at 3 months.

In pilot studies it is estimated that about 50% of treatments would benefit from adaption (either smaller or larger margins than standard). If >25% of all fractions treated or if >1fraction/patient require intervention, we anticipate clinical benefit for online correction. To determine a rate of less than 25% if the true rate is 50% with 86% power would require 29 patients to be treated in the adaptive planning group.

Another key secondary endpoint is the appropriate identification and correction of fractions requiring adaptive planning. The sample size provides sufficient power to investigate this question. If the true agreement between online and offline protocols is 85% and we wish to prove it is >75%, using a single-stage exact binomial phase II design, then for 90% power 139 fractions are required. If each patient receives 5 fractions on average, then 28 patients are needed. There is some allowance for the possibility that patients receive fewer than 5 fractions with 31 patients receiving adaptive planning (average treatment of 4.5 fractions each).

13.2. Treatment allocation

Participants will be randomised between standard and adaptive treatment delivery on a 1:1 basis.

Treatment allocation is by minimisation with a random element; balancing factors will be listed in the statistical analysis plan.

13.3. Primary endpoint definition

Acute non genitourinary toxicity will be assessed and reported using the CTCAE toxicity scale. Any grade 3 or greater treatment-related toxicities occurring within the first 3 months of radiotherapy completing will be included in assessment of the primary endpoint. Relatedness to study treatment will be subject to independent review blinded to planning method. If there are discrepancies between the PI, CI and independent assessment of relatedness, the independent assessment will take precedent for the purpose of enumeration of primary endpoint events. To be assessable for acute toxicity patients are required to have received at least 1 fraction of radiotherapy.

13.4. Secondary endpoint definitions

13.4.1. Local disease control at 3 months

This will be assessed by general anaesthetic cystoscopy and with a tumour bed biopsy at 3 months. Patients will be classified as either having evidence of residual tumour at 3 months or not. Data will be presented for all patients combined, regardless of treatment allocation. Patients will only be included in the denominator if they were able to have a disease assessment at 3 months.

13.4.2. Control rate of presenting symptoms

This will be assessed by looking at change in symptom scores from pre to post radiotherapy. Pre-radiotherapy symptoms will be recorded using the CTCAE toxicity scale and compared with CTCAE scores at 3 months from the completion of radiotherapy. The number of patients with post-radiotherapy scores equal to or lower than their baseline score will be used to calculate the control rate of presenting symptoms. Data will be presented separately for the two randomisation groups

13.4.3. Patient reported outcomes (PRO)

This will be assessed using the modified Inflammatory Bowel Disease Questionnaire (IBDQ), the King's Health Questionnaire and the EQ5D. PRO will be assessed pre-radiotherapy, at fraction 6 and, 3 and 6 months from completion of radiotherapy. The IBDQ bowel-related symptoms at 3 months from completion of radiotherapy are of primary interest. Data will be presented separately for the two randomisation groups.

13.4.4. Late toxicity

This will be assessed using CTCAE and RTOG scoring criteria at 6, 12 and 24 months from completion of radiotherapy. Data will be presented separately for the two randomisation groups.

13.4.5. Time to local disease progression

This will be measured from randomisation to the first occurrence of local disease as identified by cystoscopy or cytology and CT.

Hafeez S, *et al. BMJ Open* 2020; 10:e037134. doi: 10.1136/bmjopen-2020-037134

13.4.6. Overall survival

This will include deaths from any cause and time will be measured from randomisation

13.4.7. Proportion of fractions benefiting from adaptive planning

This will be assessed by the number of small or large plans being selected rather than the medium plan for patients in the adaptive planning group. The denominator will be the total number of fractions received in the adaptive planning group.

13.4.8. Appropriate identification and correction of fractions requiring adaptive planning

This will require an independent reviewer to select an appropriate plan for each fraction for each patient. The concordance between the actual and independent reviewer plan selection will be presented.

13.5. Exploratory endpoints

Dose Volume Histogram analysis of CTV coverage using anisotropic margins will be assessed.

The level of agreement between clinician reported toxicity and PRO will also be explored at each time point where both assessments are used.

13.6. Analysis plan

The numbers and proportions of patients with acute non-genitourinary CTCAE v4 toxicity grade 3 or greater in each planning method will be presented together with 95% one-sided exact confidence intervals (the 90% two-sided confidence interval will also be presented). The primary analysis population will be patients who received 1 fraction of radiotherapy. Sensitivity analyses will also be conducted including all randomised patients in the denominator and also only patients who received at least 3 fractions of radiotherapy.

Acute toxicity figures will also be presented by planning method for any grade 3 or greater non-genitourinary adverse event (i.e. including treatment related and not), and for any genitourinary grade 3 or greater adverse event (treatment related and all events) and any grade 3 or greater adverse event (treatment related and all events) for the two planning methods combined. The distribution of non-genitourinary and genitourinary adverse events/toxicity will be presented as numbers and proportion at each time point they are assessed.

The local control rate at 3 months will be presented for all patients (standard and adaptive combined) together with a 95% confidence interval (CI). The local control rate will be presented as a proportion with patients only included in the denominator if they were able to have an assessment at 3 months. The principal analysis will be based on all data reported on the 3 month assessment form will be used with no restrictions on time windows.

Late toxicity will be summarised by frequencies and proportions at each time point by treatment group. Kaplan-Meier methods may be used to present time-to-event data e.g. time to first occurrence of a grade 2 or greater event.

Kaplan-Meier methods will be used to analyse time to local disease progression and overall survival with data presented for randomised groups combined. Data will not be presented by treatment group as there is insufficient statistical power to detect clinically meaningful differences and this is not the aim of this phase II study. Time will be measured from randomisation and patients with no event will be censored on the data of last clinical assessment.

The proportion of fractions requiring clinical intervention will be calculated as the number out of all fractions treated, and reported with an exact binomial confidence interval.

PRO scores will be generated by combining individual items to produce subscale scores for each domain of the questionnaires using standard algorithms. The frequency and percentages at each time point will be presented for each PRO questionnaire. Data will be presented separately for each randomised group.

Pre-planned exploratory sub-group analyses will be conducted to investigate potential differences in toxicity in the following sub-groups, if there are sufficient numbers within each subgroup:

- Histology: TCC vs any other tumour types
- Staging: T2 vs T3/T4
- Performance status: 0/1 vs 2/3

Further details of analysis methods will be specified in a Statistical Analysis Plan in accordance with ICR-CTSU Standard Operating Procedures.

13.7. Interim analyses and stopping rules

This is a phase II trial and there will be no formal early stopping rules for efficacy or toxicity. As per the single stage design, if 5 or more patients report treatment related non-genitourinary grade 3 or greater acute toxicities in one planning group then randomisation will be suspended and the Independent Data Monitoring Committee (IDMC) will review the data and be asked to advise on continuation of recruitment to both planning groups.

The IDMC will closely monitor accumulating safety and efficacy data at regular intervals (to be determined by recruitment rates but likely to be 6-12 monthly) and will advise if there are any safety signals. An initial safety review will take place when 3 month data are available for 5 patients (who have received at least 3 fractions of RT) in each group.

14. TRIAL MANAGEMENT

14.1. Trial Management Group (TMG)

A Trial Management Group (TMG) will be set up and will include the Chief Investigator, ICR-CTSU Scientific Lead, Co-investigators and identified collaborators, the Trial Statistician and Trial Manager. Principal Investigators and key study personnel will be invited to join the TMG as appropriate to ensure representation from a range of sites and professional groups. Where possible, membership will include a lay/consumer representative. The TMG will meet at regular intervals, and at least annually. Notwithstanding the legal obligations of the sponsor and Chief Investigator, the TMG have operational responsibility for the conduct of the trial. The Committee's terms of reference, roles and responsibilities will be defined in a charter issued by ICR-CTSU.

14.2. Trial Steering Committee (TSC)

The HYBRID trial will be overseen by the ICR-CTSU Bladder Trials Steering Committee (TSC) which includes an independent Chairman (not involved directly in the trial other than as a member of the TSC) and not less than two other independent members. The TSC will meet annually. The TSC will provide expert independent oversight of the trial on behalf of the sponsor and funder. The Committee's terms of reference, roles and responsibilities will be defined in charter issued by ICR-CTSU.

14.3. Independent Data Monitoring Committee (IDMC)

An IDMC will be instigated to monitor the progress of the trial and will include at least three independent members, one of whom will be a medical statistician. The Committee's terms of reference, roles and responsibilities will be defined in a charter issued by ICR-CTSU. In the absence of a separate independent assessor(s), a clinical member(s) of the IDMC will perform the

independent review of relatedness to study treatment of grade 3 or greater acute non-genitourinary adverse events.

The IDMC will meet in confidence at regular intervals, and at least annually. A summary of findings and any recommendations will be produced following each meeting. This summary will be submitted to the TMG and TSC, and if required, the main REC.

The IDMC reserve the right to release any data on outcome or side-effects through the TSC to the TMG (and if appropriate to participants) if it determines at any stage that the combined evidence from this and other studies justifies it.

15. RESEARCH GOVERNANCE

15.1. Sponsor responsibilities

The sponsor of this clinical trial is the Institute of Cancer Research (ICR).

Responsibilities of participating sites are defined in an agreement between the individual participating site and the Sponsor

16. TRIAL ADMINISTRATION & LOGISTICS

16.1. Site activation

Before activating the trial, participating sites are required to sign an agreement accepting responsibility for all trial activity which takes place within their site.

Sites may commence recruitment once the site agreement has been signed by all required signatories, the required trial documentation is in place (as specified by ICR-CTSU) and a site initiation (visit or teleconference) has taken place. Site initiation visits will be conducted at sites where the Principal Investigator has requested one or where ICR-CTSU deems it is appropriate.

16.2. Investigator training

Each centre will complete the comprehensive pre-trial section of the quality assurance programme prior to commencing recruitment, as detailed in section 12. In addition to this, prior to trial initiation, a practical workshop will be held to educate Principal Investigators, radiographers and physicists in adaptive radiotherapy techniques. The quality assurance programme will continue throughout the trial, with investigator training as required.

16.3. Data acquisition

Electronic (e) Case Report Forms (CRF) will be used for the collection of trial data. ICR-CTSU will provide guidance to sites to aid the completion of the eCRFs. The Trial Management Group reserves the right to amend or add to the eCRF template as appropriate. Such changes do not constitute a protocol amendment, and revised or additional forms should be used by sites in accordance with the guidelines provided by ICR-CTSU.

16.4. Central data monitoring

Once data has been entered on the eCRF by the site personnel, ICR-CTSU will review it for compliance with the protocol, and for inconsistent or missing data. Should any missing data or data anomalies be found, queries will be raised for resolution by the site.

Any systematic inconsistencies identified through central data monitoring may trigger an on-site monitoring visit.

16.5. On-site monitoring

If a monitoring visit is required, ICR-CTSU will contact the site to arrange the visit. Once a date has been confirmed, the site should ensure that full patient notes of participants selected for source data verification are available for monitoring.

ICR-CTSU staff conducting on-site monitoring will review essential documentation and carry out source data verification to confirm compliance with the clinical trial agreement and trial protocol. If any problems are detected during the course of the monitoring visit, ICR-CTSU will work with the Principal Investigator or delegated individual to resolve issues and determine appropriate action.

16.6. Completion of the study and definition of study end date

The study end date is deemed to be the date of last data capture.

16.7. Archiving

Essential trial documents should be retained according to local policy and for a sufficient period for possible inspection by the regulatory authorities (at least 5 years after the date of last data capture). Documents should be securely stored and access restricted to authorised personnel.

17. PATIENT PROTECTION AND ETHICAL CONSIDERATIONS

17.1. Trial approvals

This trial has been formally assessed for risk by ICR-CTSU.

The trial has received ethical approval from a research ethics committee for multi-centre trials and global R&D approval via the NIHR Coordinated System for gaining NHS Permission. Before entering patients, the Principal Investigator at each site is responsible for submitting Site Specific Information and gaining local Research and Development approval of this protocol.

17.2. Trial conduct

This trial will be conducted in accordance with the Research Governance Framework for Health and Social Care and the principles of GCP.

17.3. Informed consent

Patients should be asked to sign the current main REC approved HYBRID consent form at trial entry after receiving both verbal and written information about the trial, having been given sufficient time to consider this information. All consent forms must be countersigned by the Principal Investigator or a designated individual. A signature log of delegated responsibilities, listing the designated individuals and the circumstances under which they may countersign consent forms, must be maintained at the participating site. This log, together with original copies of all signed patient consent forms, should be retained in the Site Investigator File and must be available for inspection. The current main REC approved HYBRID patient information sheets should be provided in addition to any standard patient information sheets that are provided by the site and used in routine practice.

17.4. Patient confidentiality

Patients will be asked to consent to their full name being collected at registration in addition to their date of birth, hospital number, postcode and NHS number or equivalent to allow linkage with routinely collected NHS data.

Each investigator should keep a separate log of all participants' Trial IDs, names, addresses and hospital numbers. The investigator must retain trial documents (e.g. participants' written consent forms) in strict confidence. The investigator must ensure the participants' confidentiality is maintained at all times.

Representatives of the sponsor, ICR-CTSU, other coinvestigators, members of the hospital R&D team and regulatory authorities may require access to participants' notes for quality assurance and audit purposes. ICR-CTSU will maintain the confidentiality of participants at all times and will not reproduce or disclose any information by which participants could be identified.

17.5. Data Protection Act (DPA)

ICR-CTSU will comply with all aspects of the DPA 1998. Any requests from participants for access to their data held at ICR-CTSU will be referred to the Data Protection Officer at the ICR.

17.6. Liability

Indemnity for participating hospitals is provided by the usual NHS indemnity arrangements. Inclusion of private patients will be subject to the site ensuring appropriate insurance and indemnity arrangements are in place.

18. FINANCIAL MATTERS

This trial is investigator designed and led and has been approved by the Clinical Trials Advisory & Awards Committee (CTAAC) of Cancer Research UK.

ICR has received funding from Cancer Research UK for the central coordination of the trial. In the UK, the trial meets the criteria for R&D support as outlined in the Statement of Partnership on Non-Commercial R&D in the NHS in England. The trial is part of the National Institute for Health Research (NIHR) portfolio. Research Network resources should therefore be made available for the trial to cover UK specific research costs.

19. PUBLICATION POLICY

The main trial results will be published in a peer-reviewed journal, on behalf of all collaborators. The manuscript will be prepared by a writing group, consisting of members of the TMG and selected participating clinicians. All participating clinicians will be acknowledged in the publication.

Any presentations and publications relating to the trial must be authorised by the TMG. Authorship of any secondary publications e.g. those relating to sub-studies, will reflect the intellectual and time input into these studies.

No investigator may present or attempt to publish data relating to the HYBRID trial without prior permission from the TMG.

20. ASSOCIATED STUDIES

20.1. Patient reported outcome measures study

Patient reported outcomes will be a secondary endpoint in the main trial and will be analysed as described in the statistical analysis plan.

Further details are provided in Appendix 3.

21. REFERENCES

1 UK CR. Bladder cancer; 2013 Jan 2013.
2 Rodel C. Combined-Modality Treatment and Selective Organ Preservation in Invasive Bladder Cancer: Long-Term Results. Journal of Clinical Oncology. 2002;20(14):3061-71.
3 NICE. Guidance on Cancer Services Improving Outcomes in Urological Cancers The Manual. online: NICE; 2002.
4 Kotwal S, Choudhury A, Johnston C, Paul AB, Whelan P, Kiltie AE. Similar treatment outcomes for radical cystectomy and radical radiotherapy in invasive bladder cancer treated at a United Kingdom specialist treatment center. Int J Radiat Oncol Biol Phys. 2008 Feb 1;70(2):456-63.
5 Chahal R, Sundaram SK, Iddenden R, Forman DF, Weston PM, Harrison SC. A study of the morbidity, mortality and long-term survival following radical cystectomy and radical radiotherapy in the treatment of invasive bladder cancer in Yorkshire. Eur Urol. 2003 Mar;43(3):246-57.
6 NCIN. Cancer Incidence and Mortality By Cancer Network, UK, 2005. 2010.
7 Dunst J, Diestelhorst A, Kuhn R, Muller AC, Scholz HJ, Fornara P. Organ-sparing treatment in muscle-invasive bladder cancer. Strahlenther Onkol. 2005 Oct;181(10):632-7.
8 Shipley WU, Kaufman DS, Zehr E, et al. Selective bladder preservation by combined modality protocol treatment: long-term outcomes of 190 patients with invasive bladder cancer. Urology. 2002 Jul;60(1):62-7; discussion 7-8.
9 Huddart R, Norman A, Shahidi M, Dearnaley D, Horwich A. The effect of reducing the high dose volume on toxicity of radiotherapy in the treatment of bladder cancer. British Journal of Cancer. 1999;80(Supplement 2):A3.10.
10 Huddart RA, Hall E, Lewis R, Birtle A. Life and death of spare (selective bladder preservation against radical excision): reflections on why the spare trial closed. BJU Int. 2010 Sep;106(6):753-5.
11 Duchesne GM, Bolger JJ, Griffiths GO, et al. A randomized trial of hypofractionated schedules of palliative radiotherapy in the management of bladder carcinoma: results of medical research council trial BA09. Int J Radiat Oncol Biol Phys. 2000 May 1;47(2):379-88.
12 Jose CC, Price A, Norman A, et al. Hypofractionated radiotherapy for patients with carcinoma of the bladder. Clin Oncol (R Coll Radiol). 1999;11(5):330-3.
13 McLaren DB, Morrey D, Mason MD. Hypofractionated radiotherapy for muscle invasive bladder cancer in the elderly. Radiother Oncol. 1997 May;43(2):171-4.
14 Rostom AY, Tahir S, Gershuny AR, Kandil A, Folkes A, White WF. Once weekly irradiation for carcinoma of the bladder. Int J Radiat Oncol Biol Phys. 1996 May 1;35(2):289-92.
15 Fokdal L, Honore H, Hoyer M, Meldgaard P, Fode K, von der Maase H. Impact of changes in bladder and rectal filling volume on organ motion and dose distribution of the bladder in radiotherapy for urinary bladder cancer. Int J Radiat Oncol Biol Phys. 2004 Jun 1;59(2):436-44.
16 Lotz HT, Pos FJ, Hulshof MC, et al. Tumor motion and deformation during external radiotherapy of bladder cancer. Int J Radiat Oncol Biol Phys. 2006 Apr 1;64(5):1551-8.
17 Meijer GJ, Rasch C, Remeijer P, Lebesque JV. Three-dimensional analysis of delineation errors, setup errors, and organ motion during radiotherapy of bladder cancer. Int J Radiat Oncol Biol Phys. 2003 Apr 1;55(5):1277-87.
18 Muren LP, Smaaland R, Dahl O. Organ motion, set-up variation and treatment margins in radical radiotherapy of urinary bladder cancer. Radiother Oncol. 2003 Dec;69(3):291-304.
19 Sur RK, Clinkard J, Jones WG, et al. Changes in target volume during radiotherapy treatment of invasive bladder carcinoma. Clin Oncol (R Coll Radiol). 1993;5(1):30-3.
20 Turner SL, Swindell R, Bowl N, et al. Bladder movement during radiation therapy for bladder cancer: implications for treatment planning. Int J Radiat Oncol Biol Phys. 1997 Sep 1;39(2):355-60.
21 Pos F, Remeijer P. Adaptive management of bladder cancer radiotherapy. Semin Radiat Oncol. 2010 Apr;20(2):116-20.
22 Lalondrelle S, Huddart R. Improving radiotherapy for bladder cancer: an opportunity to integrate new technologies. Clin Oncol (R Coll Radiol). 2009 Jun;21(5):380-4.

23 Lalondrelle S, Huddart R, Warren-Oseni K, et al. Adaptive-predictive organ localization using cone-beam computed tomography for improved accuracy in external beam radiotherapy for bladder cancer. Int J Radiat Oncol Biol Phys. 2011 Mar 1;79(3):705-12.
24 National Radiotherapy Implementation Group Report Image Guided Radiotherapy (IGRT) Guidance for implementation and use.; 2012.
25 McDonald F, Lallondrelle S, Taylor H, et al. Clinical implementation of adaptive hypo-fractionated bladder radiotherapy for improvement in normal tissue irradiation. Submitted for publication. 2013.
26 McDonald F, Lalondrelle S, Taylor H, et al. Adaptive-predictive organ localisation [A-POLO] in the clinic: Updated results in hypo-fractionated bladder radiotherapy GU ASCO; 2011; 2011.
27 Andreyev HJ, Wotherspoon A, Denham JW, Hauer-Jensen M. Defining pelvic-radiation disease for the survivorship era. Lancet Oncol. Apr;11(4):310-2.
28 Guyatt G, Mitchell A, Irvine EJ, et al. A new measure of health status for clinical trials in inflammatory bowel disease. Gastroenterology. 1989 Mar;96(3):804-10.
29 Olopade FA, Norman A, Blake P, et al. A modified Inflammatory Bowel Disease questionnaire and the Vaizey Incontinence questionnaire are simple ways to identify patients with significant gastrointestinal symptoms after pelvic radiotherapy. Br J Cancer. 2005 May 9;92(9):1663-70.
30 Wedlake LJ, Thomas K, Lalji A, et al. Predicting Late Effects of Pelvic Radiotherapy: Is There a Better Approach? Int J Radiat Oncol Biol Phys. 2010 Mar 16:March 16.
31 Reese P, Pleil A, Okano G, Kelleher C. Multinational study of reliability and validity of the King's Health Questionnaire in patients with overactive bladder. Qual Life Res. 2003 2003/06/01;12(4):427-42.
32 Brooks R. EuroQol: the current state of play. Health policy (Amsterdam, Netherlands). 1996 Jul;37(1):53-72.

A1. WHO performance status

Grade	Performance Status
0	Able to carry out all normal activity without restriction.
1	Restricted in physically strenuous activity but ambulatory and able to carry out light work.
2	Ambulatory and capable of all self-care but unable to carry out any work; up and about more than 50% of waking hours.
3	Capable of only limited self-care; confined to bed or chair more than 50% of waking hours.
4	Completely disabled; cannot carry on any self-care; totally confined to bed or chair.

A2. RTOG/EORTC late radiation morbidity scoring schema

0	1	2	3	4	5
BLADDER					
None	Slight epithelial atrophy<br>Minor telangiectasia (microscopic haematuria)	Moderate frequency<br>Generalized telangiectasia<br>Intermittent macroscopic haematuria	Severe frequency and dysuria<br>Severe generalized telangiectasia (often with petechiae)<br>Frequent haematuria<br>Reduction in bladder capacity (<150 cc)	Necrosis/ Contracted bladder (capacity <100 cc)<br>Severe haemorrhagic cystitis	Death due to toxicity
SMALL/LARGE INTESTINE					
None	Mild diarrhoea<br>Mild cramping<br>Bowel movement 5 times daily<br>Slight rectal discharge or bleeding	Moderate diarrhoea and colic<br>Bowel movement >5 times daily<br>Excessive rectal mucus or intermittent bleeding	Obstruction or bleeding requiring surgery	Necrosis/ Perforation<br>Fistula	Death due to toxicity

Hafeez S, *et al. BMJ Open* 2020; 10:e037134. doi: 10.1136/bmjopen-2020-037134

A3. PATIENT REPORTED OUTCOMES STUDY

A3.1 Background

The primary endpoint of HYBRID is clinician reported acute non-genitourinary toxicity within 3 months from completion of radiotherapy.

Patient reported outcomes (PRO) are a key secondary endpoint within HYBRID. The aim will be to collect detailed information about the impact of hypofractionated bladder radiotherapy on participants' daily lives, with a focus on side effects being experienced but also including a measure of general wellbeing.

The objective of the PRO sub-study within HYBRID is to compare the impact of adaptive planned hypofractionated radiotherapy on side effects as reported by the participants. This will help to support any differences in toxicity established within the primary endpoint of clinician reported toxicity. In addition, PRO data will be compared with clinician reported toxicity to give an indication of the concordance of the two measures.

A3.2 Hypothesis

It is hypothesised that participants in the adaptive planning group will report fewer severe non-genitourinary toxicities than those in the standard planning group.

A3.3 Quality of life measures

Patient reported outcomes will be measured using the modified Inflammatory Bowel Disease Questionnaire (IBDQ), King's Health Questionnaire (KHQ) and EQ-5D.

Lower gastrointestinal symptoms caused by radiation induced toxicity are similar to those in common bowel disorders unrelated to cancer therapies (27). The modified IBDQ (28) is the preferred patient reported measurement tools used by gastroenterologists specialising in pelvic radiotherapy-related side-effects (29). The modified IBDQ is a 32-item questionnaire consisting of four dimensions: bowel-related symptoms, systematic function (e.g. fatigue, sleep pattern), social function (e.g. ability to attend work and social events) and emotional status (e.g. anger, depression, irritability). The bowel subset of the modified IBDQ, the 'IBDQ-B' questionnaire has been used previously in a specialist service evaluating radiotherapy-related gastrointestinal side-effects. These patient reported questionnaires are easy to complete and are a sensitive indicator of radiotherapy toxicity (30).

Urinary side-effects experienced by participants will be captured using the KHQ, which has been validated for use in patients with overactive bladder (31) and captures details of the severity of symptoms and the impact of urinary incontinence on day to day living.

Participants will also be asked to complete the EQ5D questionnaire, a brief standardised instrument which provides a simple descriptive profile of health status (32).

A3.4 Study design

Patients are eligible for the PRO study if they fulfil the HYBRID eligibility criteria. Participants will be asked in the patient information sheet to consent to regular completion of PRO questionnaires. Patients who decline to take part in the HYBRID PRO study will remain eligible for the main trial. PRO is a secondary endpoint in the main trial and the primary timepoint of interest is 3 months after completion of radiotherapy.

A3.5 Timing of data collection

Participants will be asked to complete a questionnaire in clinic within two weeks prior to the start of treatment. Further questionnaires will be completed in clinic, at fraction six of treatment delivery and 3 and 6 months from the end of treatment.

A3.6 Compliance

Missing data may hamper interpretation of PRO. Missing data may arise because participants do not complete the questionnaires at the appropriate time (unit non-response), or because patients may miss questions within the questionnaires (item non-response). In a population of patients with low performance status such as those included in HYBRID, there is potential for non-response and informative censoring (with data not missing at random). During the study, compliance with PRO questionnaire completion will be monitored by the trial oversight committees.

A3.7 Statistical considerations

Patient reported outcome analyses will be used to supplement results of clinician assessed treatment toxicity, therefore a formal sample size calculation has not been performed. An analysis plan will be developed in consultation with the TMG with key endpoints identified from each questionnaire. Standard algorithms will be used to derive scores and handle missing data in quality of life questionnaires. Quality of life data will be presented at individual time-points and analyses to account for the longitudinal nature of the data may be used.

A4. GLOSSARY

AE	Adverse Event
APPLY	Adaptive predictive planning for hypofractionated bladder radiotherapy
CBCT	Cone beam CT
CI	Chief Investigator
CI	Confidence interval
CIS	Carcinoma in Situ
CRF	Case Report Form
CT	Computed tomography
CTCAE	Common Terminology Criteria for Adverse Events
CTV	Clinical target volume
DCF	Data Capture Form
DVH	Dose Volume Histogram
EORTC	European Organisation for Research and Treatment of Cancer
f	Fraction
FBC	Full Blood Count
GI	Gastrointestinal
GTV	Gross tumour volume
GU	Genitourinary
Gy	Gray
HR	Hazard Ratio
IBDQ	Inflammatory Bowel Disease Questionnaire
ICR	The Institute of Cancer Research
ICR-CTSU	The Institute of Cancer Research Clinical Trials and Statistics Unit
IDMC	Independent Data Monitoring Committee
IGRT	Image guided radiotherapy
KHQ	King’s Health Questionnaire
MDT	Multi-disciplinary team
MIBC	Muscle invasive bladder cancer
MRI	Magnetic resonance imaging
NCRI	National Cancer Research Institute
NCRI RTTQA	NCRI Radiotherapy Clinical Trials Quality Assurance group
NICE	National Institute for Health and Clinical Excellence
NSAID	Non-steroidal anti-inflammatory drug
PI	Principal Investigator
PIS	Patient Information Sheet
PRO	Patient Reported Outcomes
PTV	Planning target volume
QA	Quality assurance
R&D	Research and Development
REC	Research Ethics Committee
RMH	Royal Marsden Hospital
RT	Radiotherapy
RTOG	Radiation Therapy Oncology Group
SAE	Serious Adverse Event
SAR	Serious Adverse Reaction
TMG	Trial Management Group
TSC	Trial Steering Committee
WHO	World Health Organisation

This protocol is a controlled document and should not be copied, distributed or reproduced without the written permission of the ICR-CTSU
