# Supplementary material for: Protocol for hypofractionated adaptive radiotherapy to the bladder within a multicentre phase II randomised trial: radiotherapy planning and delivery guidance
Source: BMJ Open. 2020 May 26;10(5):e037134. doi: 10.1136/bmjopen-2020-037134 (PMC7259864; doi:10.1136/bmjopen-2020-037134)
Supplement: Supplementary data [file bmjopen-2020-037134supp002.pdf]

Please print on hospital headed paper

HYBRID

A multicentre randomised phase II study of **Hy**pofractionated **B**ladder **R**adiotherapy with or without **I**mage guided a**d**aptive-predictive planning

PATIENT INFORMATION SHEET
Version 1.1; 24 September 2013

We would like to invite you to take part in a research study called HYBRID.

Before you decide whether to take part, it is important that you understand why the research is being done and what it would involve for you. One of your doctors or nurses will go through this information sheet with you and answer any questions you may have . Please take time to read the information carefully and to discuss it with relatives, friends and your GP if you wish. Please ask if anything is unclear or you need any further information.

Thank you for reading this and considering taking part in this research.

Why am I being invited to take part?
We are inviting you to join this study because your doctor has found cancer that has grown into the wall of your bladder. Treatment for this type of bladder cancer would usually be surgery to remove the bladder or radiotherapy given in small doses every day for 4 or 7 weeks.

In your case, your doctor does not think that major surgery is a suitable treatment for you and you have agreed with your doctor that coming to hospital for radiotherapy treatment every day for 4 or 7 weeks would be difficult. As an alternative s/he is recommending that you are treated with radiotherapy given once a week for six weeks.

What is radiotherapy treatment?
Radiotherapy uses targeted beams of high strength x-rays to kill cancer cells. Because radiotherapy can also cause damage to normal, non-cancer cells, the treatment is carefully planned by doctors and physicists at your hospital so that only your bladder and a small border surrounding it is exposed to the highest radiotherapy dose.

Radiotherapy treatment is individually designed for each patient, based on a CT scan taken a few weeks before treatment which tells us about the position and shape of the bladder. The bladder can move about within the body depending on how full it is and because of where it is in relation to the bowel. It is important that the radiotherapy does not miss any of the bladder because of this movement, so we add a safety margin around the bladder on the planned treatment, to reduce the risk of the highest doses of radiotherapy missing any of the bladder.

Each patient would usually have just one radiotherapy plan designed for them before they start treatment. When radiotherapy treatment is given, the patient has to lie still on a bed whilst the radiotherapy machine moves around to send the radiotherapy beams from

(Form to be printed on headed paper)

different directions. These beams all focus on where the bladder is, to make sure that it receives the highest radiotherapy dose possible.

What is adaptive radiotherapy treatment?
We are now able to take a scan of where the bladder is when a patient is lying on the radiotherapy bed before each treatment. This means that we can give radiotherapy more accurately. In this study we are looking at whether it is possible to design three treatment plans with different size safety margins and then choose the one that fits best for each particular patient on their treatment day. This is called 'adaptive radiotherapy'.

Adaptive radiotherapy may allow treatment to be given with smaller safety margins, resulting in reduced side effects. The bladder would still receive the highest radiotherapy dose, but reducing the amount of radiotherapy to the margins could reduce non-bladder side effects.

What is the purpose of HYBRID?
Many people with bladder cancer find daily radiotherapy for a number of weeks difficult to cope with. One radiotherapy treatment a week for six weeks may be a good option for people who would find it difficult to come to the hospital every day and this type of radiotherapy is already in use at most hospitals which are taking part in the HYBRID research study.

We hope to show that adaptive radiotherapy reduces non-bladder side effects compared to when radiotherapy is given using the same plan each time. We also want to gather more information about how well bladder cancer is treated by weekly radiotherapy, and how well it reduces any symptoms patients experience as a result of bladder cancer.

What would happen if I took part?
All participants in this study will be treated with 6 radiotherapy treatments given once weekly for 6 weeks.

Everyone who agrees to take part in this research study will be allocated at random to one of two groups. Half of the people taking part will receive weekly radiotherapy using the same treatment plan each time and half of participants will have weekly adaptive radiotherapy, using one of three plans. The only way to make sure that the two groups are as similar as possible is to have the treatment decided upon by chance: a process called randomisation. This process ensures that the treatments are compared fully and fairly.

If you agree to take part, your doctor or nurse will ring the research centre. The centre will then record your details and tell your specialist your treatment, which will be selected by chance, meaning you will have an equal chance of having either of the treatments. Whichever group you are in, you will be treated with the best possible care and will be monitored closely.

Both treatment groups will have a scan before radiotherapy treatment to make sure that the radiotherapy treatment will not miss any of the bladder.

<u>What do I have to do before my radiotherapy treatment?</u>
To make sure that your treatment is as effective as possible, it has to be carefully planned by your Doctor and other specialised staff (radiographers and physicists). It is a very

(Form to be printed on headed paper)

precise treatment and it is important that you are able to lie in exactly the same position for every treatment.

The planning session at the radiotherapy department usually takes place once and will last about 30 minutes. You will have a CT scan taken with your bladder empty. The radiographers will also take measurements from you that are needed for treatment planning. All of the planning procedures are part of the routine care for patients receiving bladder radiotherapy, so you will have them even if you choose not to take part in the HYBRID research study.

<u>What do I have to do during my radiotherapy treatment?</u>
Your treatment will be given once a week for six weeks. We will ask you to empty your bladder immediately before each treatment. Once the radiographer has helped you to get into position and made sure that you are comfortable, we will take a scan in the treatment room. This will take about 2 minutes.

For patients receiving the same treatment plan each time, this scan will be used to make sure that the bladder is in the area which will receive the highest dose of radiotherapy. If you are receiving adaptive radiotherapy we will use the information from the pre-treatment scan to study the bladder and choose the best plan to fit your bladder size and position. This will take around five to ten minutes. Once the plan has been selected by a specially trained doctor or radiographer and checked by a second trained person you will receive your radiotherapy treatment.

The treatment only takes a few minutes, but you will need to lie still for approximately 20 minutes whilst the machine moves around to deliver the radiotherapy from different angles. You will not feel anything, as it is similar to having an x-ray.

<u>How many times will I need to visit the hospital during and after my treatment?</u>
You will be seen regularly by your Doctor and/or nurse during and after treatment so that the side effects and effectiveness of the treatment can be measured.

- During your radiotherapy treatment you will be seen by your Doctor and/or nurse every week to record and treat any side effects that you may be experiencing and they will take a small sample of blood before treatment starts and on the second, fourth and sixth treatment visit.
- After your treatment you will be seen 4 weeks and 3, 6, 12 and 24 months after the end of your radiotherapy to record and treat any side effects, if present, and check how well the treatment has controlled your cancer. You will be asked to have a cystoscopy (inspection of your bladder with a telescope) to check your bladder 3, 6 and 12 months after your radiotherapy. If you are unable to have any of these tests we would ask that you have a CT scan and provide a sample of urine so your doctor can test it for the presence of cancer cells. If your cancer is found to have returned when these checks are done, your doctor will discuss available treatment options with you.

<u>Will I be asked to do anything else?</u>
The main reason we are carrying out the HYBRID study is to look at the side effects of the radiotherapy treatment. If you decide to take part in HYBRID, we would like you to complete short questionnaires to describe any side effects that you may experience.

Hafeez S, *et al. BMJ Open* 2020; 10:e037134. doi: 10.1136/bmjopen-2020-037134

(Form to be printed on headed paper)

This is an optional part of the study but completed questionnaires will help us to understand more about the side effects of this radiotherapy treatment from your point of view. Completing a questionnaire should take no longer than 20 minutes.

If you agree to take part, we will ask you to fill in a questionnaire before you start radiotherapy, at the end of your radiotherapy treatment and then twice more, at 3 and 6 months afterwards. We know from other patients that they feel such surveys are very important, but you do not have to complete them if you do not want to.

What are the possible side effects of treatment?
Patients who have radiotherapy commonly experience some side effects. These can occur in anyone receiving radiotherapy to the bladder whether or not they are in HYBRID. No one can predict whether you will have some, all or none of the side effects, or how severe they may be. They are usually mild and short lived but can sometimes be more serious. Please let your doctor or nurse know about any side effects that you are concerned about so they can advise you what to do. Their telephone numbers are at the end of this information sheet (p7). There is also 24 hour support available from your hospital, to provide access to immediate medical care in the event of any serious problems.

Not all people will experience all of these side effects and we can give you medications to treat any side effects that you do experience.

You will be able to carry out most of your normal activities during radiotherapy, but you may feel more tired than normal and may need to rest more.

Side effects can develop during radiotherapy that may include:
- diarrhoea (around 3 in 10 people)
- needing to urinate more often (around 3 in 10 people)
- bleeding, pain or discomfort on passing urine (around 2 in 10 people)
- passing stools more frequently or with pain (around 1 in 10 people)

Most people return to normal after radiotherapy but a few may develop long term effects. These are usually mild but can occasionally be serious and can require treatment.

Side effects which can develop after radiotherapy include:
- a need to urinate more often or more urgently (around 2 in 10 people)
- bowel changes due to scarring or bleeding (around 5 in 100 people)
- vaginal scarring (around 3 in 10 women)
- problems with getting and maintaining erections (around 2 in 10 men)
- infertility (around 5 in 10 people)

Do I have to take part?
No, it is up to you to decide whether to take part or not. If you decide to take part, you will be given this information sheet to keep and will be asked to sign a consent form. You are free to change your mind and withdraw from the study at any time without giving a reason. If you do choose to withdraw, your doctor will discuss with you the best treatment option available for you at that time.

Hafeez S, *et al. BMJ Open* 2020; 10:e037134. doi: 10.1136/bmjopen-2020-037134

(Form to be printed on headed paper)

What are the alternatives to this study?
Participation in this study will not affect the usual standard of care you receive. There are no standard recommended treatments for patients who cannot have daily radiotherapy or surgery for bladder cancer. If you do not take part in HYBRID your doctor will discuss any alternative options with you.

What are the possible benefits of taking part?
Weekly radiotherapy may be a more effective treatment for the cancer than any alternative treatments. Everyone in the study will receive a scan before their treatment. This may make the radiotherapy more accurate than if it was given without the scan. If you are in the adaptive radiotherapy group you will receive radiotherapy treatment with the smallest possible safety margin each time and this may reduce the risk of non-bladder side effects.

You may be seen at your hospital more often, and have more cystoscopies or CT scans after you finish radiotherapy than you would have had if you were not in HYBRID. Your doctor will explain whether this is the case at your hospital. This may be beneficial in that any side effects or return of the cancer can be found and treated more quickly than they would otherwise.

What are the possible disadvantages of taking part?
The effects of this type of weekly radiotherapy treatment are not completely known. Early studies suggest the side effects of the treatment are similar to daily treatment. One of the purposes of this study is to confirm these reports. It is possible that the side effects of weekly radiotherapy might be worse than for daily treatment, but this will be monitored for all HYBRID participants and the study will be stopped if people are experiencing bad side effects.

The selection and confirmation of a treatment plan will extend the length of each radiotherapy treatment by about 5 to 10 minutes for patients receiving adaptive radiotherapy.

If you will have more cystoscopies or CT scans after you finish radiotherapy than you would have if you were not in HYBRID you will need to attend hospital more often than you would otherwise. If you have more CT scans than you would if you did not take part in HYBRID you will be exposed to more radiation than you would otherwise.

Before participating you should consider if this will affect any insurance you have and seek advice if necessary.

How will confidentiality be maintained?
Your medical notes will be seen by authorised members of the research team at your hospital, so that they can collect information needed for the HYBRID study. When you join the study, your name, date of birth, postcode, hospital number and NHS or Community Health Index (CHI) number will be passed to the Institute of Cancer Research Clinical Trials and Statistics Unit (ICR-CTSU) where the study is being coordinated. You will be given a unique registration number, which will be used together with your initials and date of birth on forms that the research staff will send to the trials office. All information about you will be coded with the registration number and will be stored securely. It will be treated

(Form to be printed on headed paper)

as strictly confidential and nothing that might identify you will be revealed to any third party.

Scientific employees of ICR-CTSU, and those conducting the study with them, including the national radiotherapy quality assurance team, may need to examine your medical records to ensure the study is being run properly and that the information collected on the forms is correct, but your confidentiality will be protected at all times.

We will contact your hospital over the years to find out how you are getting on. Ideally we would like to do this for life, but patients often change address and/or GP or lose touch with their hospital. If this happens we would like to use national records which are kept on everyone's health status to find out how you are. One of these is held at the General Register Office (GRO). We will need to give them enough information to identify you. This is usually your name, date of birth and NHS number (or Community Health Index and/or hospital number in Scotland). Any details we receive from any source are confidential and will only be used for the purposes of the HYBRID study. Please initial the consent form to show that we have your permission to do this – if you do not agree, we will not seek this information.

All the information that is sent to the ICR-CTSU will be kept until 20 years after the HYBRID study has ended.

Data sharing

The organisers of this study would like to be able to combine information we collect about patients in this study with information collected for other studies, if in the future it could advance our knowledge of the treatment of cancer. If this happens, information about you may be passed to other legitimate researchers, but they would not be able to identify you from the information provided.

What will happen to the results of the research study?

Independent experts will review the progress of the research, and the results will be published in a respected medical journal once we are sure they are reliable. No information that could identify you will be included and you will not be identified in any report or publication.

We will summarise the results for participants once they are available. Your hospital will be able to give you a copy and results will also be available on Cancer Research UK's patient website (www.cancerhelp.org.uk).

What if relevant new information becomes available?

Sometimes we get new information about the treatment being studied. If this happens, your doctor will tell you and discuss whether you should continue in the study. If you decide not to carry on, your doctor will make arrangements for your care to continue.

Will I be paid for taking part in this study?

No. Neither you nor your doctor will be paid for taking part in this study.

What if there is a problem?

Any complaint about the way you have been dealt with during this study, or any possible harm you might suffer, will be addressed. Your progress will be watched closely and you

Hafeez S, *et al. BMJ Open* 2020; 10:e037134. doi: 10.1136/bmjopen-2020-037134

(Form to be printed on headed paper)

will be offered whatever help is available to cope with any side effects. Occasionally some patients need a short stay in hospital for side effects to be treated, and on rare occasions these can be serious. If this were to happen, full details of what has happened will be reviewed carefully by the Doctor who has overall responsibility for the HYBRID trial. It is unlikely that anything will go wrong with your treatment or care, but if you wish to complain about any aspect of the way you have been approached or treated during the course of the study you can do so using the normal NHS complaints procedure. Concerns should be raised by speaking to a member of staff at your hospital or by talking to the local Patient Advice and Liaison Service (PALS) which has been established in every NHS Trust.

NHS bodies are liable for clinical negligence and other negligent harm to individuals covered by their duty of care. In the event that something does go wrong and you are harmed during the research and this is due to someone‘s negligence then you may have grounds for a legal action for compensation against the NHS Trust but you may have to pay your legal costs. Alternative indemnity arrangements apply to private clinics.

What if I don’t want to carry on with the study?
You are free to withdraw from the study at any time. You do not have to give a reason and your future treatment and care will not be affected. If you change your mind about having the treatment in this study, we would still like to collect information about how you are getting on. The information we need is routinely recorded in your medical records at your standard hospital visits and you would not need to do anything.

Who is organising and funding the research?
HYBRID is organised by leading doctors at the Royal Marsden Hospital in London and Sutton together with the Institute of Cancer Research in Sutton, Surrey. The research is approved and funded by Cancer Research UK. The National Health Service Research and Development Executive will pay for any extra nursing and administrative costs incurred by the hospitals.

Who reviewed this study?
All research in the NHS is looked at by an independent group of people, called a Research Ethics Committee, to protect participants’ safety, rights, wellbeing and dignity. HYBRID has been reviewed and approved by London Surrey Borders Ethics Committee on behalf of all hospitals throughout the UK. It has also been reviewed and approved by Cancer Research UK and
reviewed and endorsed by patient and carer representatives from the NCRI Consumer Liaison Group (www.ncri.org.uk).

What happens now?
You will have some time to think about the study and make your decision. Your doctor or nurse will be happy to answer any questions. You may wish to discuss it with your family or friends. Once you have reached your decision please let your doctor or nurse know. You will be asked to sign a consent form and will be given a copy to keep together with this information sheet. Please keep this information sheet and copies of the signed consent form. Your GP will be told that you are taking part in the HYBRID study. If at any time you have any questions about the study you should contact your hospital consultant.

Further information

Hafeez S, *et al. BMJ Open* 2020; 10:e037134. doi: 10.1136/bmjopen-2020-037134

(Form to be printed on headed paper)

Macmillan Cancer support is a registered charity and helps with all the things that people affected by cancer want and need, from specialist health care and information to practical, emotional and financial support (www.macmillan.org.uk). You can also learn more about clinical trials on the Cancer Research UK's patient website (www.cancerhelp.org.uk).

Contact details
If at any time you have any questions about the study you should contact your local study team:
Local consultant's name:
Local research nurse/radiographer:
Address:
Telephone:
24 hour contact number:

Thank you for your interest in our research.

Hafeez S, *et al. BMJ Open* 2020; 10:e037134. doi: 10.1136/bmjopen-2020-037134

(Form to be printed on headed paper)

MREC Number: 13/LO/1350
HYBRID trial ID:

CONSENT FORM

HYBRID:
A multicentre randomised phase II study of **Hy**pofractionated **B**ladder **R**adiotherapy with or without **I**mage guided a**d**aptive-predictive planning

Name of Researcher taking consent:

Please write your initials in the box to the right of each statement if you agree, and please sign at the bottom

1. I confirm that I have read and understand the patient information sheet version 1.1 dated 24 Sept 2013 for the above study. I have had the opportunity to consider the information, ask questions and have had these answered satisfactorily. ☐
2. I understand that my participation is voluntary and that I am free to withdraw at any time without giving any reason, without my medical care or legal rights being affected. ☐
3. If I withdraw from the study, I consent to my doctor providing authorised researchers with basic clinical information that would be routinely collected and written in my medical records. ☐
4. I understand that sections of any of my medical notes may be looked at by responsible individuals from the research team, from ethics committees, or from the NHS Trust, where it is relevant to my taking part in research. I give permission for these individuals to have access to my records. ☐
5. I consent to the Institute of Cancer Research using information held by the NHS and national databases to follow up my health status. ☐
6. Data sharing: I grant advance authorisation for the possible future sharing of information collected about me with other organisations, with the understanding that I will not be identifiable from this information *(optional)*. ☐
7. I agree to my GP being informed of my participation in the study. ☐
8. I agree to participate in the side effects questionnaire study. *(If the answer to this question is 'NO', you may still take part in HYBRID)* ☐
9. I agree to take part in HYBRID. ☐

Name of participant	Date	Signature
Name of person taking consent<br>(if different from researcher)	Date	Signature
Researcher (PI)	Date	Signature

1 copy for participant; 1 copy for Principal Investigator; 1 copy to be kept with hospital notes

HYBRID consent Version 1.1; 24 September 2013
