# Supplementary material for: Protocol for hypofractionated adaptive radiotherapy to the bladder within a multicentre phase II randomised trial: radiotherapy planning and delivery guidance
Source: BMJ Open. 2020 May 26;10(5):e037134. doi: 10.1136/bmjopen-2020-037134 (PMC7259864; doi:10.1136/bmjopen-2020-037134)
Supplement: Author's manuscript [file bmjopen-2020-037134.draft_revisions.pdf]

BMJ Open is committed to open peer review. As part of this commitment we make the peer review history of every article we publish publicly available.

When an article is published we post the peer reviewers' comments and the authors' responses online. We also post the versions of the paper that were used during peer review. These are the versions that the peer review comments apply to.

The versions of the paper that follow are the versions that were submitted during the peer review process. They are not the versions of record or the final published versions. They should not be cited or distributed as the published version of this manuscript.

BMJ Open is an open access journal and the full, final, typeset and author-corrected version of record of the manuscript is available on our site with no access controls, subscription charges or pay-per-view fees (http://bmjopen.bmj.com).

If you have any questions on BMJ Open's open peer review process please email info.bmjopen@bmj.com

BMJ Open

# Protocol for hypofractionated adaptive radiotherapy to the bladder within a multi-centre phase II randomised trial: radiotherapy planning and delivery guidance

| | |
|---|---|
| Journal: | *BMJ Open* |
| Manuscript ID | bmjopen-2020-037134 |
| Article Type: | Protocol |
| Date Submitted by the Author: | 20-Jan-2020 |
| Complete List of Authors: | Hafeez, Shaista; Institute of Cancer Research, Radiotherapy and Imaging ; Royal Marsden Hospital NHS Trust, Radiotherapy and Imaging<br>Patel, Emma; National Radiotherapy Trials Quality Assurance Group, Mount Vernon Hospital<br>Webster, Amanda; National Radiotherapy Trials Quality Assurance Group, Mount Vernon Hospital<br>Warren-oseni, Karole; Institute of Cancer Research, Radiotherapy and Imaging; Royal Marsden Hospital NHS Trust, Radiotherapy and Imaging<br>Hansen, Vibeke; Odense University Hospital, Laboratory of Radiation Physics<br>McNair, Helen; Institute of Cancer Research, Radiotherapy and Imaging ; Royal Marsden Hospital NHS Trust, Radiotherapy and Imaging<br>Miles, Elizabeth; National Radiotherapy Trials Quality Assurance Group, Mount Vernon Hospital<br>Lewis, Rebecca; Institute of Cancer Research, Clinical Trials and Statistics Unit<br>Hall, Emma; Institute of Cancer Research, Clinical Trials and Statistics Unit<br>Huddart, Robert; Institute of Cancer Research, Radiotherapy and Imaging; Royal Marsden Hospital NHS Trust, Radiotherapy and Imaging |
| Keywords: | RADIOTHERAPY, Radiation oncology < RADIOTHERAPY, Urological tumours < ONCOLOGY, Bladder disorders < UROLOGY |
| | |

SCHOLARONE™
Manuscripts

*I, the Submitting Author has the right to grant and does grant on behalf of all authors of the Work (as defined in the below author licence), an exclusive licence and/or a non-exclusive licence for contributions from authors who are: i) UK Crown employees; ii) where BMJ has agreed a CC-BY licence shall apply, and/or iii) in accordance with the terms applicable for US Federal Government officers or employees acting as part of their official duties; on a worldwide, perpetual, irrevocable, royalty-free basis to BMJ Publishing Group Ltd ("BMJ") its licensees and where the relevant Journal is co-owned by BMJ to the co-owners of the Journal, to publish the Work in this journal and any other BMJ products and to exploit all rights, as set out in our licence.*

*The Submitting Author accepts and understands that any supply made under these terms is made by BMJ to the Submitting Author unless you are acting as an employee on behalf of your employer or a postgraduate student of an affiliated institution which is paying any applicable article publishing charge ("APC") for Open Access articles. Where the Submitting Author wishes to make the Work available on an Open Access basis (and intends to pay the relevant APC), the terms of reuse of such Open Access shall be governed by a Creative Commons licence – details of these licences and which Creative Commons licence will apply to this Work are set out in our licence referred to above.*

*Other than as permitted in any relevant BMJ Author's Self Archiving Policies, I confirm this Work has not been accepted for publication elsewhere, is not being considered for publication elsewhere and does not duplicate material already published. I confirm all authors consent to publication of this Work and authorise the granting of this licence.*

For peer review only

# Protocol for hypofractionated adaptive radiotherapy to the bladder within a multi-centre phase II randomised trial: radiotherapy planning and delivery guidance

Hafeez S[1], Patel E[2], Webster A[2], Warren-Oseni K[1], Hansen V[3], McNair H[1], Miles E[2] Lewis R[4], Hall E[4], Huddart R[1]

[1]Department of Radiotherapy and Imaging, Institute of Cancer Research and The Royal Marsden NHS Foundation Trust, London, UK.

[2]National Radiotherapy Trials Quality Assurance Group, Mount Vernon Hospital, Northwood, United Kingdom

[3]Odense University Hospital, Laboratory of Radiation Physics, Denmark

[4]Clinical Trials and Statistics Unit, Institute of Cancer Research, London, UK.

**Author for correspondence**

Dr S Hafeez

The Royal Marsden NHS Foundation Trust

Downs Road

Sutton

Surrey

SM2 5PT

UK

shaista.hafeez@icr.ac.uk

For peer review only

## Abstract

### Introduction

Patients with muscle invasive bladder cancer (MIBC) who are unfit and unsuitable for standard radical treatment with cystectomy or daily radiotherapy present a large unmet clinical need. Untreated, they suffer high cancer specific mortality and risk significant disease related local symptoms. Hypofractionated radiotherapy (delivering higher doses in fewer fractions/visits) is a potential treatment solution but could be compromised by the mobile nature of the bladder, resulting in target misses in a significant proportion of fractions. Adaptive 'plan of the day' image guided radiotherapy delivery may improve the precision and accuracy of treatment. We aim to demonstrate within a randomised multi-centre phase II trial feasibility of 'plan of the day' hypofractionated bladder radiotherapy delivery with acceptable rates of toxicity.

### Methods and analysis

Patients with T2-T4aN0M0 MIBC receiving 36Gy in six weekly fractions are randomised (1:1) between treatment delivered using a single standard plan or adaptive radiotherapy using a library of three plans (small, medium, and large). A cone beam CT taken prior to each treatment is used to visualize the anatomy and select the most appropriate plan depending on the bladder shape and size. A comprehensive radiotherapy quality assurance (QA) programme has been instituted to ensure standardisation of radiotherapy planning and delivery. The primary endpoint is to exclude $\geq$30% acute grade $\geq$3 non-genitourinary toxicity at 3 months for adaptive radiotherapy in patients who received $\geq$1 fraction ($p0= 0.71$, $p1=0.9$, $\alpha= 0.05$, $\beta= 0.2$). Secondary endpoints include local disease control, symptom control, late toxicity, overall survival, patient reported outcomes, and proportion of fractions benefiting from adaptive planning. Target recruitment is 62 patients.

### Ethics and dissemination

The trial is approved by regional research ethics committee. The results will be disseminated via peer reviewed scientific journals, conference presentations, and submission to regulatory authorities.

### Registration details

The trial is registered at ClinicalTrials.gov (NCT01810757).


For peer review only - http://bmjopen.bmj.com/site/about/guidelines.xhtml

**Keywords**

muscle invasive bladder cancer, image guided adaptive radiotherapy, randomised control trial

For peer review only

**Article summary**

This article describes the first randomised control trial protocol evaluating an image guided adaptive radiotherapy technique. The study population is an elderly less fit group of patients with muscle invasive bladder cancer who are not suitable for radical treatment with cystectomy or daily radiotherapy. Patients will be planned to receive a total dose of 36Gy in six, weekly fractions randomised (1:1) between treatment delivered using either a single standard plan or adaptive radiotherapy using a library of plans (plan of the day). If successful, the trial will help facilitate implementation of a new advanced radiotherapy technique in the UK and inform tolerance of hypofractionated radiotherapy for a bladder cancer patient group with otherwise unmet clinical need.

**Strengths and limitations of this study**

Phase II national multi-centre non-comparative randomised control trial

Detailed guidance and training for novel radiotherapy technique provided to ensure standardisation

Pre-trial and on trial robust radiotherapy quality assurance programme

Primary endpoint focus is acute non-genitourinary grade 3 toxicity scoring



For peer review only - http://bmjopen.bmj.com/site/about/guidelines.xhtml

## Introduction

Standard radical management of muscle invasive bladder cancer (MIBC) involves either radical cystectomy or a course of daily radiotherapy delivered with radiosensitisation over 4-7 weeks [1-5]. Given the aetiological association of bladder cancer with smoking, cardiovascular and respiratory co-morbidities are common [6, 7]. Under treatment and poor access to effective treatment is particularly evident in older patient groups who have the highest risk of cancer related morbidity and death from initially curable bladder cancer [8].

Hypofractionated radiotherapy (delivering higher doses in fewer fractions/visits) may provide a potential treatment solution for these patients. The only multi-centre randomised control trial of hypofractionated bladder radiotherapy investigated two schedules of relatively low biological effectiveness; 35Gy in 10 fractions over 2 weeks and 21Gy in 3 fractions over 1 week [9]. Both treatment groups achieved similar symptom control with no significant difference in efficacy or toxicity evident between different radiotherapy schedules. Despite the palliative treatment intent, approximately 20% of patients achieved survival beyond 24 months [9]. Given the presumed dose response relationship of MIBC to radiotherapy, a higher biological effective dose would be expected to improve local disease and symptom control further [10].

A number of small single centre studies using the higher biological dose of 30-36Gy in 6 Gy per fraction suggest acceptable acute and late toxicity with local control achieved in over of 60% patients at 3 months [11-13]. Prospective multi-centre assessment of this radiotherapy schedule has not yet been performed.

Reliably targeting the bladder for radiotherapy is challenging. It is a relatively mobile structure subject to marked shape and volume change during a course of radiotherapy [14-16]. This has meant that historically bladder cancer radiotherapy has been delivered with some element of geographical miss (up to 57% of fractions) even when large safety margins of up to 1.5cm are applied to create the planning target volume (PTV) [17]. The expected consequence of dose intended for the target hitting adjacent normal structures is reduced tumour control and increased treatment related toxicity. Larger safety margins would more reliably encompass the bladder target variation but would further increase the normal tissue exposed to radiation dose, so increase side effects from treatment.

Volumetric soft tissue imaging made possible by cone beam CT (CBCT) technology integrated on current generation linear accelerators allows a 3D image to be acquired immediately prior to treatment. This informs positional adjustment to optimise target


For peer review only - http://bmjopen.bmj.com/site/about/guidelines.xhtml

coverage by the radiotherapy plan. It also has enabled 'plan of the day' solution. Rather than a single plan available for treatment, a library of plans can be created to cover the range of expected filling and positional variation of the bladder. Acquiring CBCT just prior to treatment allows visualisation of the soft tissue so that a plan which best covers the bladder target with least normal tissue irradiation can be selected for treatment that day [17].

In a single centre non-randomised phase II study we demonstrated feasibility of the 'plan of the day' approach using library of three plans in a MIBC patient population unfit for radical treatment [18]. Target coverage was maintained with reduction in dose to normal tissue irradiation compared to single standard plan [19]. The HYBRID trial seeks to examine whether this treatment approach can be consistently and safely delivered across multiple NHS centres.

Below, we describe the HYBRID trial protocol with particular emphasis on the radiotherapy procedural aspects, including preparatory imaging, treatment planning, delivery and evaluation, with the aim of providing comprehensive description of the radiotherapy implemented for the study.

**Hypothesis**

Adaptive radiotherapy techniques can be delivered at multiple centres and result in acceptable levels of acute non-genitourinary side effects experienced by patients with MIBC unsuitable for radical daily radiotherapy or cystectomy.

For peer review only

## Materials and analysis

### Study design

HYBRID is a non-blinded multicentre non-comparative randomised control phase II trial conducted in accordance with the Research Governance Framework for Health and Social Care and principles of Good Clinical Practice. The trial is sponsored by The Institute of Cancer Research, registered on the ClinicalTrials.gov database (NCT01810757) and is included in the National Institute for Health Research (NIHR) Clinical Research Network portfolio.

All patients are planned to receive a total dose of 36Gy in six weekly fractions randomised (1:1) between treatment delivered using a single standard plan (control) or adaptive radiotherapy using a library of plans. Randomisation takes place centrally by the trials unit (ICR-CTSU) within a maximum of 6 weeks prior to the planned radiotherapy start date.

The primary endpoint is to evaluate acute non-genitourinary grade 3 or greater toxicity as assessed using Common Terminology Criteria for Adverse Events (CTCAE v.4). The secondary end points are to assess local disease control at 3 months, control rate of presenting symptoms as measured by CTCAE v.4, patient reported outcomes as measured by IBDQ, KHQ, and EQ5D, late toxicity as measured by CTCAE v.4 and RTOG, time to local disease progression, overall survival, and proportion of fractions benefiting from adaptive planning.

The trial has a number of exploratory secondary endpoints related to the appropriate identification of plan selection, target coverage, and concordance between clinical and patient reported outcomes.

Figure 1 shows the trial schema and overview of follow-up. Table 1 provides summary of the scheduled pre-randomisation, on treatment, and post treatment assessments.

### Participants and eligibility

Target recruitment is 62 patients from fourteen participating UK centres. Patients with histological confirmation of invasive bladder cancer (T2-T4aN0M0) of any pathological sub-type unsuitable for radical cystectomy or radical daily radiotherapy for any reason including but not limited to performance status, co-morbidity, or patient refusal will be approached for inclusion. Eligible patients would have an expected


For peer review only - http://bmjopen.bmj.com/site/about/guidelines.xhtml

survival of greater than 6 months, be willing to accept assessment with cystoscopy following radiotherapy completion, and be able to attend for follow-up.

Patients with an indwelling urinary catheter, active or history of other malignancy within 2 years of randomisation except for non-melanomatous skin carcinoma, previous non-muscle invasive bladder tumours, and low risk prostate cancer (as defined by NCCN risk stratification as T1/T2a, Gleason 6 PSA <10) will be excluded. Those with previous history of radiation to the pelvis or other contra-indication to pelvic radiotherapy e.g. inflammatory bowel disease will also be excluded.

**Study treatment**

All participants should have a TURBT if possible prior to trial entry but this is not mandated, accepting that a proportion of patients will be unsuitable for this procedure. To permit sufficient time for radiotherapy planning, it is expected that treatment would commence within a maximum of 6 weeks from randomisation.

Participants will be planned to receive six, 6Gy fractions delivered weekly to a total dose of 36Gy. Those allocated to the standard planning group will have one radiotherapy plan generated which will be used to deliver all 6 treatments. A CBCT scan acquired just prior to treatment delivery can be used to inform an online position correction in accordance with National Radiotherapy Implementation Group Report, on Image Guided Radiotherapy (IGRT) [20] and standard local practice.

Participants allocated to adaptive planning will have three radiotherapy plans generated corresponding to a small, medium and large PTV. A CBCT taken immediately prior to each treatment delivery will be used to select the most appropriate 'plan of the day' depending on the bladder volume and shape. Plan selection is authorised to be carried out only by those radiographers or other practitioners (physicians or physicists) who have attained concordance with the gold standard PTV selection through the Radiotherapy Trials QA Group (RTTQA) IGRT credentialing. This is to ensure all those participating in plan selection have the necessary advanced skill level required for the study.

**Radiotherapy planning and delivery**

The radiotherapy planning and delivery guidance was developed in collaboration with the RTTQA group.

For peer review only

**Radiotherapy planning CT scan**

The patient preparation procedures are the same irrespective of randomisation arm. Patients are required to have an empty bladder for acquisition of the radiotherapy planning CT scan. Patients are therefore asked to void immediately before planning CT scan and not to drink fluids for 30 minutes before the planning scan. Given bladder deformation occurs with loaded rectum, patients are also encouraged to evacuate their bowels of flatus and faeces prior to scanning. The use of micro enemas is permitted if it is standard local practice but is not mandated.

Patients are positioned supine with arms comfortably positioned out of the radiotherapy field using appropriate immobilisation devices. CT slices of $\leq$3 mm thickness are obtained from at least 4cm above the dome of the bladder to 2cm below the ischial tuberosities. No oral or intravenous contrast is required.

**Target volume definition**

Volumes are defined according to the International Commission on Radiation Units and Measurements (ICRU) report 50, supplement report ICRU 62: Prescribing, Recording and Reporting Photon Beam Therapy and ICRU 83: Prescribing, Recording and Reporting Photon- Beam Intensity Modulated radiotherapy (IMRT) [23]. Consistent structure naming convention for target volumes and organs at risk is adopted for all patients participating within the trial.

Outlining should be carried out with the aid of all diagnostic MRI and CT scans wherever available. The clinical target volume (CTV) is contoured to encompass the gross tumour volume (GTV), the whole bladder, and any area of extravesical spread. The CTV includes 1.5cm of prostatic urethra in male patients or 1cm of urethra in female patients if tumour is at the base of bladder or if distant CIS is present. It is not required that the GTV is drawn as a separate structure.

The CTV will be expanded either isotropically by 1.5cm to create a single PTV for standard planning (control) or three PTVs using variable margins (small, medium, and large) for adaptive planning depending on the randomisation arm. The CTV to PTV expansion details have been derived from earlier phase I/II work [17-19] and are summarised in Table 2.


For peer review only - http://bmjopen.bmj.com/site/about/guidelines.xhtml

**Organs at risk delineation**

Organs at risk (OARs) are identified as the rectum, other bowel, and femoral heads. These structures are outlined as solid structures by defining their outer wall. The rectum is outlined to include the full circumference and rectal contents. The rectal outlining should extend from the lowest level of the ischial tuberosities to the rectosigmoid junction which identified as the level at which there is an anterior inflection of the bowel, best appreciated on sagittal reconstructions on the CT planning scan.

The small and large bowel (including sigmoid colon) is outlined as a single structure labelled 'other bowel'. Small and large bowel visible on relevant axial slices of the planning scan is outlined as individual loops. The cranial extent of 'other bowel' outlining should be 2cm beyond the superior extent of the standard PTV or large PTV as appropriate.

Both the femoral heads are outlined to the bottom of the femoral head curvature. The femoral necks not included.

**Radiotherapy planning**

Three-dimensional conformal radiotherapy (3DCRT) planning is recommended using three or four fields, however use of static 5-7-field intensity modulated radiotherapy (IMRT) or volumetric modulated arc radiotherapy (VMAT) treatment is permitted. It is accepted that the preferred treatment planning method may vary between participating centres but should be stated at the start of the trial and then be used for all patients enrolled there.

For patient's randomised to standard planning a single plan is created. For those patients randomised to adaptive planning a series of three plans are created using PTV small, PTV medium, and PTV large.

Three-dimensional dose distributions are produced for the overall prescribed dose of 36Gy in 6 fractions. The dose distribution is assessed for coverage of the PTV and normal tissues sparing using appropriate transverse sagittal and coronal views.

All plans are created to ensure that at least 98% of the PTV (PTV $D_{98\%}$) receives $\geq$90% (ideally $\geq$95%) of the prescribed dose, the median PTV dose (PTV $D_{50\%}$) is within 1% of the prescription dose, and the near-maximum (PTV $D_{2\%}$) is $\leq$107% (ideally $\leq$105%) of the prescribed dose. To minimise unexpected high dose outside the PTV, it is


For peer review only - http://bmjopen.bmj.com/site/about/guidelines.xhtml

required that 1cc of normal tissue outside the PTV should be ≤110% of the prescribed dose.

Dose to OARs should be as low as possible. To minimise dose to 'other bowel', it is recommended that the small plan for those randomised to adaptive radiotherapy aims to achieve the pre-defined optimal dose constraints, and the mandatory constraints for the medium plan. It is accepted that the rectum and bowel dose constraints of the large plan may not be met despite adequate optimisation. Assessment of 'other bowel' dose on the large plan represents an over estimation of true dose to 'other bowel' compared to when this plan is actually used to deliver treatment. This is because when the large plan is selected for treatment, a proportion of bowel moves out of the field with bladder filling. It is at the local principals' investigator discretion to accept the OARs doses.

The target volume and OAR dose volume constraints are summarised in Table 3 and Table 4 respectively.

**Pre-radiotherapy checks**

To minimise risk of error at the time of plan importing, exporting, and plan selection, it is recommended that each beam name and ID reflect the assigned plan. It is also important to ensure that the participating centre's local record and verify systems cannot mix beams from different plans at the time exporting from the treatment planning system and importing for treatment delivery. One way of achieving this is to create each plan with slightly different contributions from each field so that only the correct combination of beams can be chosen on any given day. Adding 2 points diagonally on the isocentre slice with a dose close to the 100% isodose would achieve this. All beams can then only be assigned from the same plan to each of the points as the reference point differs.

**Treatment delivery**

The same patient preparation instructions used at planning CT will be implemented prior to each fraction delivered.

CBCT of the pelvis should be acquired prior to each fraction irrespective of randomisation. For those patients randomised to standard (control) arm, pre-treatment CBCT should be used in accordance with guidance provided in the NRIG IGRT report



For peer review only - http://bmjopen.bmj.com/site/about/guidelines.xhtml

[20]. It is therefore expected that this CBCT will inform appropriate corrections (either manual or automatic) to be applied prior to the delivered fraction to ensure that treatment is accurately directed.

For those patients randomised to the adaptive (experimental) arm, the pre-treatment CBCT is acquired and registered to bone in accordance with the guidance provided in the NRIG IGRT report [20]. An appropriately trained radiographers or other practitioners reviews the bone matched CBCT assessing the bladder size and position in relation to the three PTVs and the coverage they provide. The PTV contour and corresponding plan providing the most suitable coverage with minimal normal tissue irradiation is selected. The most suitable contour is deemed to be that which encompasses the whole bladder CTV as seen on CBCT with an approximate 3mm margin to account for any intra-fraction filling that may occur during treatment delivery. A second appropriately trained radiographer or practitioner must confirm the selected PTV and corresponding plan. Once agreement has been reached any necessary couch correction is performed prior to treatment delivery with the selected plan.

If no PTV contour appears to provide suitable coverage of the bladder CTV, then it is advised that the patient is removed from the treatment couch and is asked to empty their bladder and, or bowel. The above steps are repeated with CBCT acquired just prior to treatment to reassess bladder. It advised that the centre contacts the RTTQA group for advice if the PTV still appears to provide inadequate target coverage.

**Treatment scheduling**

Treatment can be scheduled to start on any day of the week but each fraction should be delivered on the same day of the week at weekly intervals +/- 2 days. Therefore, a maximum interval of 9 days between fractions is acceptable in the event of machine breakdown or service. For any gaps longer than this, the participating centre is advised to contact the trial team.

**Radiotherapy protocol compliance programme**

A comprehensive radiotherapy QA programme led by the RTTQA group has been implemented for the HYBRID trial, and has been previously described [21, 22]. The QA programme aims to standardise contouring, planning, and delivery of image guided and adaptive bladder radiotherapy in participating centres. It comprises of both pre-trial and on-trial components including independent monitoring of appropriate treatment plan selection for the adaptive planning during patient recruitment.


For peer review only - http://bmjopen.bmj.com/site/about/guidelines.xhtml
For peer review only

Prior to trial entry participating centres are asked to complete an online facility questionnaire in order to gauge current local IGRT experience. A separate process document is used to collect task details of all aspects of a complete patient pathway.

The principal investigator (PI) at each participating centre is asked to contour two benchmark clinical cases as per protocol. Structured feedback is provided via RTTQA team to the PI.

All participating trial centres are required to complete a planning benchmark case. Centres are provided with access to CT DICOM data and pre-outlined structure set. They are requested to then plan this patient in their own treatment planning system as if randomised to the HYBRID adaptive arm. It is the responsibility of the local investigator to ensure that appropriate plan checking QA process is in place at their local institution. Once the three plans of the benchmark case have been created, reviewed, and accepted by the local PI, the DICOM CT, dose cubes, RTplan, and structure sets are returned in to the RTTQA group via secure file transfer and structured feedback is provided.

It is a pre-trial requirement that all participating centres have both an established IGRT training programme in place for their radiographers and be utilising CBCT to assess bladder treatment delivery. Trial specific bladder IGRT competency is completed through an on-line training package, practical workshop, and independent assessment of plan selection.

The online training consists of three practice cases each with 6 CBCTs to work through. Step by step instructions with correct plan selections is provided. Following this, a credentialing assessment consisting of 12 plan selections is carried out. The plan selections and matched reviews are assessed by the RTTQA group and structured feedback provided. Only those who meet minimum threshold of concordance of plan selection as pre-defined by the trial team will be approved for performing HYBRID plan selection.

As part of the on-trial QA, each participating centre visited by the RTTQA group during their first adaptive patient's treatment course for an on site review of the local image registration processes and plan selection decision-making. Once the first adaptive patient has been recruited from each participating centre, the plans, and plan


For peer review only - http://bmjopen.bmj.com/site/about/guidelines.xhtml

selections for treatment delivery will be retrospectively reviewed remotely prior to the second patient starting treatment.

All planning data and treatment delivery data (CBCT, registration objects and treatment forms) is collected and reviewed by the RTTQA group to ensure adherence to the HYBRID planning and delivery protocol is maintained. Remote retrospective plan selection review will take place for all adaptive radiotherapy patients during the trial.

**Statistical considerations**

The sample size is based on the primary endpoint of acute (up to 3 months after the end of radiotherapy) non-genitourinary CTCAE $\geq$grade 3 toxicity. Using data from the non-randomised phase II study it is expected that the acute non-genitourinary $\geq$grade 3 rate will be 10% in patients receiving adaptive planning [18]. The study is designed to rule out a 30% upper limit of $\geq$grade 3 non-genitourinary toxicity with each planning method. Using an A'Hern exact single stage design with 80% power and 5% alpha (one-sided) in each planning group, if 5 or more of 28 evaluable patients experience non-genitourinary $\geq$grade 3 toxicity then the acute toxicity associated with that planning technique will be assumed to be too high. To be evaluable for acute toxicity participants must receive at least 1 fraction of radiotherapy. Incorporating a 10% non-evaluable rate gives a target sample size of 62 patients (31 in each planning group).

The numbers and proportions of patients with acute non-genitourinary CTCAE v4 toxicity $\geq$grade 3 within the first 3 months of completing radiotherapy in each planning method will be presented together with 95% one-sided exact confidence intervals (the 90% two-sided confidence interval will also be presented).

Late toxicity will be summarised by frequencies and proportions at each time point by treatment group. Kaplan-Meier methods will be used to present time to event outcomes; due to small numbers no formal comparison is planned.


For peer review only - http://bmjopen.bmj.com/site/about/guidelines.xhtml

## Ethics and dissemination

The trial is approved by the London-Surrey Borders Research Ethics Committee (13/LO/1350).

## Safety reporting

Data is collected at each trial visit regarding any adverse events according to the CTCAE V4.0 grading system. The highest grade observed since the last visit should be reported. All serious adverse events (SAEs) are reported to the ICR-CTSU within 24 hours of the PI becoming aware of the event. SAEs should be followed up until clinical recovery is complete or until the condition has stabilised. Any safety concerns will be reported to the main REC by ICR-CTSU as part of the annual progress report.

## Trial monitoring and oversight

The trial is supervised by a Trial Management Group (TMG) that includes the Chief Investigator, trials unit scientific lead, statistician and co-ordinators along with co-investigators, identified collaborators including RTTQA group representative, and lay/consumer representative.

Oversight is provided by an independent Trials Steering Committee (TSC) and an independent data monitoring committee (IDMC).

There are no formal early stopping rules for efficacy or toxicity but, as per the statistical design, if 5 or more participants report non-genitourinary $\geq$grade 3 toxicities in one planning group then randomisation will cease. The IDMC would then review the data and advise on continuation of recruitment to the other planning method.

## Trial status and dissemination of results

The first patient was registered in April 2014. The study completed recruitment in August 2016. It is expected that the trial will report in 2020.

## Patient and public involvement

The HYBRID trial has been reviewed and endorsed by patient and carer representatives from the National Cancer Research Institute (NCRI) Consumer Liaison Group and the NCRI Clinical and Translational Radiotherapy Research Group (CTRAD) working group.


For peer review only - http://bmjopen.bmj.com/site/about/guidelines.xhtml

Patient and public involvement began at the protocol design and development stage via national and local consumer oversight committee review. This included the NIHR Biomedical Research Centre radiotherapy studies consumer panel at the Institute of Cancer Research and The Royal Marsden NHS Foundation Trust, and the National Cancer Research Institute (NCRI) Bladder Clinical Studies Group, which includes consumer representation.

Patients who had participated in the phase I study were asked to assess the burden of involvement required for participation in the HYBRID trial. This included review of the patient reported outcomes questionnaires.

The trial patient information sheet and consent form were reviewed by the South West London Cancer Research Network consumer group. Their feedback was adopted and incorporated in to the final version of both documents.

Patient representation on the Trial Management Group advises on day to day management of the trial including patient recruitment, and it is expected that they will also participate in dissemination of results via bladder cancer patient groups.

## Conclusions

HYBRID represents the first randomised trial of adaptive 'plan of the day' radiotherapy and provides a framework for implementation of this technique in the UK. We hope to demonstrate that this approach will result in satisfactory clinical outcomes for patients who are not suitable for radical treatment options. The aim would be to help demonstrate that adaptive hypofractionated radiotherapy is well tolerated and should be considered for a group of bladder cancer patients with otherwise significant unmet need.


For peer review only - http://bmjopen.bmj.com/site/about/guidelines.xhtml

**Figures**

Figure 1. Trial schema

**Tables**

Table 1. Schedule of assessments

Table 2. CTV to PTV expansion details

Table 3. Target volume constraints

Table 4. Organ at risk dose constraint guide

For peer review only

**Table 1. Schedule of assessments**

| Visit/Assessment | Pre-randomisation | Up to 14 days pre-treatment | On treatment (before each fraction) | 4 weeks after completion of radiotherapy | 3 months after completion of radiotherapy | 6 months after completion of radiotherapy | 12 months after completion of radiotherapy | 24 months after completion of radiotherapy | Annually thereafter |
|---|---|---|---|---|---|---|---|---|---|
| Histological confirmation of bladder cancer | X | | | | | | | | |
| Radiological assessment of bladder cancer (minimum CT abdomen and pelvis and chest x-ray) | X[1] | | | | | | | | |
| Acute toxicity assessment (CTCAE v.4) | | X | X | X | X | | | | |
| Full blood count, urea and electrolytes | | X | X[2] | | | | | | |
| Patient reported outcomes questionnaire (IBDQ, KHQ and EQ5D) | | X | X[3] | | X | X | | | |
| Cystoscopy under general anaesthetic with tumour bed biopsy *(if not possible, flexible cystoscopy with visual inspection of tumour bed and urine cytology)* | | | | | X | | | | |
| Late toxicity assessment (CTCAE v.4 and RTOG) | | | | | | X | X | X | |
| Flexible cystoscopy with visual inspection of tumour bed *(if not possible, urine cytology and pelvic CT scan)* | | | | | | X | X | | |
| Assessment of disease status | | | | | | | | X | X |

Footnotes
1. Baseline radiological assessment should take place ideally within 4 weeks and within a maximum of 6 weeks prior to randomisation
2. Full blood count, urea and electrolytes prior to fractions 2, 4 and 6 only
3. PRO questionnaire at fraction 6 only

For peer review only

**Table 2. CTV to PTV expansion details**

| Patient Randomisation | | CTV to PTV Expansion (cm) Laterally | Anteriorly | Posteriorly | Superiorly | Inferiorly |
|---|---|---|---|---|---|---|
| **Standard Plan** | PTV Standard | 1.5 | 1.5 | 1.5 | 1.5 | 1.5 |
| **Adaptive Plan** | PTV Small | 0.5 | 0.5 | 0.5 | 0.5 | 0.5 |
| | PTV Medium | 0.5 | 1.5 | 1.0 | 1.5 | 0.5 |
| | PTV Large | 0.8 | 2.0 | 1.2 | 2.5 | 0.8 |

For peer review only


For peer review only - http://bmjopen.bmj.com/site/about/guidelines.xhtml

**Table 3. Target volume constraints**

| Dose Constraints | Optimal | Mandatory |
|---|---|---|
| **PTV $D_{98\%}$** | ≥95% of prescribed dose | ≥90% of prescribed dose |
| **PTV $D_{50\%}$** | +/- 1% of prescribed dose | - |
| **PTV $D_{2\%}$** | ≤105% of prescribed dose | ≤107% of prescribed dose |
| **Normal Tissue $D_{1cc}$** | - | ≤110% of prescribed dose |

Where
PTV $D_{98\%}$ is the dose received by 98% of PTV
PTV $D_{50\%}$ is the dose received by 50% of PTV
PTV $D_{2\%}$ is the dose received by 2% of PTV
Normal Tissue $D_{1cc}$ is the dose received by 1cc of normal tissue outside the PTV

**Table 4. Organ at risk dose constraint guide**

| Organ at risk | *Constraint | | |
|---|---|---|---|
| | **Dose level** | **Optimal** | **Mandatory** |
| **Rectum** | 17Gy | 50% | 80% |
| | 28Gy | 20% | 60% |
| | 33Gy | 15% | 50% |
| | 36Gy | 5% | 30% |
| **Other bowel** | V25 | 139cc | 208cc |
| | V28 | 122cc | 183cc |
| | V31 | 105cc | 157cc |
| | V33 | 84cc | 126cc |
| | V36 | 26cc | 39cc |
| **Femoral heads** | 28Gy | - | 50% |

*The constraints provided serve only as a guide with recommendation that the optimal constraints particularly for other bowel should be met for the small plan and mandatory constraints should be met for medium plan.

For peer review only

## Contribution

All contributors meet at least of one the criteria recommended by the ICMJE. RH and EH conceived the study design. SH, HMcN, VH, RL, EH and RH were involvement in protocol development. SH wrote the first draft of the radiotherapy protocol and manuscript. SH, EP, AW, KWO, VH, HMcN, EM, RL, EH, and RH contributed to subsequent drafts and revisions of the radiotherapy protocol and manuscript.

## Competing interest

SH reports non-financial support from Elekta (Elekta AB, Stockholm, Sweden), non-financial support from Merck Sharp & Dohme (MSD), personal fees and non-financial support from Roche outside the submitted work; EP, AW, KWO, VH, HMcN, EM, and RL have no conflicts to disclose; EH reports grants from Cancer Research UK during the conduct of the study; grants from Accuray Inc., grants from Varian Medical Systems Inc., outside the submitted work; RH reports non-financial support from Janssen, grants and personal fees from MSD, personal fees from Bristol Myers Squibb, grants from CRUK, other from Nektar, personal fees and non-financial support from Roche, outside the submitted work.

## Funding

The HYBRID trial is funded by Cancer Research UK (CRUK/12/055). Cancer Research UK supports the work of the Clinical Trials and Statistics Unit at the Institute of Cancer Research (ICR-CTSU) through programme grant award (1491/A15955). The trial was supported by the National Radiotherapy Trials Quality Assurance Team (RTTQA).

## Acknowledgements

SH, KW, HMcN, RL, EH, and RH acknowledge this study represents independent research supported by the National Institute for Health Research (NIHR) Biomedical Research Centre at The Royal Marsden NHS Foundation Trust and the Institute of Cancer Research, London. The views expressed are those of the author(s) and not necessarily those of the NIHR or the Department of Health and Social Care.

## Data sharing


For peer review only - http://bmjopen.bmj.com/site/about/guidelines.xhtml

No additional data are available as submission relates to trial protocol only.

**References**


1. **Bladder cancer: diagnosis and management. NICE guidance 2.** . http://wwwniceorguk/guidance/ng2/evidence/full- *guideline-3744109* 2015.
2. James ND, Hussain SA, Hall E, Jenkins P, Tremlett J, Rawlings C, Crundwell M, Sizer B, Sreenivasan T, Hendron C *et al*: **Radiotherapy with or without chemotherapy in muscle-invasive bladder cancer**. *N Engl J Med* 2012, **366**(16):1477-1488.
3. Hoskin PJ, Rojas AM, Bentzen SM, Saunders MI: **Radiotherapy with concurrent carbogen and nicotinamide in bladder carcinoma**. *J Clin Oncol* 2010, **28**(33):4912-4918.
4. Bellmunt J, Orsola A, Leow JJ, Wiegel T, De Santis M, Horwich A: **Bladder cancer: ESMO Practice Guidelines for diagnosis, treatment and follow-up**. *Annals of Oncology* 2014, **25**(suppl 3):iii40-iii48.
5. Witjes JA, Lebret T, Comperat EM, Cowan NC, De Santis M, Bruins HM, Hernandez V, Espinos EL, Dunn J, Rouanne M *et al*: **Updated 2016 EAU Guidelines on Muscle-invasive and Metastatic Bladder Cancer**. *European Urology* 2017, **71**(3):462-475.
6. Freedman ND, Silverman DT, Hollenbeck AR, Schatzkin A, Abnet CC: **Association between smoking and risk of bladder cancer among men and women**. *JAMA* 2011, **306**(7):737-745.
7. Goossens-Laan CA, Leliveld AM, Verhoeven RH, Kil PJ, de Bock GH, Hulshof MC, de Jong IJ, Coebergh JW: **Effects of age and comorbidity on treatment and survival of patients with muscle-invasive bladder cancer**. *International journal of cancer Journal international du cancer* 2014, **135**(4):905-912.
8. Noon AP, Albertsen PC, Thomas F, Rosario DJ, Catto JW: **Competing mortality in patients diagnosed with bladder cancer: evidence of undertreatment in the elderly and female patients**. *Br J Cancer* 2013, **108**(7):1534-1540.
9. Duchesne GM, Bolger JJ, Griffiths GO, Trevor Roberts J, Graham JD, Hoskin PJ, Fossa SD, Uscinska BM, Parmar MK: **A randomized trial of hypofractionated schedules of palliative radiotherapy in the management of bladder carcinoma: results of medical research council trial BA09**. *Int J Radiat Oncol Biol Phys* 2000, **47**(2):379-388.
10. Pos FJ, Hart G, Schneider C, Sminia P: **Radical radiotherapy for invasive bladder cancer: What dose and fractionation schedule to choose?** *Int J Radiat Oncol Biol Phys* 2006, **64**(4):1168-1173.
11. Jose CC, Price A, Norman A, Jay G, Huddart R, Dearnaley DP, Horwich A: **Hypofractionated radiotherapy for patients with carcinoma of the bladder**. *Clin Oncol (R Coll Radiol)* 1999, **11**(5):330-333.
12. McLaren DB, Morrey D, Mason MD: **Hypofractionated radiotherapy for muscle invasive bladder cancer in the elderly**. *Radiother Oncol* 1997, **43**(2):171-174.
13. Rostom AY, Tahir S, Gershuny AR, Kandil A, Folkes A, White WF: **Once weekly irradiation for carcinoma of the bladder**. *Int J Radiat Oncol Biol Phys* 1996, **35**(2):289-292.
14. Fokdal L, Honore H, Hoyer M, Meldgaard P, Fode K, von der Maase H: **Impact of changes in bladder and rectal filling volume on organ motion and dose distribution of the bladder in radiotherapy for urinary bladder cancer**. *Int J Radiat Oncol Biol Phys* 2004, **59**(2):436-444.
15. Lotz HT, Pos FJ, Hulshof MC, van Herk M, Lebesque JV, Duppen JC, Remeijer P: **Tumor motion and deformation during external radiotherapy of bladder cancer**. *Int J Radiat Oncol Biol Phys* 2006, **64**(5):1551-1558.



For peer review only - http://bmjopen.bmj.com/site/about/guidelines.xhtml

16. Meijer GJ, Rasch C, Remeijer P, Lebesque JV: **Three-dimensional analysis of delineation errors, setup errors, and organ motion during radiotherapy of bladder cancer**. *Int J Radiat Oncol Biol Phys* 2003, **55**(5):1277-1287.
17. Lalondrelle S, Huddart R, Warren-Oseni K, Hansen VN, McNair H, Thomas K, Dearnaley D, Horwich A, Khoo V: **Adaptive-predictive organ localization using cone-beam computed tomography for improved accuracy in external beam radiotherapy for bladder cancer**. *Int J Radiat Oncol Biol Phys* 2011, **79**(3):705-712.
18. Hafeez S, McDonald F, Lalondrelle S, McNair H, Warren-Oseni K, Jones K, Harris V, Taylor H, Khoo V, Thomas K *et al*: **Clinical Outcomes of Image Guided Adaptive Hypofractionated Weekly Radiation Therapy for Bladder Cancer in Patients Unsuitable for Radical Treatment**. *Int J Radiat Oncol Biol Phys* 2017, **98**(1):115-122.
19. McDonald F, Lalondrelle S, Taylor H, Warren-Oseni K, Khoo V, McNair HA, Harris V, Hafeez S, Hansen VN, Thomas K *et al*: **Clinical implementation of adaptive hypofractionated bladder radiotherapy for improvement in normal tissue irradiation**. *Clin Oncol (R Coll Radiol)* 2013, **25**(9):549-556.
20. **National Radiotherapy Implementation Group Report Image Guided Radiotherapy (IGRT) Guidance for implementation and use**. *https://wwwsororg/sites/default/files/document-versions/National%20Radiotherapy%20Implementation%20Group%20Report%20IGRT%20Finalpdf* August 2012.
21. Tsang Y, Baker A, Patel E, Miles E: **A new era for clinical trial quality assurance: A credentialing programme for RTT led adaptive radiotherapy**. *Technical Innovations & Patient Support in Radiation Oncology* 2018, **5**:1-2.
22. Patel E, Tsang Y, Baker A, Callender J, Hafeez S, Hall E, Hansen VN, Lewis R, McNair H, Miles E *et al*: **Quality assuring "Plan of the day" selection in a multicentre adaptive bladder trial: Implementation of a pre-accrual IGRT guidance and assessment module**. *Clinical and Translational Radiation Oncology* 2019, **19**:27-32.
23. Measurements ICoRUa: **ICRU Report 83 Prescribing, Recording, and Reporting Intensity-Modulated Photon-Beam Therapy (IMRT)**. 2010.

For peer review only

**Figure 1. Trial schema**

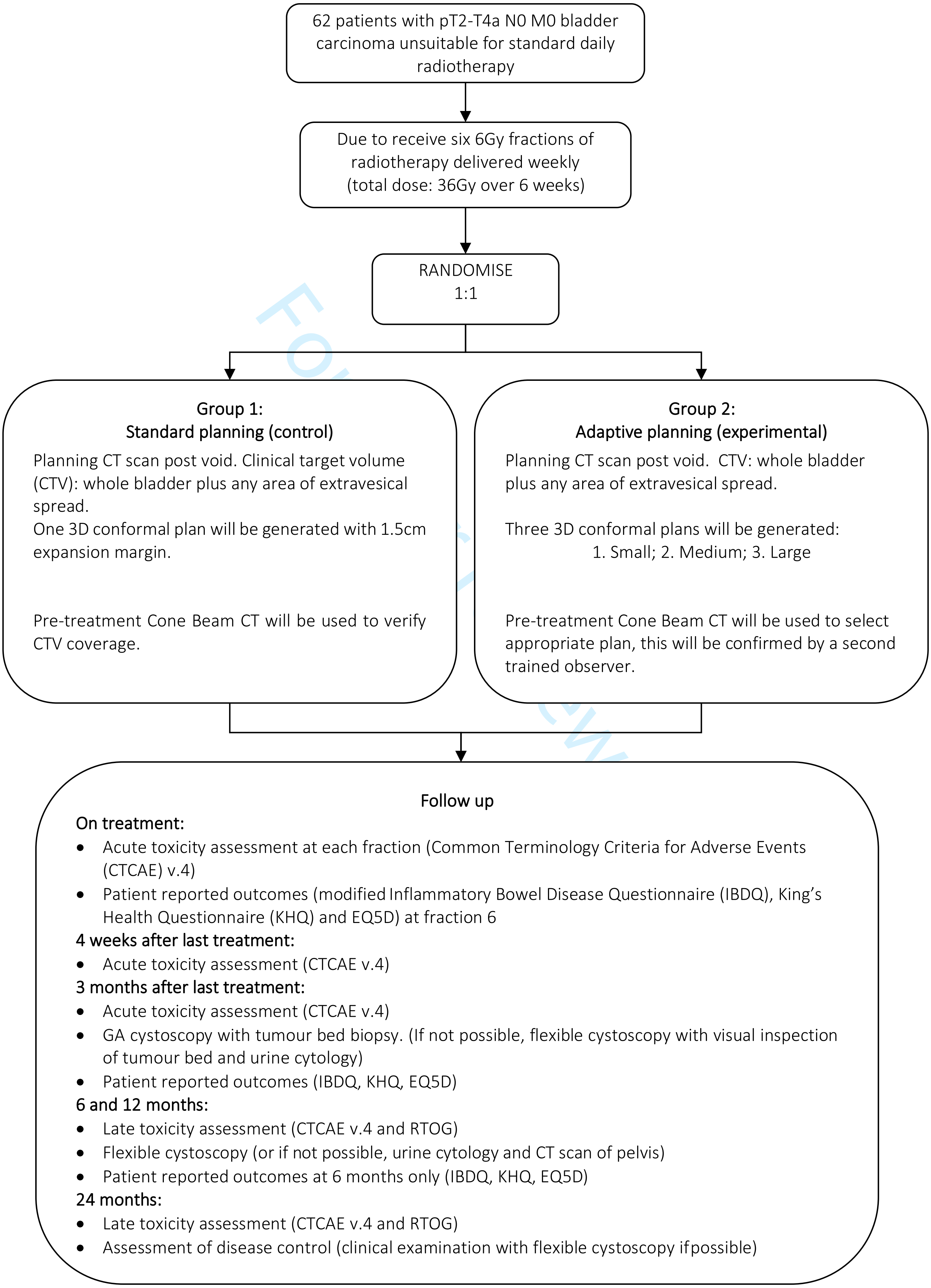

# Protocol for hypofractionated adaptive radiotherapy to the bladder within a multi-centre phase II randomised trial: radiotherapy planning and delivery guidance

| | |
|---|---|
| Journal: | *BMJ Open* |
| Manuscript ID | bmjopen-2020-037134.R1 |
| Article Type: | Protocol |
| Date Submitted by the Author: | 30-Mar-2020 |
| Complete List of Authors: | Hafeez, Shaista; Institute of Cancer Research, Radiotherapy and Imaging ; Royal Marsden Hospital NHS Trust, Radiotherapy and Imaging<br>Patel, Emma; National Radiotherapy Trials Quality Assurance Group, Mount Vernon Hospital<br>Webster, Amanda; National Radiotherapy Trials Quality Assurance Group, Mount Vernon Hospital<br>Warren-oseni, Karole; Institute of Cancer Research, Radiotherapy and Imaging; Royal Marsden Hospital NHS Trust, Radiotherapy and Imaging<br>Hansen, Vibeke; Odense University Hospital, Laboratory of Radiation Physics<br>McNair, Helen; Institute of Cancer Research, Radiotherapy and Imaging ; Royal Marsden Hospital NHS Trust, Radiotherapy and Imaging<br>Miles, Elizabeth; National Radiotherapy Trials Quality Assurance Group, Mount Vernon Hospital<br>Lewis, Rebecca; Institute of Cancer Research, Clinical Trials and Statistics Unit<br>Hall, Emma; Institute of Cancer Research, Clinical Trials and Statistics Unit<br>Huddart, Robert; Institute of Cancer Research, Radiotherapy and Imaging; Royal Marsden Hospital NHS Trust, Radiotherapy and Imaging |
| <b>Primary Subject Heading</b>: | Oncology |
| Secondary Subject Heading: | Urology |
| Keywords: | RADIOTHERAPY, Radiation oncology < RADIOTHERAPY, Urological tumours < ONCOLOGY, Bladder disorders < UROLOGY, ONCOLOGY |
| | |

*I, the Submitting Author has the right to grant and does grant on behalf of all authors of the Work (as defined in the below author licence), an exclusive licence and/or a non-exclusive licence for contributions from authors who are: i) UK Crown employees; ii) where BMJ has agreed a CC-BY licence shall apply, and/or iii) in accordance with the terms applicable for US Federal Government officers or employees acting as part of their official duties; on a worldwide, perpetual, irrevocable, royalty-free basis to BMJ Publishing Group Ltd ("BMJ") its licensees and where the relevant Journal is co-owned by BMJ to the co-owners of the Journal, to publish the Work in this journal and any other BMJ products and to exploit all rights, as set out in our licence.*

*The Submitting Author accepts and understands that any supply made under these terms is made by BMJ to the Submitting Author unless you are acting as an employee on behalf of your employer or a postgraduate student of an affiliated institution which is paying any applicable article publishing charge ("APC") for Open Access articles. Where the Submitting Author wishes to make the Work available on an Open Access basis (and intends to pay the relevant APC), the terms of reuse of such Open Access shall be governed by a Creative Commons licence – details of these licences and which Creative Commons licence will apply to this Work are set out in our licence referred to above.*

*Other than as permitted in any relevant BMJ Author's Self Archiving Policies, I confirm this Work has not been accepted for publication elsewhere, is not being considered for publication elsewhere and does not duplicate material already published. I confirm all authors consent to publication of this Work and authorise the granting of this licence.*

For peer review only

# Protocol for hypofractionated adaptive radiotherapy to the bladder within a multi-centre phase II randomised trial: radiotherapy planning and delivery guidance

Hafeez S[1], Patel E[2], Webster A[2], Warren-Oseni K[1], Hansen V[3], McNair H[1], Miles E[2] Lewis R[4], Hall E[4], Huddart R[1]

[1]Department of Radiotherapy and Imaging, Institute of Cancer Research and The Royal Marsden NHS Foundation Trust, London, UK.

[2]National Radiotherapy Trials Quality Assurance Group, Mount Vernon Hospital, Northwood, United Kingdom

[3]Odense University Hospital, Laboratory of Radiation Physics, Denmark

[4]Clinical Trials and Statistics Unit, Institute of Cancer Research, London, UK.

For peer review only

**Author for correspondence**

Dr S Hafeez

The Royal Marsden NHS Foundation Trust

Downs Road

Sutton

Surrey

SM2 5PT

UK

shaista.hafeez@icr.ac.uk



For peer review only - http://bmjopen.bmj.com/site/about/guidelines.xhtml

## Abstract

### Introduction

Patients with muscle invasive bladder cancer (MIBC) who are unfit and unsuitable for standard radical treatment with cystectomy or daily radiotherapy present a large unmet clinical need. Untreated, they suffer high cancer specific mortality and risk significant disease related local symptoms. Hypofractionated radiotherapy (delivering higher doses in fewer fractions/visits) is a potential treatment solution but could be compromised by the mobile nature of the bladder, resulting in target misses in a significant proportion of fractions. Adaptive 'plan of the day' image guided radiotherapy delivery may improve the precision and accuracy of treatment. We aim to demonstrate within a randomised multi-centre phase II trial feasibility of 'plan of the day' hypofractionated bladder radiotherapy delivery with acceptable rates of toxicity.

### Methods and analysis

Patients with T2-T4aN0M0 MIBC receiving 36Gy in six weekly fractions are randomised (1:1) between treatment delivered using a single standard plan or adaptive radiotherapy using a library of three plans (small, medium, and large). A cone beam CT taken prior to each treatment is used to visualize the anatomy and select the most appropriate plan depending on the bladder shape and size. A comprehensive radiotherapy quality assurance (QA) programme has been instituted to ensure standardisation of radiotherapy planning and delivery. The primary endpoint is to exclude $\geq$30% acute grade $\geq$3 non-genitourinary toxicity at 3 months for adaptive radiotherapy in patients who received $\geq$1 fraction ($p0=0.7$, $p1=0.9$, $\alpha= 0.05$, $\beta=0.2$). Secondary endpoints include local disease control, symptom control, late toxicity, overall survival, patient reported outcomes, and proportion of fractions benefiting from adaptive planning. Target recruitment is 62 patients.

### Ethics and dissemination

The trial is approved by the London-Surrey Borders Research Ethics Committee (13/LO/1350). The results will be disseminated via peer reviewed scientific journals, conference presentations, and submission to regulatory authorities.

### Registration details

The trial is registered at ClinicalTrials.gov (NCT01810757).




For peer review only - http://bmjopen.bmj.com/site/about/guidelines.xhtml

**Keywords**

muscle invasive bladder cancer, image guided adaptive radiotherapy, randomised control trial

For peer review only



For peer review only - http://bmjopen.bmj.com/site/about/guidelines.xhtml

**Strengths and limitations of this study**

- This is a phase II national multi-centre randomised control trial evaluating innovation in radiotherapy technology (strength).
- The trial has a non-comparative single stage design (limitation).
- Detailed guidance and training for this novel radiotherapy technique is provided to ensure standardisation across multiple participating centres (strength).
- A robust pre-trial and on trial radiotherapy quality assurance programme is in place to ensure standardisation of trial technique (strength).
- Primary endpoint focus is based on determining early effectiveness of this approach as measured by acute non-genitourinary grade 3 toxicity scoring (strength).

For peer review only

## Introduction

Standard radical management of muscle invasive bladder cancer (MIBC) involves either radical cystectomy or a course of daily radiotherapy delivered with radiosensitisation over 4-7 weeks [1-5]. Given the aetiological association of bladder cancer with smoking, cardiovascular and respiratory co-morbidities are common [6, 7]. Under treatment and poor access to effective treatment is particularly evident in older patient groups who have the highest risk of cancer related morbidity and death from initially curable bladder cancer [8].

Hypofractionated radiotherapy (delivering higher doses in fewer fractions/visits) may provide a potential treatment solution for these patients. The only multi-centre randomised control trial of hypofractionated bladder radiotherapy investigated two schedules of relatively low biological effectiveness; 35Gy in 10 fractions over 2 weeks and 21Gy in 3 fractions over 1 week [9]. Both treatment groups achieved similar symptom control with no significant difference in efficacy or toxicity evident between different radiotherapy schedules. Despite the palliative treatment intent, approximately 20% of patients achieved survival beyond 24 months [9]. Given the presumed dose response relationship of MIBC to radiotherapy, a higher biological effective dose would be expected to improve local disease and symptom control further [10].

A number of small single centre studies using the higher biological dose of 30-36Gy in 6 Gy per fraction suggest acceptable acute and late toxicity with local control achieved in over of 60% patients at 3 months [11-13]. Prospective multi-centre assessment of this radiotherapy schedule has not yet been performed.

Reliably targeting the bladder for radiotherapy is challenging. It is a relatively mobile structure subject to marked shape and volume change during a course of radiotherapy [14-16]. This has meant that historically bladder cancer radiotherapy has been delivered with some element of geographical miss (up to 57% of fractions) even when large safety margins of up to 1.5cm are applied to create the planning target volume (PTV) [17]. The expected consequence of dose intended for the target hitting adjacent normal structures is reduced tumour control and increased treatment related toxicity. Larger safety margins would more reliably encompass the bladder target variation but would further increase the normal tissue exposed to radiation dose, so increase side effects from treatment.


For peer review only - http://bmjopen.bmj.com/site/about/guidelines.xhtml

Volumetric soft tissue imaging made possible by cone beam CT (CBCT) technology integrated on current generation linear accelerators allows a 3D image to be acquired immediately prior to treatment. This informs positional adjustment to optimise target coverage by the radiotherapy plan. It also has enabled 'plan of the day' solution. Rather than a single plan available for treatment, a library of plans can be created to cover the range of expected filling and positional variation of the bladder. Acquiring CBCT just prior to treatment allows visualisation of the soft tissue so that a plan which best covers the bladder target with least normal tissue irradiation can be selected for treatment that day [17].

In a single centre non-randomised phase II study we demonstrated feasibility of the 'plan of the day' approach using library of three plans in a MIBC patient population unfit for radical treatment [18]. Target coverage was maintained with reduction in dose to normal tissue irradiation compared to single standard plan [19]. The HYBRID trial seeks to examine whether this treatment approach can be consistently and safely delivered across multiple NHS centres.

Below, we describe the HYBRID trial protocol with particular emphasis on the radiotherapy procedural aspects, including preparatory imaging, treatment planning, delivery and evaluation, with the aim of providing comprehensive description of the radiotherapy implemented for the study.

**Hypothesis**

Adaptive radiotherapy techniques can be delivered at multiple centres and result in acceptable levels of acute non-genitourinary side effects experienced by patients with MIBC unsuitable for radical daily radiotherapy or cystectomy.

For peer review only

## Materials and analysis

### Study design

HYBRID is a non-blinded multicentre non-comparative randomised control phase II trial conducted in accordance with the Research Governance Framework for Health and Social Care and principles of Good Clinical Practice. The trial is sponsored by The Institute of Cancer Research, registered on the ClinicalTrials.gov database (NCT01810757) and is included in the National Institute for Health Research (NIHR) Clinical Research Network portfolio.

All patients are planned to receive a total dose of 36Gy in six weekly fractions randomised (1:1) between treatment delivered using a single standard plan (control) or adaptive radiotherapy using a library of plans. Randomisation takes place centrally by the trials unit (ICR-CTSU) within a maximum of 6 weeks prior to the planned radiotherapy start date.

The primary endpoint is to evaluate acute non-genitourinary grade 3 or greater toxicity as assessed using Common Terminology Criteria for Adverse Events (CTCAE v.4). The secondary end points are to assess local disease control at 3 months, control rate of presenting symptoms as measured by CTCAE v.4, patient reported outcomes as measured by IBDQ, KHQ, and EQ5D, late toxicity as measured by CTCAE v.4 and RTOG, time to local disease progression, overall survival, and proportion of fractions benefiting from adaptive planning.

The trial has a number of exploratory secondary endpoints related to the appropriate identification of plan selection, target coverage, and concordance between clinical and patient reported outcomes.

Figure 1 shows the trial schema and overview of follow-up. Table 1 provides summary of the scheduled pre-randomisation, on treatment, and post treatment assessments.

### Participants and eligibility

Target recruitment is 62 patients from fourteen participating UK centres. Patients with histological confirmation of invasive bladder cancer (T2-T4aN0M0) of any pathological sub-type unsuitable for radical cystectomy or radical daily radiotherapy for any reason including but not limited to performance status, co-morbidity, or



For peer review only - http://bmjopen.bmj.com/site/about/guidelines.xhtml

For peer review only

patient refusal will be approached for inclusion. Eligible patients would have an expected survival of greater than 6 months, be willing to accept assessment with cystoscopy following radiotherapy completion, and be able to attend for follow-up.

Patients with an indwelling urinary catheter, active or history of other malignancy within 2 years of randomisation except for non-melanomatous skin carcinoma, previous non-muscle invasive bladder tumours, and low risk prostate cancer (as defined by NCCN risk stratification as T1/T2a, Gleason 6 PSA <10) will be excluded. Those with previous history of radiation to the pelvis or other contra-indication to pelvic radiotherapy e.g. inflammatory bowel disease will also be excluded.

**Study treatment**

All participants should have a TURBT if possible prior to trial entry but this is not mandated, accepting that a proportion of patients will be unsuitable for this procedure. To permit sufficient time for radiotherapy planning, it is expected that treatment would commence within a maximum of 6 weeks from randomisation.

Participants will be planned to receive six, 6Gy fractions delivered weekly to a total dose of 36Gy. Those allocated to the standard planning group will have one radiotherapy plan generated which will be used to deliver all 6 treatments. A CBCT scan acquired just prior to treatment delivery can be used to inform an online position correction in accordance with National Radiotherapy Implementation Group Report, on Image Guided Radiotherapy (IGRT) [20] and standard local practice.

Participants allocated to adaptive planning will have three radiotherapy plans generated corresponding to a small, medium and large PTV. A CBCT taken immediately prior to each treatment delivery will be used to select the most appropriate 'plan of the day' depending on the bladder volume and shape. Plan selection is authorised to be carried out only by those radiographers or other practitioners (physicians or physicists) who have attained concordance with the gold standard PTV selection through the Radiotherapy Trials QA Group (RTTQA) IGRT credentialing. This is to ensure all those participating in plan selection have the necessary advanced skill level required for the study.

**Radiotherapy planning and delivery**

The radiotherapy planning and delivery guidance was developed in collaboration with the RTTQA group.


For peer review only - http://bmjopen.bmj.com/site/about/guidelines.xhtml

**Radiotherapy planning CT scan**

The patient preparation procedures are the same irrespective of randomisation arm. Patients are required to have an empty bladder for acquisition of the radiotherapy planning CT scan. Patients are therefore asked to void immediately before planning CT scan and not to drink fluids for 30 minutes before the planning scan. Given bladder deformation occurs with loaded rectum, patients are also encouraged to evacuate their bowels of flatus and faeces prior to scanning. The use of micro enemas is permitted if it is standard local practice but is not mandated.

Patients are positioned supine with arms comfortably positioned out of the radiotherapy field using appropriate immobilisation devices. CT slices of ≤3 mm thickness are obtained from at least 4cm above the dome of the bladder to 2cm below the ischial tuberosities. No oral or intravenous contrast is required.

**Target volume definition**

Volumes are defined according to the International Commission on Radiation Units and Measurements (ICRU) report 50, supplement report ICRU 62: Prescribing, Recording and Reporting Photon Beam Therapy and ICRU 83: Prescribing, Recording and Reporting Photon- Beam Intensity Modulated radiotherapy (IMRT) [21]. Consistent structure naming convention for target volumes and organs at risk is adopted for all patients participating within the trial.

Outlining should be carried out with the aid of all diagnostic MRI and CT scans wherever available. The clinical target volume (CTV) is contoured to encompass the gross tumour volume (GTV), the whole bladder, and any area of extravesical spread. The CTV includes 1.5cm of prostatic urethra in male patients or 1cm of urethra in female patients if tumour is at the base of bladder or if distant CIS is present. It is not required that the GTV is drawn as a separate structure.

The CTV will be expanded either isotropically by 1.5cm to create a single PTV for standard planning (control) or three PTVs using variable margins (small, medium, and large) for adaptive planning depending on the randomisation arm. The CTV to PTV expansion details have been derived from earlier phase I/II work [17-19] and are summarised in Table 2.



For peer review only - http://bmjopen.bmj.com/site/about/guidelines.xhtml

**Organs at risk delineation**

Organs at risk (OARs) are identified as the rectum, other bowel, and femoral heads. These structures are outlined as solid structures by defining their outer wall. The rectum is outlined to include the full circumference and rectal contents. The rectal outlining should extend from the lowest level of the ischial tuberosities to the rectosigmoid junction which identified as the level at which there is an anterior inflection of the bowel, best appreciated on sagittal reconstructions on the CT planning scan.

The small and large bowel (including sigmoid colon) is outlined as a single structure labelled 'other bowel'. Small and large bowel visible on relevant axial slices of the planning scan is outlined as individual loops. The cranial extent of 'other bowel' outlining should be 2cm beyond the superior extent of the standard PTV or large PTV as appropriate.

Both the femoral heads are outlined to the bottom of the femoral head curvature. The femoral necks not included.

**Radiotherapy planning**

Three-dimensional conformal radiotherapy (3DCRT) planning is recommended using three or four fields, however use of static 5-7-field intensity modulated radiotherapy (IMRT) or volumetric modulated arc radiotherapy (VMAT) treatment is permitted. It is accepted that the preferred treatment planning method may vary between participating centres but should be stated at the start of the trial and then be used for all patients enrolled there.

For patient's randomised to standard planning a single plan is created. For those patients randomised to adaptive planning a series of three plans are created using PTV small, PTV medium, and PTV large.

Three-dimensional dose distributions are produced for the overall prescribed dose of 36Gy in 6 fractions. The dose distribution is assessed for coverage of the PTV and normal tissues sparing using appropriate transverse sagittal and coronal views.

All plans are created to ensure that at least 98% of the PTV (PTV $D_{98\%}$) receives $\geq$90% (ideally $\geq$95%) of the prescribed dose, the median PTV dose (PTV $D_{50\%}$) is


For peer review only - http://bmjopen.bmj.com/site/about/guidelines.xhtml

within 1% of the prescription dose, and the near-maximum (PTV $D_{2\%}$) is $\leq$107% (ideally $\leq$105%) of the prescribed dose. To minimise unexpected high dose outside the PTV, it is required that 1cc of normal tissue outside the PTV should be $\leq$110% of the prescribed dose.

Dose to OARs should be as low as possible. To minimise dose to 'other bowel', it is recommended that the small plan for those randomised to adaptive radiotherapy aims to achieve the pre-defined optimal dose constraints, and the mandatory constraints for the medium plan. It is accepted that the rectum and bowel dose constraints of the large plan may not be met despite adequate optimisation. Assessment of 'other bowel' dose on the large plan represents an over estimation of true dose to 'other bowel' compared to when this plan is actually used to deliver treatment. This is because when the large plan is selected for treatment, a proportion of bowel moves out of the field with bladder filling. It is at the local principals' investigator discretion to accept the OARs doses.

The target volume and OAR dose volume constraints are summarised in Table 3 and Table 4 respectively.

**Pre-radiotherapy checks**

To minimise risk of error at the time of plan importing, exporting, and plan selection, it is recommended that each beam name and ID reflect the assigned plan. It is also important to ensure that the participating centre's local record and verify systems cannot mix beams from different plans at the time exporting from the treatment planning system and importing for treatment delivery. One way of achieving this is to create each plan with slightly different contributions from each field so that only the correct combination of beams can be chosen on any given day. Adding 2 points diagonally on the isocentre slice with a dose close to the 100% isodose would achieve this. All beams can then only be assigned from the same plan to each of the points as the reference point differs.

**Treatment delivery**

The same patient preparation instructions used at planning CT will be implemented prior to each fraction delivered.



For peer review only - http://bmjopen.bmj.com/site/about/guidelines.xhtml

CBCT of the pelvis should be acquired prior to each fraction irrespective of randomisation. For those patients randomised to standard (control) arm, pre-treatment CBCT should be used in accordance with guidance provided in the NRIG IGRT report [20]. It is therefore expected that this CBCT will inform appropriate corrections (either manual or automatic) to be applied prior to the delivered fraction to ensure that treatment is accurately directed.

For those patients randomised to the adaptive (experimental) arm, the pre-treatment CBCT is acquired and registered to bone in accordance with the guidance provided in the NRIG IGRT report [20]. An appropriately trained radiographers or other practitioners reviews the bone matched CBCT assessing the bladder size and position in relation to the three PTVs and the coverage they provide. The PTV contour and corresponding plan providing the most suitable coverage with minimal normal tissue irradiation is selected. The most suitable contour is deemed to be that which encompasses the whole bladder CTV as seen on CBCT with an approximate 3mm margin to account for any intra-fraction filling that may occur during treatment delivery. A second appropriately trained radiographer or practitioner must confirm the selected PTV and corresponding plan. Once agreement has been reached any necessary couch correction is performed prior to treatment delivery with the selected plan.

If no PTV contour appears to provide suitable coverage of the bladder CTV, then it is advised that the patient is removed from the treatment couch and is asked to empty their bladder and, or bowel. The above steps are repeated with CBCT acquired just prior to treatment to reassess bladder. It advised that the centre contacts the RTTQA group for advice if the PTV still appears to provide inadequate target coverage.

**Treatment scheduling**

Treatment can be scheduled to start on any day of the week but each fraction should be delivered on the same day of the week at weekly intervals +/- 2 days. Therefore, a maximum interval of 9 days between fractions is acceptable in the event of machine breakdown or service. For any gaps longer than this, the participating centre is advised to contact the trial team.

**Radiotherapy protocol compliance programme**

A comprehensive radiotherapy QA programme led by the RTTQA group has been implemented for the HYBRID trial, and has been previously described [22, 23]. The


For peer review only - http://bmjopen.bmj.com/site/about/guidelines.xhtml

QA programme aims to standardise contouring, planning, and delivery of image guided and adaptive bladder radiotherapy in participating centres. It comprises of both pre-trial and on-trial components including independent monitoring of appropriate treatment plan selection for the adaptive planning during patient recruitment.

Prior to trial entry participating centres are asked to complete an online facility questionnaire in order to gauge current local IGRT experience.   A separate process document is used to collect task details of all aspects of a complete patient pathway.

The principal investigator (PI) at each participating centre is asked to contour two benchmark clinical cases as per protocol.  Structured feedback is provided via RTTQA team to the PI.

All participating trial centres are required to complete a planning benchmark case. Centres are provided with access to CT DICOM data and pre-outlined structure set. They are requested to then plan this patient in their own treatment planning system as if randomised to the HYBRID adaptive arm.  It is the responsibility of the local investigator to ensure that appropriate plan checking QA process is in place at their local institution.  Once the three plans of the benchmark case have been created, reviewed, and accepted by the local PI, the DICOM CT, dose cubes, RTplan, and structure sets are returned in to the RTTQA group via secure file transfer and structured feedback is provided.

It is a pre-trial requirement that all participating centres have both an established IGRT training programme in place for their radiographers and be utilising CBCT to assess bladder treatment delivery.   Trial specific bladder IGRT competency is completed through an on-line training package, practical workshop, and independent assessment of plan selection.

The online training consists of three practice cases each with 6 CBCTs to work through.   Step by step instructions with correct plan selections is provided. Following this, a credentialing assessment consisting of 12 plan selections is carried out. The plan selections and matched reviews are assessed by the RTTQA group and structured feedback provided.   Only those who meet minimum threshold of concordance of plan selection as pre-defined by the trial team will be approved for performing HYBRID plan selection.


For peer review only - http://bmjopen.bmj.com/site/about/guidelines.xhtml

As part of the on-trial QA, each participating centre visited by the RTTQA group during their first adaptive patient's treatment course for an on site review of the local image registration processes and plan selection decision-making. Once the first adaptive patient has been recruited from each participating centre, the plans, and plan selections for treatment delivery will be retrospectively reviewed remotely prior to the second patient starting treatment.

All planning data and treatment delivery data (CBCT, registration objects and treatment forms) is collected and reviewed by the RTTQA group to ensure adherence to the HYBRID planning and delivery protocol is maintained. Remote retrospective plan selection review will take place for all adaptive radiotherapy patients during the trial.

**Statistical considerations**

The primary objective is to assess whether adaptive radiotherapy techniques when delivered at multiple centres can lead to a reduction in the level of acute non-genitourinary (GU) toxicity experienced by patients with muscle invasive bladder cancer unsuitable for daily radical radiotherapy.

The sample size is based on the primary endpoint of acute (up to 3 months after the end of radiotherapy) non-genitourinary CTCAE $\geq$grade 3 toxicity. An A'Hern exact phase II design was used to rule out an upper limit for each planning method separately. Based on results of the APPLY study (NCT01000129) [18], it is expected that the acute non-genitourinary $\geq$grade 3 rate will be 10% ($p1=0.9$) in patients receiving adaptive planning. The study is designed to rule out a 30% ($p0=0.7$) upper limit of $\geq$grade 3 non-genitourinary toxicity with each planning method. For 80% power ($\beta=0.2$) and 5% alpha (one-sided) in each planning group, 28 evaluable patients are required and if 5 or more experience non-genitourinary $\geq$grade 3 toxicity then the acute toxicity associated with that planning technique will be assumed to be too high. To be evaluable for acute toxicity participants must receive at least 1 fraction of radiotherapy. Incorporating a 10% non-evaluable rate gives a target sample size of 62 patients (31 in each planning group).

The numbers and proportions of patients with acute non-genitourinary CTCAE v4 toxicity $\geq$grade 3 within the first 3 months of completing radiotherapy in each planning method will be presented together with 95% one-sided exact confidence intervals


For peer review only - http://bmjopen.bmj.com/site/about/guidelines.xhtml

(the 90% two-sided confidence interval will also be presented).

Late toxicity will be summarised by frequencies and proportions at each time point by treatment group. Kaplan-Meier methods will be used to present time to event outcomes; due to small numbers no formal comparison is planned.

**Ethics**

The trial is approved by the London-Surrey Borders Research Ethics Committee (13/LO/1350).

**Safety reporting**

Data is collected at each trial visit regarding any adverse events according to the CTCAE V4.0 grading system. The highest grade observed since the last visit should be reported. All serious adverse events (SAEs) are reported to the ICR-CTSU within 24 hours of the PI becoming aware of the event. SAEs should be followed up until clinical recovery is complete or until the condition has stabilised. Any safety concerns will be reported to the main Research and Ethics Comittee by ICR-CTSU as part of the annual progress report.

**Trial monitoring and oversight**

The trial is supervised by a Trial Management Group (TMG) that includes the Chief Investigator, trials unit scientific lead, statistician and co-ordinators along with co-investigators, identified collaborators including RTTQA group representative, and lay/consumer representative.

Oversight is provided by an independent Trials Steering Committee (TSC) and an independent data monitoring committee (IDMC).

There are no formal early stopping rules for efficacy or toxicity but, as per the statistical design, if 5 or more participants report non-genitourinary ≥grade 3 toxicities in one planning group then randomisation will cease. The IDMC would then review the data and advise on continuation of recruitment to the other planning method.

**Trial status and dissemination of results**

The first patient was registered in April 2014. The study completed recruitment in August 2016. It is expected that the trial will report in 2020. The results will be


For peer review only - http://bmjopen.bmj.com/site/about/guidelines.xhtml

disseminated via peer reviewed scientific journals, conference presentations, and submission to regulatory authorities.

**Patient and public involvement**

The HYBRID trial has been reviewed and endorsed by patient and carer representatives from the National Cancer Research Institute (NCRI) Consumer Liaison Group and the NCRI Clinical and Translational Radiotherapy Research Group (CTRAD) working group.

Patient and public involvement began at the protocol design and development stage via national and local consumer oversight committee review. This included the NIHR Biomedical Research Centre radiotherapy studies consumer panel at the Institute of Cancer Research and The Royal Marsden NHS Foundation Trust, and the National Cancer Research Institute (NCRI) Bladder Clinical Studies Group, which includes consumer representation.

Patients who had participated in the phase I study were asked to assess the burden of involvement required for participation in the HYBRID trial. This included review of the patient reported outcomes questionnaires.

The trial patient information sheet and consent form were reviewed by the South West London Cancer Research Network consumer group. Their feedback was adopted and incorporated in to the final version of both documents.

Patient representation on the Trial Management Group advises on day to day management of the trial including patient recruitment, and it is expected that they will also participate in dissemination of results via bladder cancer patient groups.


For peer review only - http://bmjopen.bmj.com/site/about/guidelines.xhtml

## Figures

Figure 1. Trial schema

## Tables

Table 1. Schedule of assessments

Table 2. CTV to PTV expansion details

Table 3. Target volume constraints

Table 4. Organ at risk dose constraint guide

## Supplementary information

Patient information sheet and consent form (Version 1.1; 24 September 2013)

For peer review only

**Table 1. Schedule of assessments**

| Visit/Assessment | Pre-randomisation | Up to 14 days pre-treatment | On treatment (before each fraction) | 4 weeks after completion of radiotherapy | 3 months after completion of radiotherapy | 6 months after completion of radiotherapy | 12 months after completion of radiotherapy | 24 months after completion of radiotherapy | Annually thereafter |
|---|---|---|---|---|---|---|---|---|---|
| Histological confirmation of bladder cancer | X | | | | | | | | |
| Radiological assessment of bladder cancer (minimum CT abdomen and pelvis and chest x-ray) | X[1] | | | | | | | | |
| Acute toxicity assessment (CTCAE v.4) | | X | X | X | X | | | | |
| Full blood count, urea and electrolytes | | X | X[2] | | | | | | |
| Patient reported outcomes questionnaire (IBDQ, KHQ and EQ5D) | | X | X[3] | | X | X | | | |
| Cystoscopy under general anaesthetic with tumour bed biopsy *(if not possible, flexible cystoscopy with visual inspection of tumour bed and urine cytology)* | | | | | X | | | | |
| Late toxicity assessment (CTCAE v.4 and RTOG) | | | | | | X | X | X | |
| Flexible cystoscopy with visual inspection of tumour bed *(if not possible, urine cytology and pelvic CT scan)* | | | | | | X | X | | |
| Assessment of disease status | | | | | | | | X | X |

Footnotes
1. Baseline radiological assessment should take place ideally within 4 weeks and within a maximum of 6 weeks prior to randomisation
2. Full blood count, urea and electrolytes prior to fractions 2, 4 and 6 only
3. PRO questionnaire at fraction 6 only

For peer review only

**Table 2. CTV to PTV expansion details**

| Patient Randomisation | | CTV to PTV Expansion (cm) Laterally | Anteriorly | Posteriorly | Superiorly | Inferiorly |
|---|---|---|---|---|---|---|
| **Standard Plan** | PTV Standard | 1.5 | 1.5 | 1.5 | 1.5 | 1.5 |
| **Adaptive Plan** | PTV Small | 0.5 | 0.5 | 0.5 | 0.5 | 0.5 |
| | PTV Medium | 0.5 | 1.5 | 1.0 | 1.5 | 0.5 |
| | PTV Large | 0.8 | 2.0 | 1.2 | 2.5 | 0.8 |

For peer review only

## Table 3. Target volume constraints

| Dose Constraints | Optimal | Mandatory |
|---|---|---|
| **PTV $D_{98\%}$** | ≥95% of prescribed dose | ≥90% of prescribed dose |
| **PTV $D_{50\%}$** | +/- 1% of prescribed dose | - |
| **PTV $D_{2\%}$** | ≤105% of prescribed dose | ≤107% of prescribed dose |
| **Normal Tissue $D_{1cc}$** | - | ≤110% of prescribed dose |

Where
PTV $D_{98\%}$ is the dose received by 98% of PTV
PTV $D_{50\%}$ is the dose received by 50% of PTV
PTV $D_{2\%}$ is the dose received by 2% of PTV
Normal Tissue $D_{1cc}$ is the dose received by 1cc of normal tissue outside the PTV

## Table 4. Organ at risk dose constraint guide

| Organ at risk | *Constraint | | |
|---|---|---|---|
| | Dose level | Optimal | Mandatory |
| **Rectum** | 17Gy | 50% | 80% |
| | 28Gy | 20% | 60% |
| | 33Gy | 15% | 50% |
| | 36Gy | 5% | 30% |
| **Other bowel** | V25 | 139cc | 208cc |
| | V28 | 122cc | 183cc |
| | V31 | 105cc | 157cc |
| | V33 | 84cc | 126cc |
| | V36 | 26cc | 39cc |
| **Femoral heads** | 28Gy | - | 50% |

*The constraints provided serve only as a guide with recommendation that the optimal constraints particularly for other bowel should be met for the small plan and mandatory constraints should be met for medium plan.

For peer review only

**Contribution**

All contributors meet at least of one the criteria recommended by the ICMJE. RH and EH conceived the study design. SH, HMcN, VH, RL, EH and RH were involved in protocol development. SH wrote the first draft of the radiotherapy protocol and manuscript. SH, EP, AW, KWO, VH, HMcN, EM, RL, EH, and RH contributed to subsequent drafts and revisions of the radiotherapy protocol and manuscript.

**Competing interest**

SH reports non-financial support from Elekta (Elekta AB, Stockholm, Sweden), non-financial support from Merck Sharp & Dohme (MSD), personal fees and non-financial support from Roche outside the submitted work; EP, AW, KWO, VH, HMcN, EM, and RL have no conflicts to disclose; EH reports grants from Cancer Research UK during the conduct of the study; grants from Accuray Inc., grants from Varian Medical Systems Inc., outside the submitted work; RH reports non-financial support from Janssen, grants and personal fees from MSD, personal fees from Bristol Myers Squibb, grants from CRUK, other from Nektar, personal fees and non-financial support from Roche, outside the submitted work.

**Funding**

The HYBRID trial is funded by Cancer Research UK (CRUK/12/055). Cancer Research UK supports the work of the Clinical Trials and Statistics Unit at the Institute of Cancer Research (ICR-CTSU) through a programme grant award (1491/A15955). The trial was supported by the National Radiotherapy Trials Quality Assurance Team (RTTQA).

**Acknowledgements**

SH, KW, HMcN, RL, EH, and RH acknowledge this study represents independent research supported by the National Institute for Health Research (NIHR) Biomedical Research Centre at The Royal Marsden NHS Foundation Trust and the Institute of Cancer Research, London. The views expressed are those of the author(s) and not necessarily those of the NIHR or the Department of Health and Social Care.

**Data sharing**

No additional data are available as submission relates to trial protocol only.

For peer review only

## References


1. **National Institute for Health and Clinical Excellence (NICE) Guidance. Bladder cancer: diagnosis and management (2015).** http://wwwniceorguk/guidance/ng2/evidence/full-guideline-3744109.
2. James ND, Hussain SA, Hall E, Jenkins P, Tremlett J, Rawlings C, Crundwell M, Sizer B, Sreenivasan T, Hendron C *et al*: **Radiotherapy with or without chemotherapy in muscle-invasive bladder cancer**. *N Engl J Med* 2012, **366**(16):1477-1488.
3. Hoskin PJ, Rojas AM, Bentzen SM, Saunders MI: **Radiotherapy with concurrent carbogen and nicotinamide in bladder carcinoma**. *J Clin Oncol* 2010, **28**(33):4912-4918.
4. Bellmunt J, Orsola A, Leow JJ, Wiegel T, De Santis M, Horwich A, Group EGW: **Bladder cancer: ESMO Practice Guidelines for diagnosis, treatment and follow-up**. *Ann Oncol* 2014, **25 Suppl 3**(suppl 3):iii40-48.
5. Witjes JA, Lebret T, Comperat EM, Cowan NC, De Santis M, Bruins HM, Hernandez V, Espinos EL, Dunn J, Rouanne M *et al*: **Updated 2016 EAU Guidelines on Muscle-invasive and Metastatic Bladder Cancer**. *European Urology* 2017, **71**(3):462-475.
6. Freedman ND, Silverman DT, Hollenbeck AR, Schatzkin A, Abnet CC: **Association between smoking and risk of bladder cancer among men and women**. *JAMA* 2011, **306**(7):737-745.
7. Goossens-Laan CA, Leliveld AM, Verhoeven RH, Kil PJ, de Bock GH, Hulshof MC, de Jong IJ, Coebergh JW: **Effects of age and comorbidity on treatment and survival of patients with muscle-invasive bladder cancer**. *International journal of cancer Journal international du cancer* 2014, **135**(4):905-912.
8. Noon AP, Albertsen PC, Thomas F, Rosario DJ, Catto JW: **Competing mortality in patients diagnosed with bladder cancer: evidence of undertreatment in the elderly and female patients**. *Br J Cancer* 2013, **108**(7):1534-1540.
9. Duchesne GM, Bolger JJ, Griffiths GO, Trevor Roberts J, Graham JD, Hoskin PJ, Fossa SD, Uscinska BM, Parmar MK: **A randomized trial of hypofractionated schedules of palliative radiotherapy in the management of bladder carcinoma: results of medical research council trial BA09**. *Int J Radiat Oncol Biol Phys* 2000, **47**(2):379-388.
10. Pos FJ, Hart G, Schneider C, Sminia P: **Radical radiotherapy for invasive bladder cancer: What dose and fractionation schedule to choose?** *Int J Radiat Oncol Biol Phys* 2006, **64**(4):1168-1173.
11. Jose CC, Price A, Norman A, Jay G, Huddart R, Dearnaley DP, Horwich A: **Hypofractionated radiotherapy for patients with carcinoma of the bladder**. *Clin Oncol (R Coll Radiol)* 1999, **11**(5):330-333.
12. McLaren DB, Morrey D, Mason MD: **Hypofractionated radiotherapy for muscle invasive bladder cancer in the elderly**. *Radiother Oncol* 1997, **43**(2):171-174.
13. Rostom AY, Tahir S, Gershuny AR, Kandil A, Folkes A, White WF: **Once weekly irradiation for carcinoma of the bladder**. *Int J Radiat Oncol Biol Phys* 1996, **35**(2):289-292.
14. Fokdal L, Honore H, Hoyer M, Meldgaard P, Fode K, von der Maase H: **Impact of changes in bladder and rectal filling volume on organ motion and dose distribution of the bladder in radiotherapy for urinary bladder cancer**. *Int J Radiat Oncol Biol Phys* 2004, **59**(2):436-444.
15. Lotz HT, Pos FJ, Hulshof MC, van Herk M, Lebesque JV, Duppen JC, Remeijer P: **Tumor motion and deformation during external radiotherapy of bladder cancer**. *Int J Radiat Oncol Biol Phys* 2006, **64**(5):1551-1558.
16. Meijer GJ, Rasch C, Remeijer P, Lebesque JV: **Three-dimensional analysis of delineation errors, setup errors, and organ motion during radiotherapy of bladder cancer**. *Int J Radiat Oncol Biol Phys* 2003, **55**(5):1277-1287.
17. Lalondrelle S, Huddart R, Warren-Oseni K, Hansen VN, McNair H, Thomas K, Dearnaley D, Horwich A, Khoo V: **Adaptive-predictive organ localization using cone-beam computed tomography for improved accuracy in external beam radiotherapy for bladder cancer**. *Int J Radiat Oncol Biol Phys* 2011, **79**(3):705-712.



For peer review only - http://bmjopen.bmj.com/site/about/guidelines.xhtml

18. Hafeez S, McDonald F, Lalondrelle S, McNair H, Warren-Oseni K, Jones K, Harris V, Taylor H, Khoo V, Thomas K *et al*: **Clinical Outcomes of Image Guided Adaptive Hypofractionated Weekly Radiation Therapy for Bladder Cancer in Patients Unsuitable for Radical Treatment**. *Int J Radiat Oncol Biol Phys* 2017, **98**(1):115-122.
19. McDonald F, Lalondrelle S, Taylor H, Warren-Oseni K, Khoo V, McNair HA, Harris V, Hafeez S, Hansen VN, Thomas K *et al*: **Clinical implementation of adaptive hypofractionated bladder radiotherapy for improvement in normal tissue irradiation**. *Clin Oncol (R Coll Radiol)* 2013, **25**(9):549-556.
20. **National Radiotherapy Implementation Group Report Image Guided Radiotherapy (IGRT) Guidance for implementation and use**. *https://wwwsororg/sites/default/files/document-versions/National%20Radiotherapy%20Implementation%20Group%20Report%20IGRT%20Finalpdf* August 2012.
21. Measurements ICoRUa: **ICRU Report 83 Prescribing, Recording, and Reporting Intensity-Modulated Photon-Beam Therapy (IMRT)**. 2010.
22. Tsang Y, Baker A, Patel E, Miles E: **A new era for clinical trial quality assurance: A credentialing programme for RTT led adaptive radiotherapy**. *Technical Innovations & Patient Support in Radiation Oncology* 2018, **5**:1-2.
23. Patel E, Tsang Y, Baker A, Callender J, Hafeez S, Hall E, Hansen VN, Lewis R, McNair H, Miles E *et al*: **Quality assuring "Plan of the day" selection in a multicentre adaptive bladder trial: Implementation of a pre-accrual IGRT guidance and assessment module**. *Clinical and Translational Radiation Oncology* 2019, **19**:27-32.

For peer review only


For peer review only - http://bmjopen.bmj.com/site/about/guidelines.xhtml

**Figure 1. Trial schema**

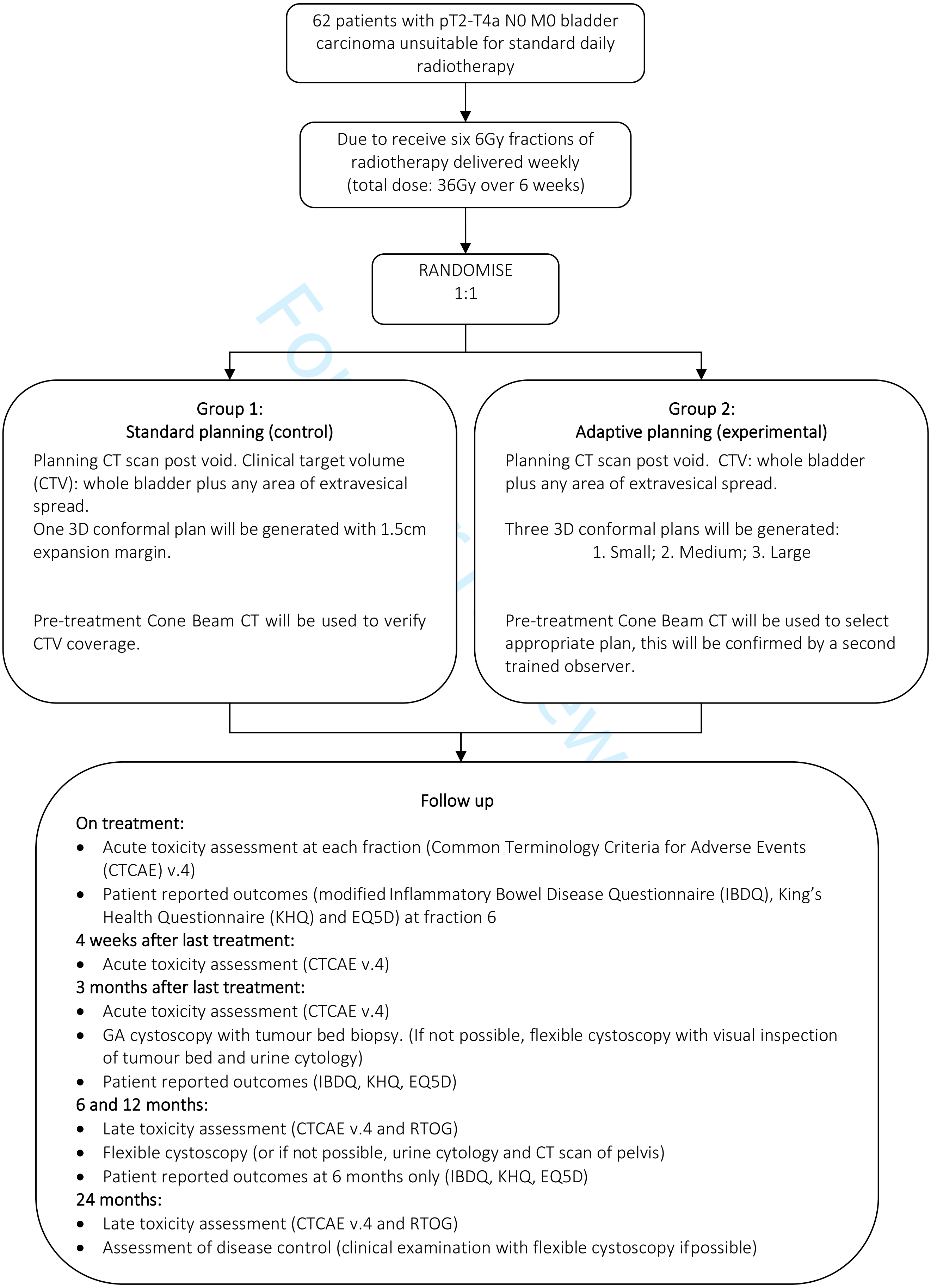


For peer review only - http://bmjopen.bmj.com/site/about/guidelines.xhtml

STANDARD PROTOCOL ITEMS: RECOMMENDATIONS FOR INTERVENTIONAL TRIALS

SPIRIT 2013 Checklist: Recommended items to address in a clinical trial protocol and related documents*

| Section/item | Item No | Description | Addressed on page number |
|---|---|---|---|
| **Administrative information** | | | |
| Title | 1 | Descriptive title identifying the study design, population, interventions, and, if applicable, trial acronym | p1 manuscript (p1 protocol) |
| Trial registration | 2a | Trial identifier and registry name. If not yet registered, name of intended registry | p7 manuscript (p1 protocol) |
| | 2b | All items from the World Health Organization Trial Registration Data Set | n/a |
| Protocol version | 3 | Date and version identifier | V 3.1 dated 13/8/2015 |
| Funding | 4 | Sources and types of financial, material, and other support | p21 manuscript (p21 protocol) |
| Roles and responsibilities | 5a | Names, affiliations, and roles of protocol contributors | p1 manuscript |
| | 5b | Name and contact information for the trial sponsor | p1 manuscript (p1 protocol) |
| | 5c | Role of study sponsor and funders, if any, in study design; collection, management, analysis, and interpretation of data; writing of the report; and the decision to submit the report for publication, including whether they will have ultimate authority over any of these activities | p26-28, 30 protocol |

For peer review only

| | | | |
|---|---|---|---|
| | 5d | Composition, roles, and responsibilities of the coordinating centre, steering committee, endpoint adjudication committee, data management team, and other individuals or groups overseeing the trial, if applicable (see Item 21a for data monitoring committee) | p15 manuscript (p27-29 protocol) |
| **Introduction** | | | |
| Background and rationale | 6a | Description of research question and justification for undertaking the trial, including summary of relevant studies (published and unpublished) examining benefits and harms for each intervention | p5-6 manuscript (p10-12 protocol) |
| | 6b | Explanation for choice of comparators | p11 protocol |
| Objectives | 7 | Specific objectives or hypotheses | p5-6 manuscript (p13 protocol) |
| Trial design | 8 | Description of trial design including type of trial (eg, parallel group, crossover, factorial, single group), allocation ratio, and framework (eg, superiority, equivalence, noninferiority, exploratory) | p7 manuscript (p13 protocol) |
| **Methods: Participants, interventions, and outcomes** | | | |
| Study setting | 9 | Description of study settings (eg, community clinic, academic hospital) and list of countries where data will be collected. Reference to where list of study sites can be obtained | p7 manuscript (p15 protocol) |
| Eligibility criteria | 10 | Inclusion and exclusion criteria for participants. If applicable, eligibility criteria for study centres and individuals who will perform the interventions (eg, surgeons, psychotherapists) | p7 manuscript (p15 protocol) |
| Interventions | 11a | Interventions for each group with sufficient detail to allow replication, including how and when they will be administered | p7-12 manuscript (p17-20 p15 protocol and additional radiotherapy planning and delivery protocol) |



For peer review only - http://bmjopen.bmj.com/site/about/guidelines.xhtml

| | | | |
|---|---|---|---|
| | 11b | Criteria for discontinuing or modifying allocated interventions for a given trial participant (eg, drug dose change in response to harms, participant request, or improving/worsening disease) | p15 manuscript (p27 protocol) |
| | 11c | Strategies to improve adherence to intervention protocols, and any procedures for monitoring adherence (eg, drug tablet return, laboratory tests) | p12-14 manuscript (p28-29 protocol and additional radiotherapy planning and delivery protocol) |
| | 11d | Relevant concomitant care and interventions that are permitted or prohibited during the trial | p27 protocol |
| Outcomes | 12 | Primary, secondary, and other outcomes, including the specific measurement variable (eg, systolic blood pressure), analysis metric (eg, change from baseline, final value, time to event), method of aggregation (eg, median, proportion), and time point for each outcome. Explanation of the clinical relevance of chosen efficacy and harm outcomes is strongly recommended | p7 manuscript (p14 protocol) |
| Participant timeline | 13 | Time schedule of enrolment, interventions (including any run-ins and washouts), assessments, and visits for participants. A schematic diagram is highly recommended (see Figure) | Table 1 manuscript (Table p19 of protocol) |
| Sample size | 14 | Estimated number of participants needed to achieve study objectives and how it was determined, including clinical and statistical assumptions supporting any sample size calculations | p14 manuscript (p24 protocol) |
| Recruitment | 15 | Strategies for achieving adequate participant enrolment to reach target sample size | p15-16 protocol |

**Methods: Assignment of interventions (for controlled trials)**

Allocation:

| | | | |
|---|---|---|---|
| Sequence generation | 16a | Method of generating the allocation sequence (eg, computer-generated random numbers), and list of any factors for stratification. To reduce predictability of a random sequence, details of any planned restriction (eg, blocking) should be provided in a separate document that is unavailable to those who enrol participants or assign interventions | p16, 25 protocol |



For peer review only - http://bmjopen.bmj.com/site/about/guidelines.xhtml

| | | | |
|---|---|---|---|
| Allocation concealment mechanism | 16b | Mechanism of implementing the allocation sequence (eg, central telephone; sequentially numbered, opaque, sealed envelopes), describing any steps to conceal the sequence until interventions are assigned | p16, 25 protocol |
| Implementation | 16c | Who will generate the allocation sequence, who will enrol participants, and who will assign participants to interventions | p16, 25 protocol |
| Blinding (masking) | 17a | Who will be blinded after assignment to interventions (eg, trial participants, care providers, outcome assessors, data analysts), and how | n/a |
| | 17b | If blinded, circumstances under which unblinding is permissible, and procedure for revealing a participant's allocated intervention during the trial | n/a |
| **Methods: Data collection, management, and analysis** | | | |
| Data collection methods | 18a | Plans for assessment and collection of outcome, baseline, and other trial data, including any related processes to promote data quality (eg, duplicate measurements, training of assessors) and a description of study instruments (eg, questionnaires, laboratory tests) along with their reliability and validity, if known. Reference to where data collection forms can be found, if not in the protocol | p28 protocol |
| | 18b | Plans to promote participant retention and complete follow-up, including list of any outcome data to be collected for participants who discontinue or deviate from intervention protocols | p18 protocol |
| Data management | 19 | Plans for data entry, coding, security, and storage, including any related processes to promote data quality (eg, double data entry; range checks for data values). Reference to where details of data management procedures can be found, if not in the protocol | p28 protocol |
| Statistical methods | 20a | Statistical methods for analysing primary and secondary outcomes. Reference to where other details of the statistical analysis plan can be found, if not in the protocol | P14 manuscript (p24, p35 protocol) |
| | 20b | Methods for any additional analyses (eg, subgroup and adjusted analyses) | P14 manuscript (p27 protocol) |
| | 20c | Definition of analysis population relating to protocol non-adherence (eg, as randomised analysis), and any statistical methods to handle missing data (eg, multiple imputation) | P14 manuscript (p28, p35 protocol) |



For peer review only - http://bmjopen.bmj.com/site/about/guidelines.xhtml

## Methods: Monitoring

| | | | |
|---|---|---|---|
| Data monitoring | 21a | Composition of data monitoring committee (DMC); summary of its role and reporting structure; statement of whether it is independent from the sponsor and competing interests; and reference to where further details about its charter can be found, if not in the protocol. Alternatively, an explanation of why a DMC is not needed | P15 manuscript (p27, p28 protocol) |
| | 21b | Description of any interim analyses and stopping guidelines, including who will have access to these interim results and make the final decision to terminate the trial | P15 manuscript (p27 protocol) |
| Harms | 22 | Plans for collecting, assessing, reporting, and managing solicited and spontaneously reported adverse events and other unintended effects of trial interventions or trial conduct | P15 manuscript (p21-24 protocol) |
| Auditing | 23 | Frequency and procedures for auditing trial conduct, if any, and whether the process will be independent from investigators and the sponsor | n/a |

## Ethics and dissemination

| | | | |
|---|---|---|---|
| Research ethics approval | 24 | Plans for seeking research ethics committee/institutional review board (REC/IRB) approval | P15 manuscript (P1, p29 protocol) |
| Protocol amendments | 25 | Plans for communicating important protocol modifications (eg, changes to eligibility criteria, outcomes, analyses) to relevant parties (eg, investigators, REC/IRBs, trial participants, trial registries, journals, regulators) | P15 manuscript (p2 protocol) |
| Consent or assent | 26a | Who will obtain informed consent or assent from potential trial participants or authorised surrogates, and how (see Item 32) | P16 protocol |
| | 26b | Additional consent provisions for collection and use of participant data and biological specimens in ancillary studies, if applicable | n/a |
| Confidentiality | 27 | How personal information about potential and enrolled participants will be collected, shared, and maintained in order to protect confidentiality before, during, and after the trial | p29-30 protocol |
| Declaration of interests | 28 | Financial and other competing interests for principal investigators for the overall trial and each study site | p21 manuscript |


For peer review only - http://bmjopen.bmj.com/site/about/guidelines.xhtml

| | | | |
|---|---|---|---|
| Access to data | 29 | Statement of who will have access to the final trial dataset, and disclosure of contractual agreements that limit such access for investigators | p21 manuscript (P30 protocol) |
| Ancillary and post-trial care | 30 | Provisions, if any, for ancillary and post-trial care, and for compensation to those who suffer harm from trial participation | P30 protocol |
| Dissemination policy | 31a | Plans for investigators and sponsor to communicate trial results to participants, healthcare professionals, the public, and other relevant groups (eg, via publication, reporting in results databases, or other data sharing arrangements), including any publication restrictions | P30 protocol |
| | 31b | Authorship eligibility guidelines and any intended use of professional writers | p21 manuscript (P30 protocol) |
| | 31c | Plans, if any, for granting public access to the full protocol, participant-level dataset, and statistical code | n/a |
| **Appendices** | | | |
| Informed consent materials | 32 | Model consent form and other related documentation given to participants and authorised surrogates | Included as supplementary |
| Biological specimens | 33 | Plans for collection, laboratory evaluation, and storage of biological specimens for genetic or molecular analysis in the current trial and for future use in ancillary studies, if applicable | n/a |

*It is strongly recommended that this checklist be read in conjunction with the SPIRIT 2013 Explanation & Elaboration for important clarification on the items. Amendments to the protocol should be tracked and dated. The SPIRIT checklist is copyrighted by the SPIRIT Group under the Creative Commons "Attribution-NonCommercial-NoDerivs 3.0 Unported" license.

For peer review only

BMJ Open

# Protocol for hypofractionated adaptive radiotherapy to the bladder within a multi-centre phase II randomised trial: radiotherapy planning and delivery guidance

| | |
|---|---|
| Journal: | *BMJ Open* |
| Manuscript ID | bmjopen-2020-037134.R2 |
| Article Type: | Protocol |
| Date Submitted by the Author: | 20-Apr-2020 |
| Complete List of Authors: | Hafeez, Shaista; Institute of Cancer Research, Radiotherapy and Imaging ; Royal Marsden Hospital NHS Trust, Radiotherapy and Imaging<br>Patel, Emma; National Radiotherapy Trials Quality Assurance Group, Mount Vernon Hospital<br>Webster, Amanda; National Radiotherapy Trials Quality Assurance Group, Mount Vernon Hospital<br>Warren-oseni, Karole; Institute of Cancer Research, Radiotherapy and Imaging; Royal Marsden Hospital NHS Trust, Radiotherapy and Imaging<br>Hansen, Vibeke; Odense University Hospital, Laboratory of Radiation Physics<br>McNair, Helen; Institute of Cancer Research, Radiotherapy and Imaging ; Royal Marsden Hospital NHS Trust, Radiotherapy and Imaging<br>Miles, Elizabeth; National Radiotherapy Trials Quality Assurance Group, Mount Vernon Hospital<br>Lewis, Rebecca; Institute of Cancer Research, Clinical Trials and Statistics Unit<br>Hall, Emma; Institute of Cancer Research, Clinical Trials and Statistics Unit<br>Huddart, Robert; Institute of Cancer Research, Radiotherapy and Imaging; Royal Marsden Hospital NHS Trust, Radiotherapy and Imaging |
| <b>Primary Subject Heading</b>: | Oncology |
| Secondary Subject Heading: | Urology |
| Keywords: | RADIOTHERAPY, Radiation oncology < RADIOTHERAPY, Urological tumours < ONCOLOGY, Bladder disorders < UROLOGY, ONCOLOGY |
| | |

SCHOLARONE™
Manuscripts

*I, the Submitting Author has the right to grant and does grant on behalf of all authors of the Work (as defined in the below author licence), an exclusive licence and/or a non-exclusive licence for contributions from authors who are: i) UK Crown employees; ii) where BMJ has agreed a CC-BY licence shall apply, and/or iii) in accordance with the terms applicable for US Federal Government officers or employees acting as part of their official duties; on a worldwide, perpetual, irrevocable, royalty-free basis to BMJ Publishing Group Ltd ("BMJ") its licensees and where the relevant Journal is co-owned by BMJ to the co-owners of the Journal, to publish the Work in this journal and any other BMJ products and to exploit all rights, as set out in our licence.*

*The Submitting Author accepts and understands that any supply made under these terms is made by BMJ to the Submitting Author unless you are acting as an employee on behalf of your employer or a postgraduate student of an affiliated institution which is paying any applicable article publishing charge ("APC") for Open Access articles. Where the Submitting Author wishes to make the Work available on an Open Access basis (and intends to pay the relevant APC), the terms of reuse of such Open Access shall be governed by a Creative Commons licence – details of these licences and which Creative Commons licence will apply to this Work are set out in our licence referred to above.*

*Other than as permitted in any relevant BMJ Author's Self Archiving Policies, I confirm this Work has not been accepted for publication elsewhere, is not being considered for publication elsewhere and does not duplicate material already published. I confirm all authors consent to publication of this Work and authorise the granting of this licence.*

For peer review only

# Protocol for hypofractionated adaptive radiotherapy to the bladder within a multi-centre phase II randomised trial: radiotherapy planning and delivery guidance

Hafeez S[1], Patel E[2], Webster A[2], Warren-Oseni K[1], Hansen V[3], McNair H[1], Miles E[2] Lewis R[4], Hall E[4], Huddart R[1]

[1]Department of Radiotherapy and Imaging, Institute of Cancer Research and The Royal Marsden NHS Foundation Trust, London, UK.

[2]National Radiotherapy Trials Quality Assurance Group, Mount Vernon Hospital, Northwood, United Kingdom

[3]Odense University Hospital, Laboratory of Radiation Physics, Denmark

[4]Clinical Trials and Statistics Unit, Institute of Cancer Research, London, UK.

**Author for correspondence**

Dr S Hafeez

The Royal Marsden NHS Foundation Trust

Downs Road

Sutton

Surrey

SM2 5PT

UK

shaista.hafeez@icr.ac.uk

For peer review only

## Abstract

### Introduction

Patients with muscle invasive bladder cancer (MIBC) who are unfit and unsuitable for standard radical treatment with cystectomy or daily radiotherapy present a large unmet clinical need. Untreated, they suffer high cancer specific mortality and risk significant disease related local symptoms. Hypofractionated radiotherapy (delivering higher doses in fewer fractions/visits) is a potential treatment solution but could be compromised by the mobile nature of the bladder, resulting in target misses in a significant proportion of fractions. Adaptive 'plan of the day' image guided radiotherapy delivery may improve the precision and accuracy of treatment. We aim to demonstrate within a randomised multi-centre phase II trial feasibility of 'plan of the day' hypofractionated bladder radiotherapy delivery with acceptable rates of toxicity.

### Methods and analysis

Patients with T2-T4aN0M0 MIBC receiving 36Gy in six weekly fractions are randomised (1:1) between treatment delivered using a single standard plan or adaptive radiotherapy using a library of three plans (small, medium, and large). A cone beam CT taken prior to each treatment is used to visualize the anatomy and select the most appropriate plan depending on the bladder shape and size. A comprehensive radiotherapy quality assurance (QA) programme has been instituted to ensure standardisation of radiotherapy planning and delivery. The primary endpoint is to exclude $\geq$30% acute grade $\geq$3 non-genitourinary toxicity at 3 months for adaptive radiotherapy in patients who received $\geq$1 fraction ($p0=0.7$, $p1=0.9$, $\alpha= 0.05$, $\beta=0.2$). Secondary endpoints include local disease control, symptom control, late toxicity, overall survival, patient reported outcomes, and proportion of fractions benefiting from adaptive planning. Target recruitment is 62 patients.

### Ethics and dissemination

The trial is approved by the London-Surrey Borders Research Ethics Committee (13/LO/1350). The results will be disseminated via peer reviewed scientific journals, conference presentations, and submission to regulatory authorities.

### Registration details

The trial is registered at ClinicalTrials.gov (NCT01810757).


For peer review only

**Keywords**

muscle invasive bladder cancer, image guided adaptive radiotherapy, randomised control trial

For peer review only


For peer review only - http://bmjopen.bmj.com/site/about/guidelines.xhtml

**Strengths and limitations of this study**

- This is a phase II national multi-centre randomised control trial evaluating innovation in radiotherapy technology (strength).
- The trial has a non-comparative single stage design (limitation).
- Detailed guidance and training for this novel radiotherapy technique is provided to ensure standardisation across multiple participating centres (strength).
- A robust pre-trial and on trial radiotherapy quality assurance programme is in place to ensure standardisation of trial technique (strength).
- Primary endpoint focus is based on determining early effectiveness of this approach as measured by acute non-genitourinary grade 3 toxicity scoring (strength).

For peer review only


For peer review only - http://bmjopen.bmj.com/site/about/guidelines.xhtml

## Introduction

Standard radical management of muscle invasive bladder cancer (MIBC) involves either radical cystectomy or a course of daily radiotherapy delivered with radiosensitisation over 4-7 weeks [1-5]. Given the aetiological association of bladder cancer with smoking, cardiovascular and respiratory co-morbidities are common [6, 7]. Under treatment and poor access to effective treatment is particularly evident in older patient groups who have the highest risk of cancer related morbidity and death from initially curable bladder cancer [8].

Hypofractionated radiotherapy (delivering higher doses in fewer fractions/visits) may provide a potential treatment solution for these patients. The only multi-centre randomised control trial of hypofractionated bladder radiotherapy investigated two schedules of relatively low biological effectiveness; 35Gy in 10 fractions over 2 weeks and 21Gy in 3 fractions over 1 week [9]. Both treatment groups achieved similar symptom control with no significant difference in efficacy or toxicity evident between different radiotherapy schedules. Despite the palliative treatment intent, approximately 20% of patients achieved survival beyond 24 months [9]. Given the presumed dose response relationship of MIBC to radiotherapy, a higher biological effective dose would be expected to improve local disease and symptom control further [10].

A number of small single centre studies using the higher biological dose of 30-36Gy in 6 Gy per fraction suggest acceptable acute and late toxicity with local control achieved in over of 60% patients at 3 months [11-13]. Prospective multi-centre assessment of this radiotherapy schedule has not yet been performed.

Reliably targeting the bladder for radiotherapy is challenging. It is a relatively mobile structure subject to marked shape and volume change during a course of radiotherapy [14-16]. This has meant that historically bladder cancer radiotherapy has been delivered with some element of geographical miss (up to 57% of fractions) even when large safety margins of up to 1.5cm are applied to create the planning target volume (PTV) [17]. The expected consequence of dose intended for the target hitting adjacent normal structures is reduced tumour control and increased treatment related toxicity. Larger safety margins would more reliably encompass the bladder target variation but would further increase the normal tissue exposed to radiation dose, so increase side effects from treatment.

Volumetric soft tissue imaging made possible by cone beam CT (CBCT) technology

For peer review only

integrated on current generation linear accelerators allows a 3D image to be acquired immediately prior to treatment. This informs positional adjustment to optimise target coverage by the radiotherapy plan. It also has enabled 'plan of the day' solution. Rather than a single plan available for treatment, a library of plans can be created to cover the range of expected filling and positional variation of the bladder. Acquiring CBCT just prior to treatment allows visualisation of the soft tissue so that a plan which best covers the bladder target with least normal tissue irradiation can be selected for treatment that day [17].

In a single centre non-randomised phase II study we demonstrated feasibility of the 'plan of the day' approach using library of three plans in a MIBC patient population unfit for radical treatment [18]. Target coverage was maintained with reduction in dose to normal tissue irradiation compared to single standard plan [19]. The HYBRID trial seeks to examine whether this treatment approach can be consistently and safely delivered across multiple NHS centres.

Below, we describe the HYBRID trial protocol with particular emphasis on the radiotherapy procedural aspects, including preparatory imaging, treatment planning, delivery and evaluation, with the aim of providing comprehensive description of the radiotherapy implemented for the study.

## Hypothesis

Adaptive radiotherapy techniques can be delivered at multiple centres and result in acceptable levels of acute non-genitourinary side effects experienced by patients with MIBC unsuitable for radical daily radiotherapy or cystectomy.


For peer review only - http://bmjopen.bmj.com/site/about/guidelines.xhtml

## Materials and analysis

### Study design

HYBRID is a non-blinded multicentre non-comparative randomised control phase II trial conducted in accordance with the Research Governance Framework for Health and Social Care and principles of Good Clinical Practice. The trial is sponsored by The Institute of Cancer Research, registered on the ClinicalTrials.gov database (NCT01810757) and is included in the National Institute for Health Research (NIHR) Clinical Research Network portfolio. The final ethics approved version of HYBRID trial protocol is provided in the supplementary files.

All patients are planned to receive a total dose of 36Gy in six weekly fractions randomised (1:1) between treatment delivered using a single standard plan (control) or adaptive radiotherapy using a library of plans. Randomisation takes place centrally by the trials unit (ICR-CTSU) within a maximum of 6 weeks prior to the planned radiotherapy start date.

The primary endpoint is to evaluate acute non-genitourinary grade 3 or greater toxicity as assessed using Common Terminology Criteria for Adverse Events (CTCAE v.4). The secondary end points are to assess local disease control at 3 months, control rate of presenting symptoms as measured by CTCAE v.4, patient reported outcomes as measured by IBDQ, KHQ, and EQ5D, late toxicity as measured by CTCAE v.4 and RTOG, time to local disease progression, overall survival, and proportion of fractions benefiting from adaptive planning.

The trial has a number of exploratory secondary endpoints related to the appropriate identification of plan selection, target coverage, and concordance between clinical and patient reported outcomes.

Figure 1 shows the trial schema and overview of follow-up. Table 1 provides summary of the scheduled pre-randomisation, on treatment, and post treatment assessments.

### Participants and eligibility

Target recruitment is 62 patients from fourteen participating UK centres. Patients with histological confirmation of invasive bladder cancer (T2-T4aN0M0) of any pathological sub-type unsuitable for radical cystectomy or radical daily radiotherapy for any reason including but not limited to performance status, co-morbidity, or patient



For peer review only - http://bmjopen.bmj.com/site/about/guidelines.xhtml

refusal will be approached for inclusion. Eligible patients would have an expected survival of greater than 6 months, be willing to accept assessment with cystoscopy following radiotherapy completion, and be able to attend for follow-up.

Patients with an indwelling urinary catheter, active or history of other malignancy within 2 years of randomisation except for non-melanomatous skin carcinoma, previous non-muscle invasive bladder tumours, and low risk prostate cancer (as defined by NCCN risk stratification as T1/T2a, Gleason 6 PSA <10) will be excluded. Those with previous history of radiation to the pelvis or other contra-indication to pelvic radiotherapy e.g. inflammatory bowel disease will also be excluded.

**Study treatment**

All participants should have a TURBT if possible prior to trial entry but this is not mandated, accepting that a proportion of patients will be unsuitable for this procedure. To permit sufficient time for radiotherapy planning, it is expected that treatment would commence within a maximum of 6 weeks from randomisation.

Participants will be planned to receive six, 6Gy fractions delivered weekly to a total dose of 36Gy. Those allocated to the standard planning group will have one radiotherapy plan generated which will be used to deliver all 6 treatments. A CBCT scan acquired just prior to treatment delivery can be used to inform an online position correction in accordance with National Radiotherapy Implementation Group Report, on Image Guided Radiotherapy (IGRT) [20] and standard local practice.

Participants allocated to adaptive planning will have three radiotherapy plans generated corresponding to a small, medium and large PTV. A CBCT taken immediately prior to each treatment delivery will be used to select the most appropriate 'plan of the day' depending on the bladder volume and shape. Plan selection is authorised to be carried out only by those radiographers or other practitioners (physicians or physicists) who have attained concordance with the gold standard PTV selection through the Radiotherapy Trials QA Group (RTTQA) IGRT credentialing. This is to ensure all those participating in plan selection have the necessary advanced skill level required for the study.

**Radiotherapy planning and delivery**



For peer review only - http://bmjopen.bmj.com/site/about/guidelines.xhtml

The radiotherapy planning and delivery guidance was developed in collaboration with the RTTQA group.

**Radiotherapy planning CT scan**

The patient preparation procedures are the same irrespective of randomisation arm. Patients are required to have an empty bladder for acquisition of the radiotherapy planning CT scan. Patients are therefore asked to void immediately before planning CT scan and not to drink fluids for 30 minutes before the planning scan. Given bladder deformation occurs with loaded rectum, patients are also encouraged to evacuate their bowels of flatus and faeces prior to scanning. The use of micro enemas is permitted if it is standard local practice but is not mandated.

Patients are positioned supine with arms comfortably positioned out of the radiotherapy field using appropriate immobilisation devices. CT slices of $\leq$3 mm thickness are obtained from at least 4cm above the dome of the bladder to 2cm below the ischial tuberosities. No oral or intravenous contrast is required.

**Target volume definition**

Volumes are defined according to the International Commission on Radiation Units and Measurements (ICRU) report 50, supplement report ICRU 62: Prescribing, Recording and Reporting Photon Beam Therapy and ICRU 83: Prescribing, Recording and Reporting Photon- Beam Intensity Modulated radiotherapy (IMRT) [21]. Consistent structure naming convention for target volumes and organs at risk is adopted for all patients participating within the trial.

Outlining should be carried out with the aid of all diagnostic MRI and CT scans wherever available. The clinical target volume (CTV) is contoured to encompass the gross tumour volume (GTV), the whole bladder, and any area of extravesical spread. The CTV includes 1.5cm of prostatic urethra in male patients or 1cm of urethra in female patients if tumour is at the base of bladder or if distant CIS is present. It is not required that the GTV is drawn as a separate structure.

The CTV will be expanded either isotropically by 1.5cm to create a single PTV for standard planning (control) or three PTVs using variable margins (small, medium, and large) for adaptive planning depending on the randomisation arm. The CTV to PTV expansion details have been derived from earlier phase I/II work [17-19] and are summarised in Table 2.



For peer review only - http://bmjopen.bmj.com/site/about/guidelines.xhtml

### Organs at risk delineation

Organs at risk (OARs) are identified as the rectum, other bowel, and femoral heads. These structures are outlined as solid structures by defining their outer wall. The rectum is outlined to include the full circumference and rectal contents. The rectal outlining should extend from the lowest level of the ischial tuberosities to the rectosigmoid junction which identified as the level at which there is an anterior inflection of the bowel, best appreciated on sagittal reconstructions on the CT planning scan.

The small and large bowel (including sigmoid colon) is outlined as a single structure labelled ‘other bowel’. Small and large bowel visible on relevant axial slices of the planning scan is outlined as individual loops. The cranial extent of ‘other bowel’ outlining should be 2cm beyond the superior extent of the standard PTV or large PTV as appropriate.

Both the femoral heads are outlined to the bottom of the femoral head curvature. The femoral necks not included.

### Radiotherapy planning

Three-dimensional conformal radiotherapy (3DCRT) planning is recommended using three or four fields, however use of static 5-7-field intensity modulated radiotherapy (IMRT) or volumetric modulated arc radiotherapy (VMAT) treatment is permitted. It is accepted that the preferred treatment planning method may vary between participating centres but should be stated at the start of the trial and then be used for all patients enrolled there.

For patient’s randomised to standard planning a single plan is created. For those patients randomised to adaptive planning a series of three plans are created using PTV small, PTV medium, and PTV large.

Three-dimensional dose distributions are produced for the overall prescribed dose of 36Gy in 6 fractions. The dose distribution is assessed for coverage of the PTV and normal tissues sparing using appropriate transverse sagittal and coronal views.

For peer review only

All plans are created to ensure that at least 98% of the PTV (PTV $D_{98\%}$) receives $\geq$90% (ideally $\geq$95%) of the prescribed dose, the median PTV dose (PTV $D_{50\%}$) is within 1% of the prescription dose, and the near-maximum (PTV $D_{2\%}$) is $\leq$107% (ideally $\leq$105%) of the prescribed dose. To minimise unexpected high dose outside the PTV, it is required that 1cc of normal tissue outside the PTV should be $\leq$110% of the prescribed dose.

Dose to OARs should be as low as possible. To minimise dose to 'other bowel', it is recommended that the small plan for those randomised to adaptive radiotherapy aims to achieve the pre-defined optimal dose constraints, and the mandatory constraints for the medium plan. It is accepted that the rectum and bowel dose constraints of the large plan may not be met despite adequate optimisation. Assessment of 'other bowel' dose on the large plan represents an over estimation of true dose to 'other bowel' compared to when this plan is actually used to deliver treatment. This is because when the large plan is selected for treatment, a proportion of bowel moves out of the field with bladder filling. It is at the local principals' investigator discretion to accept the OARs doses.

The target volume and OAR dose volume constraints are summarised in Table 3 and Table 4 respectively.

**Pre-radiotherapy checks**

To minimise risk of error at the time of plan importing, exporting, and plan selection, it is recommended that each beam name and ID reflect the assigned plan. It is also important to ensure that the participating centre's local record and verify systems cannot mix beams from different plans at the time exporting from the treatment planning system and importing for treatment delivery. One way of achieving this is to create each plan with slightly different contributions from each field so that only the correct combination of beams can be chosen on any given day. Adding 2 points diagonally on the isocentre slice with a dose close to the 100% isodose would achieve this. All beams can then only be assigned from the same plan to each of the points as the reference point differs.

**Treatment delivery**


For peer review only - http://bmjopen.bmj.com/site/about/guidelines.xhtml

The same patient preparation instructions used at planning CT will be implemented prior to each fraction delivered.

CBCT of the pelvis should be acquired prior to each fraction irrespective of randomisation. For those patients randomised to standard (control) arm, pre-treatment CBCT should be used in accordance with guidance provided in the NRIG IGRT report [20]. It is therefore expected that this CBCT will inform appropriate corrections (either manual or automatic) to be applied prior to the delivered fraction to ensure that treatment is accurately directed.

For those patients randomised to the adaptive (experimental) arm, the pre-treatment CBCT is acquired and registered to bone in accordance with the guidance provided in the NRIG IGRT report [20]. An appropriately trained radiographers or other practitioners reviews the bone matched CBCT assessing the bladder size and position in relation to the three PTVs and the coverage they provide. The PTV contour and corresponding plan providing the most suitable coverage with minimal normal tissue irradiation is selected. The most suitable contour is deemed to be that which encompasses the whole bladder CTV as seen on CBCT with an approximate 3mm margin to account for any intra-fraction filling that may occur during treatment delivery. A second appropriately trained radiographer or practitioner must confirm the selected PTV and corresponding plan. Once agreement has been reached any necessary couch correction is performed prior to treatment delivery with the selected plan.

If no PTV contour appears to provide suitable coverage of the bladder CTV, then it is advised that the patient is removed from the treatment couch and is asked to empty their bladder and, or bowel. The above steps are repeated with CBCT acquired just prior to treatment to reassess bladder. It advised that the centre contacts the RTTQA group for advice if the PTV still appears to provide inadequate target coverage.

**Treatment scheduling**

Treatment can be scheduled to start on any day of the week but each fraction should be delivered on the same day of the week at weekly intervals +/- 2 days. Therefore, a maximum interval of 9 days between fractions is acceptable in the event of machine breakdown or service. For any gaps longer than this, the participating centre is advised to contact the trial team.

**Radiotherapy protocol compliance programme**


For peer review only - http://bmjopen.bmj.com/site/about/guidelines.xhtml

A comprehensive radiotherapy QA programme led by the RTTQA group has been implemented for the HYBRID trial, and has been previously described [22, 23]. The QA programme aims to standardise contouring, planning, and delivery of image guided and adaptive bladder radiotherapy in participating centres. It comprises of both pre-trial and on-trial components including independent monitoring of appropriate treatment plan selection for the adaptive planning during patient recruitment.

Prior to trial entry participating centres are asked to complete an online facility questionnaire in order to gauge current local IGRT experience. A separate process document is used to collect task details of all aspects of a complete patient pathway.

The principal investigator (PI) at each participating centre is asked to contour two benchmark clinical cases as per protocol. Structured feedback is provided via RTTQA team to the PI.

All participating trial centres are required to complete a planning benchmark case. Centres are provided with access to CT DICOM data and pre-outlined structure set. They are requested to then plan this patient in their own treatment planning system as if randomised to the HYBRID adaptive arm. It is the responsibility of the local investigator to ensure that appropriate plan checking QA process is in place at their local institution. Once the three plans of the benchmark case have been created, reviewed, and accepted by the local PI, the DICOM CT, dose cubes, RTplan, and structure sets are returned in to the RTTQA group via secure file transfer and structured feedback is provided.

It is a pre-trial requirement that all participating centres have both an established IGRT training programme in place for their radiographers and be utilising CBCT to assess bladder treatment delivery. Trial specific bladder IGRT competency is completed through an on-line training package, practical workshop, and independent assessment of plan selection.

The online training consists of three practice cases each with 6 CBCTs to work through. Step by step instructions with correct plan selections is provided. Following this, a credentialing assessment consisting of 12 plan selections is carried out. The plan selections and matched reviews are assessed by the RTTQA group and structured feedback provided. Only those who meet minimum threshold of



For peer review only - http://bmjopen.bmj.com/site/about/guidelines.xhtml

concordance of plan selection as pre-defined by the trial team will be approved for performing HYBRID plan selection.

As part of the on-trial QA, each participating centre visited by the RTTQA group during their first adaptive patient's treatment course for an on site review of the local image registration processes and plan selection decision-making. Once the first adaptive patient has been recruited from each participating centre, the plans, and plan selections for treatment delivery will be retrospectively reviewed remotely prior to the second patient starting treatment.

All planning data and treatment delivery data (CBCT, registration objects and treatment forms) is collected and reviewed by the RTTQA group to ensure adherence to the HYBRID planning and delivery protocol is maintained. Remote retrospective plan selection review will take place for all adaptive radiotherapy patients during the trial.

**Statistical considerations**

The primary objective is to assess whether adaptive radiotherapy techniques when delivered at multiple centres can lead to a reduction in the level of acute non-genitourinary (GU) toxicity experienced by patients with muscle invasive bladder cancer unsuitable for daily radical radiotherapy.

The sample size is based on the primary endpoint of acute (up to 3 months after the end of radiotherapy) non-genitourinary CTCAE $\geq$grade 3 toxicity. An A'Hern exact phase II design was used to rule out an upper limit for each planning method separately. Based on results of the APPLY study (NCT01000129) [18], it is expected that the acute non-genitourinary $\geq$grade 3 rate will be 10% (p1=0.9) in patients receiving adaptive planning. The study is designed to rule out a 30% (p0=0.7) upper limit of $\geq$grade 3 non-genitourinary toxicity with each planning method. For 80% power ($\beta$=0.2) and 5% alpha (one-sided) in each planning group, 28 evaluable patients are required and if 5 or more experience non-genitourinary $\geq$grade 3 toxicity then the acute toxicity associated with that planning technique will be assumed to be too high. To be evaluable for acute toxicity participants must receive at least 1 fraction of radiotherapy. Incorporating a 10% non-evaluable rate gives a target sample size of 62 patients (31 in each planning group).

The numbers and proportions of patients with acute non-genitourinary CTCAE v4


For peer review only - http://bmjopen.bmj.com/site/about/guidelines.xhtml

toxicity ≥grade 3 within the first 3 months of completing radiotherapy in each planning method will be presented together with 95% one-sided exact confidence intervals (the 90% two-sided confidence interval will also be presented).

Late toxicity will be summarised by frequencies and proportions at each time point by treatment group. Kaplan-Meier methods will be used to present time to event outcomes; due to small numbers no formal comparison is planned.

**Ethics**

The trial is approved by the London-Surrey Borders Research Ethics Committee (13/LO/1350).

**Safety reporting**

Data is collected at each trial visit regarding any adverse events according to the CTCAE V4.0 grading system. The highest grade observed since the last visit should be reported. All serious adverse events (SAEs) are reported to the ICR-CTSU within 24 hours of the PI becoming aware of the event. SAEs should be followed up until clinical recovery is complete or until the condition has stabilised. Any safety concerns will be reported to the main Research and Ethics Committee by ICR-CTSU as part of the annual progress report.

**Trial monitoring and oversight**

The trial is supervised by a Trial Management Group (TMG) that includes the Chief Investigator, trials unit scientific lead, statistician and co-ordinators along with co-investigators, identified collaborators including RTTQA group representative, and lay/consumer representative.

Oversight is provided by an independent Trials Steering Committee (TSC) and an independent data monitoring committee (IDMC).

There are no formal early stopping rules for efficacy or toxicity but, as per the statistical design, if 5 or more participants report non-genitourinary ≥grade 3 toxicities in one planning group then randomisation will cease. The IDMC would then review the data and advise on continuation of recruitment to the other planning method.

**Trial status and dissemination of results**


For peer review only - http://bmjopen.bmj.com/site/about/guidelines.xhtml

The first patient was registered in April 2014. The study completed recruitment in August 2016. It is expected that the trial will report in 2020. The results will be disseminated via peer reviewed scientific journals, conference presentations, and submission to regulatory authorities.

**Patient and public involvement**

The HYBRID trial has been reviewed and endorsed by patient and carer representatives from the National Cancer Research Institute (NCRI) Consumer Liaison Group and the NCRI Clinical and Translational Radiotherapy Research Group (CTRAD) working group.

Patient and public involvement began at the protocol design and development stage via national and local consumer oversight committee review. This included the NIHR Biomedical Research Centre radiotherapy studies consumer panel at the Institute of Cancer Research and The Royal Marsden NHS Foundation Trust, and the National Cancer Research Institute (NCRI) Bladder Clinical Studies Group, which includes consumer representation.

Patients who had participated in the phase I study were asked to assess the burden of involvement required for participation in the HYBRID trial. This included review of the patient reported outcomes questionnaires.

The trial patient information sheet and consent form were reviewed by the South West London Cancer Research Network consumer group. Their feedback was adopted and incorporated in to the final version of both documents. Copy of the ethics approved final version of the patient information sheet and consent are provided in the supplementary files.

Patient representation on the Trial Management Group advises on day to day management of the trial including patient recruitment, and it is expected that they will also participate in dissemination of results via bladder cancer patient groups.

For peer review only


For peer review only - http://bmjopen.bmj.com/site/about/guidelines.xhtml

For peer review only


For peer review only - http://bmjopen.bmj.com/site/about/guidelines.xhtml

**Figures**

Figure 1. Trial schema

**Tables**

Table 1. Schedule of assessments

Table 2. CTV to PTV expansion details

Table 3. Target volume constraints

Table 4. Organ at risk dose constraint guide

**Supplementary information**

HYBRID patient information sheet and consent form (Version 1.1; 24 September 2013)

HYBRID trial protocol (Version 3.1; 13th August 2015)

For peer review only

## Table 1. Schedule of assessments

| Visit/Assessment | Pre-randomisation | Up to 14 days pre-treatment | On treatment (before each fraction) | 4 weeks after completion of radiotherapy | 3 months after completion of radiotherapy | 6 months after completion of radiotherapy | 12 months after completion of radiotherapy | 24 months after completion of radiotherapy | Annually thereafter |
|---|---|---|---|---|---|---|---|---|---|
| Histological confirmation of bladder cancer | X | | | | | | | | |
| Radiological assessment of bladder cancer (minimum CT abdomen and pelvis and chest x-ray) | X[1] | | | | | | | | |
| Acute toxicity assessment (CTCAE v.4) | | X | X | X | X | | | | |
| Full blood count, urea and electrolytes | | X | X[2] | | | | | | |
| Patient reported outcomes questionnaire (IBDQ, KHQ and EQ5D) | | X | X[3] | | X | X | | | |
| Cystoscopy under general anaesthetic with tumour bed biopsy *(if not possible, flexible cystoscopy with visual inspection of tumour bed and urine cytology)* | | | | | X | | | | |
| Late toxicity assessment (CTCAE v.4 and RTOG) | | | | | | X | X | X | |
| Flexible cystoscopy with visual inspection of tumour bed *(if not possible, urine cytology and pelvic CT scan)* | | | | | | X | X | | |
| Assessment of disease status | | | | | | | | X | X |

Footnotes
1. Baseline radiological assessment should take place ideally within 4 weeks and within a maximum of 6 weeks prior to randomisation
2. Full blood count, urea and electrolytes prior to fractions 2, 4 and 6 only
3. PRO questionnaire at fraction 6 only

For peer review only


For peer review only - http://bmjopen.bmj.com/site/about/guidelines.xhtml

**Table 2. CTV to PTV expansion details**

| Patient Randomisation | | CTV to PTV Expansion (cm) Laterally | Anteriorly | Posteriorly | Superiorly | Inferiorly |
|---|---|---|---|---|---|---|
| **Standard Plan** | PTV Standard | 1.5 | 1.5 | 1.5 | 1.5 | 1.5 |
| **Adaptive Plan** | PTV Small | 0.5 | 0.5 | 0.5 | 0.5 | 0.5 |
| | PTV Medium | 0.5 | 1.5 | 1.0 | 1.5 | 0.5 |
| | PTV Large | 0.8 | 2.0 | 1.2 | 2.5 | 0.8 |


For peer review only - http://bmjopen.bmj.com/site/about/guidelines.xhtml
For peer review only

**Table 3. Target volume constraints**

| Dose Constraints | Optimal | Mandatory |
|---|---|---|
| **PTV $D_{98\%}$** | ≥95% of prescribed dose | ≥90% of prescribed dose |
| **PTV $D_{50\%}$** | +/- 1% of prescribed dose | - |
| **PTV $D_{2\%}$** | ≤105% of prescribed dose | ≤107% of prescribed dose |
| **Normal Tissue $D_{1cc}$** | - | ≤110% of prescribed dose |

Where
PTV $D_{98\%}$ is the dose received by 98% of PTV
PTV $D_{50\%}$ is the dose received by 50% of PTV
PTV $D_{2\%}$ is the dose received by 2% of PTV
Normal Tissue $D_{1cc}$ is the dose received by 1cc of normal tissue outside the PTV

**Table 4. Organ at risk dose constraint guide**

| Organ at risk | *Constraint | | |
|---|---|---|---|
| | Dose level | Optimal | Mandatory |
| **Rectum** | 17Gy | 50% | 80% |
| | 28Gy | 20% | 60% |
| | 33Gy | 15% | 50% |
| | 36Gy | 5% | 30% |
| **Other bowel** | V25 | 139cc | 208cc |
| | V28 | 122cc | 183cc |
| | V31 | 105cc | 157cc |
| | V33 | 84cc | 126cc |
| | V36 | 26cc | 39cc |
| **Femoral heads** | 28Gy | - | 50% |

*The constraints provided serve only as a guide with recommendation that the optimal constraints particularly for other bowel should be met for the small plan and mandatory constraints should be met for medium plan.


For peer review only - http://bmjopen.bmj.com/site/about/guidelines.xhtml

### Contribution

All contributors meet at least of one the criteria recommended by the ICMJE. RH and EH conceived the study design. SH, HMcN, VH, RL, EH and RH were involved in protocol development. SH wrote the first draft of the radiotherapy protocol and manuscript. SH, EP, AW, KWO, VH, HMcN, EM, RL, EH, and RH contributed to subsequent drafts and revisions of the radiotherapy protocol and manuscript.

### Competing interest

SH reports non-financial support from Elekta (Elekta AB, Stockholm, Sweden), non-financial support from Merck Sharp & Dohme (MSD), personal fees and non-financial support from Roche outside the submitted work; EP, AW, KWO, VH, HMcN, EM, and RL have no conflicts to disclose; EH reports grants from Cancer Research UK during the conduct of the study; grants from Accuray Inc., grants from Varian Medical Systems Inc., outside the submitted work; RH reports non-financial support from Janssen, grants and personal fees from MSD, personal fees from Bristol Myers Squibb, grants from CRUK, other from Nektar, personal fees and non-financial support from Roche, outside the submitted work.

### Funding

The HYBRID trial is funded by Cancer Research UK (CRUK/12/055). Cancer Research UK supports the work of the Clinical Trials and Statistics Unit at the Institute of Cancer Research (ICR-CTSU) through a programme grant award (1491/A15955). The trial was supported by the National Radiotherapy Trials Quality Assurance Team (RTTQA).

### Acknowledgements

SH, KW, HMcN, RL, EH, and RH acknowledge this study represents independent research supported by the National Institute for Health Research (NIHR) Biomedical Research Centre at The Royal Marsden NHS Foundation Trust and the Institute of Cancer Research, London. The views expressed are those of the author(s) and not necessarily those of the NIHR or the Department of Health and Social Care.

### Data sharing

No additional data are available as submission relates to trial protocol only.

For peer review only

## References

1. **National Institute for Health and Clinical Excellence (NICE) Guidance. Bladder cancer: diagnosis and management (2015)**. http://wwwniceorguk/guidance/ng2/evidence/full-guideline-3744109.
2. James ND, Hussain SA, Hall E, Jenkins P, Tremlett J, Rawlings C, Crundwell M, Sizer B, Sreenivasan T, Hendron C *et al*: **Radiotherapy with or without chemotherapy in muscle-invasive bladder cancer**. *N Engl J Med* 2012, **366**(16):1477-1488.
3. Hoskin PJ, Rojas AM, Bentzen SM, Saunders MI: **Radiotherapy with concurrent carbogen and nicotinamide in bladder carcinoma**. *J Clin Oncol* 2010, **28**(33):4912-4918.
4. Bellmunt J, Orsola A, Leow JJ, Wiegel T, De Santis M, Horwich A, Group EGW: **Bladder cancer: ESMO Practice Guidelines for diagnosis, treatment and follow-up**. *Ann Oncol* 2014, **25 Suppl 3**(suppl 3):iii40-48.
5. Witjes JA, Lebret T, Comperat EM, Cowan NC, De Santis M, Bruins HM, Hernandez V, Espinos EL, Dunn J, Rouanne M *et al*: **Updated 2016 EAU Guidelines on Muscle-invasive and Metastatic Bladder Cancer**. *European Urology* 2017, **71**(3):462-475.
6. Freedman ND, Silverman DT, Hollenbeck AR, Schatzkin A, Abnet CC: **Association between smoking and risk of bladder cancer among men and women**. *JAMA* 2011, **306**(7):737-745.
7. Goossens-Laan CA, Leliveld AM, Verhoeven RH, Kil PJ, de Bock GH, Hulshof MC, de Jong IJ, Coebergh JW: **Effects of age and comorbidity on treatment and survival of patients with muscle-invasive bladder cancer**. *International journal of cancer Journal international du cancer* 2014, **135**(4):905-912.
8. Noon AP, Albertsen PC, Thomas F, Rosario DJ, Catto JW: **Competing mortality in patients diagnosed with bladder cancer: evidence of undertreatment in the elderly and female patients**. *Br J Cancer* 2013, **108**(7):1534-1540.
9. Duchesne GM, Bolger JJ, Griffiths GO, Trevor Roberts J, Graham JD, Hoskin PJ, Fossa SD, Uscinska BM, Parmar MK: **A randomized trial of hypofractionated schedules of palliative radiotherapy in the management of bladder carcinoma: results of medical research council trial BA09**. *Int J Radiat Oncol Biol Phys* 2000, **47**(2):379-388.
10. Pos FJ, Hart G, Schneider C, Sminia P: **Radical radiotherapy for invasive bladder cancer: What dose and fractionation schedule to choose?** *Int J Radiat Oncol Biol Phys* 2006, **64**(4):1168-1173.
11. Jose CC, Price A, Norman A, Jay G, Huddart R, Dearnaley DP, Horwich A: **Hypofractionated radiotherapy for patients with carcinoma of the bladder**. *Clin Oncol (R Coll Radiol)* 1999, **11**(5):330-333.
12. McLaren DB, Morrey D, Mason MD: **Hypofractionated radiotherapy for muscle invasive bladder cancer in the elderly**. *Radiother Oncol* 1997, **43**(2):171-174.
13. Rostom AY, Tahir S, Gershuny AR, Kandil A, Folkes A, White WF: **Once weekly irradiation for carcinoma of the bladder**. *Int J Radiat Oncol Biol Phys* 1996, **35**(2):289-292.
14. Fokdal L, Honore H, Hoyer M, Meldgaard P, Fode K, von der Maase H: **Impact of changes in bladder and rectal filling volume on organ motion and dose distribution of the bladder in radiotherapy for urinary bladder cancer**. *Int J Radiat Oncol Biol Phys* 2004, **59**(2):436-444.
15. Lotz HT, Pos FJ, Hulshof MC, van Herk M, Lebesque JV, Duppen JC, Remeijer P: **Tumor motion and deformation during external radiotherapy of bladder cancer**. *Int J Radiat Oncol Biol Phys* 2006, **64**(5):1551-1558.
16. Meijer GJ, Rasch C, Remeijer P, Lebesque JV: **Three-dimensional analysis of delineation errors, setup errors, and organ motion during radiotherapy of bladder cancer**. *Int J Radiat Oncol Biol Phys* 2003, **55**(5):1277-1287.
17. Lalondrelle S, Huddart R, Warren-Oseni K, Hansen VN, McNair H, Thomas K, Dearnaley D, Horwich A, Khoo V: **Adaptive-predictive organ localization using cone-beam computed tomography for improved accuracy in external beam radiotherapy for bladder cancer**. *Int J Radiat Oncol Biol Phys* 2011, **79**(3):705-712.
18. Hafeez S, McDonald F, Lalondrelle S, McNair H, Warren-Oseni K, Jones K, Harris V, Taylor H, Khoo V, Thomas K *et al*: **Clinical Outcomes of Image Guided Adaptive**


For peer review only - http://bmjopen.bmj.com/site/about/guidelines.xhtml

**Hypofractionated Weekly Radiation Therapy for Bladder Cancer in Patients Unsuitable for Radical Treatment**. *Int J Radiat Oncol Biol Phys* 2017, **98**(1):115-122.

19. McDonald F, Lalondrelle S, Taylor H, Warren-Oseni K, Khoo V, McNair HA, Harris V, Hafeez S, Hansen VN, Thomas K *et al*: **Clinical implementation of adaptive hypofractionated bladder radiotherapy for improvement in normal tissue irradiation**. *Clin Oncol (R Coll Radiol)* 2013, **25**(9):549-556.
20. **National Radiotherapy Implementation Group Report Image Guided Radiotherapy (IGRT) Guidance for implementation and use**. *https://wwwsororg/sites/default/files/document-versions/National%20Radiotherapy%20Implementation%20Group%20Report%20IGRT%20Finalpdf* August 2012.
21. Measurements ICoRUa: **ICRU Report 83 Prescribing, Recording, and Reporting Intensity-Modulated Photon-Beam Therapy (IMRT)**. 2010.
22. Tsang Y, Baker A, Patel E, Miles E: **A new era for clinical trial quality assurance: A credentialing programme for RTT led adaptive radiotherapy**. *Technical Innovations & Patient Support in Radiation Oncology* 2018, **5**:1-2.
23. Patel E, Tsang Y, Baker A, Callender J, Hafeez S, Hall E, Hansen VN, Lewis R, McNair H, Miles E *et al*: **Quality assuring "Plan of the day" selection in a multicentre adaptive bladder trial: Implementation of a pre-accrual IGRT guidance and assessment module**. *Clinical and Translational Radiation Oncology* 2019, **19**:27-32.

For peer review only



For peer review only - http://bmjopen.bmj.com/site/about/guidelines.xhtml

**Figure 1. Trial schema**

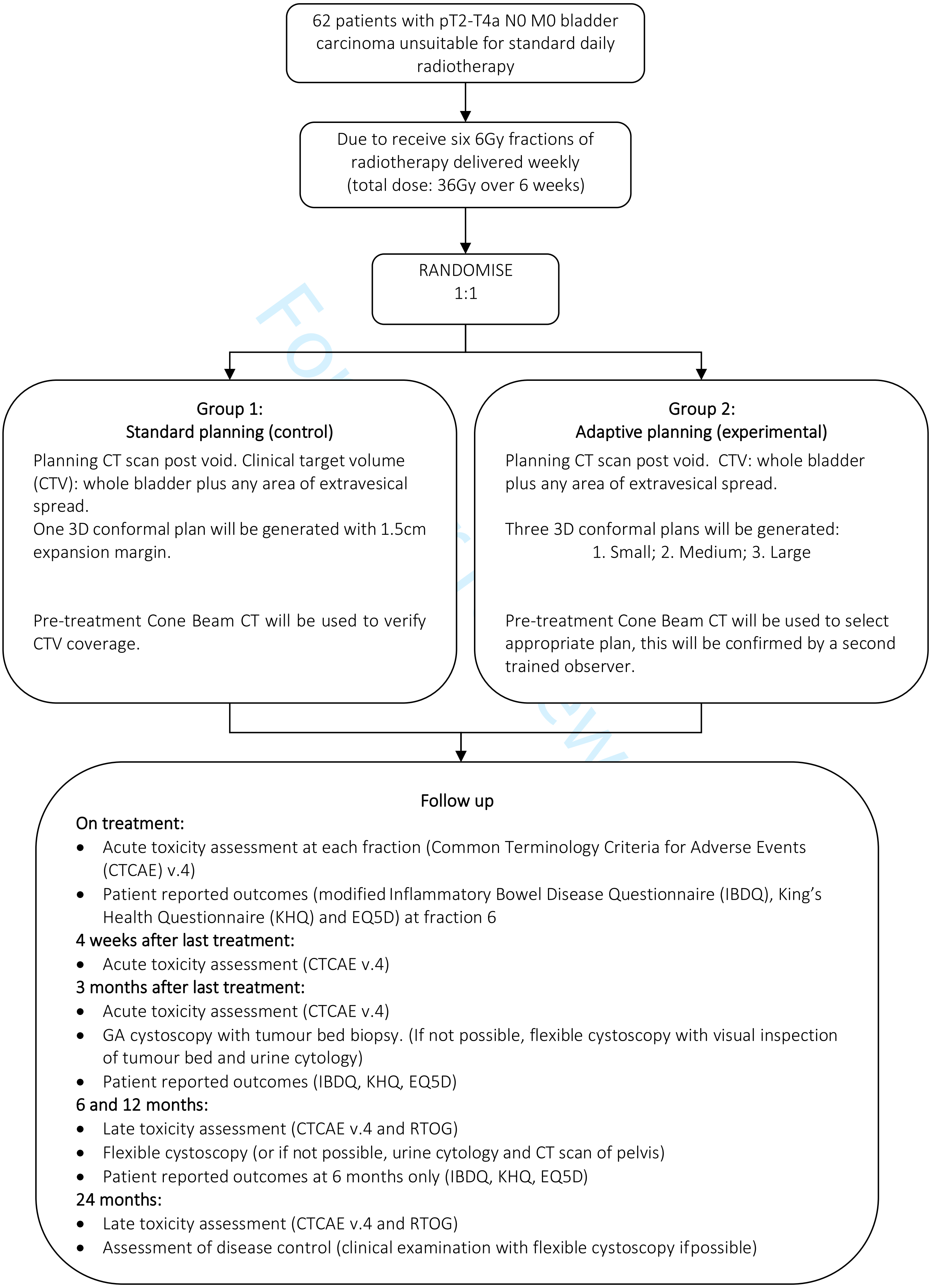

Please print on hospital headed paper

# HYBRID

A multicentre randomised phase II study of **Hy**pofractionated **B**ladder **R**adiotherapy with or without **I**mage guided a**d**aptive-predictive planning

## PATIENT INFORMATION SHEET

Version 1.1; 24 September 2013

We would like to invite you to take part in a research study called HYBRID.

Before you decide whether to take part, it is important that you understand why the research is being done and what it would involve for you. One of your doctors or nurses will go through this information sheet with you and answer any questions you may have . Please take time to read the information carefully and to discuss it with relatives, friends and your GP if you wish. Please ask if anything is unclear or you need any further information.

Thank you for reading this and considering taking part in this research.

**Why am I being invited to take part?**
We are inviting you to join this study because your doctor has found cancer that has grown into the wall of your bladder. Treatment for this type of bladder cancer would usually be surgery to remove the bladder or radiotherapy given in small doses every day for 4 or 7 weeks.

In your case, your doctor does not think that major surgery is a suitable treatment for you and you have agreed with your doctor that coming to hospital for radiotherapy treatment every day for 4 or 7 weeks would be difficult. As an alternative s/he is recommending that you are treated with radiotherapy given once a week for six weeks.

**What is radiotherapy treatment?**
Radiotherapy uses targeted beams of high strength x-rays to kill cancer cells. Because radiotherapy can also cause damage to normal, non-cancer cells, the treatment is carefully planned by doctors and physicists at your hospital so that only your bladder and a small border surrounding it is exposed to the highest radiotherapy dose.

Radiotherapy treatment is individually designed for each patient, based on a CT scan taken a few weeks before treatment which tells us about the position and shape of the bladder. The bladder can move about within the body depending on how full it is and because of where it is in relation to the bowel. It is important that the radiotherapy does not miss any of the bladder because of this movement, so we add a safety margin around the bladder on the planned treatment, to reduce the risk of the highest doses of radiotherapy missing any of the bladder.

Each patient would usually have just one radiotherapy plan designed for them before they start treatment. When radiotherapy treatment is given, the patient has to lie still on a bed whilst the radiotherapy machine moves around to send the radiotherapy beams from

For peer review only

*(Form to be printed on headed paper)*

different directions. These beams all focus on where the bladder is, to make sure that it receives the highest radiotherapy dose possible.

**What is adaptive radiotherapy treatment?**

We are now able to take a scan of where the bladder is when a patient is lying on the radiotherapy bed before each treatment. This means that we can give radiotherapy more accurately. In this study we are looking at whether it is possible to design three treatment plans with different size safety margins and then choose the one that fits best for each particular patient on their treatment day. This is called ‘adaptive radiotherapy’.

Adaptive radiotherapy may allow treatment to be given with smaller safety margins, resulting in reduced side effects. The bladder would still receive the highest radiotherapy dose, but reducing the amount of radiotherapy to the margins could reduce non-bladder side effects.

**What is the purpose of HYBRID?**

Many people with bladder cancer find daily radiotherapy for a number of weeks difficult to cope with. One radiotherapy treatment a week for six weeks may be a good option for people who would find it difficult to come to the hospital every day and this type of radiotherapy is already in use at most hospitals which are taking part in the HYBRID research study.

We hope to show that adaptive radiotherapy reduces non-bladder side effects compared to when radiotherapy is given using the same plan each time. We also want to gather more information about how well bladder cancer is treated by weekly radiotherapy, and how well it reduces any symptoms patients experience as a result of bladder cancer.

**What would happen if I took part?**

All participants in this study will be treated with 6 radiotherapy treatments given once weekly for 6 weeks.

Everyone who agrees to take part in this research study will be allocated at random to one of two groups. Half of the people taking part will receive weekly radiotherapy using the same treatment plan each time and half of participants will have weekly adaptive radiotherapy, using one of three plans. The only way to make sure that the two groups are as similar as possible is to have the treatment decided upon by chance: a process called randomisation. This process ensures that the treatments are compared fully and fairly.

If you agree to take part, your doctor or nurse will ring the research centre. The centre will then record your details and tell your specialist your treatment, which will be selected by chance, meaning you will have an equal chance of having either of the treatments. Whichever group you are in, you will be treated with the best possible care and will be monitored closely.

Both treatment groups will have a scan before radiotherapy treatment to make sure that the radiotherapy treatment will not miss any of the bladder.

<u>What do I have to do before my radiotherapy treatment?</u>

To make sure that your treatment is as effective as possible, it has to be carefully planned by your Doctor and other specialised staff (radiographers and physicists). It is a very



For peer review only - http://bmjopen.bmj.com/site/about/guidelines.xhtml

*(Form to be printed on headed paper)*

precise treatment and it is important that you are able to lie in exactly the same position for every treatment.

The planning session at the radiotherapy department usually takes place once and will last about 30 minutes. You will have a CT scan taken with your bladder empty. The radiographers will also take measurements from you that are needed for treatment planning. All of the planning procedures are part of the routine care for patients receiving bladder radiotherapy, so you will have them even if you choose not to take part in the HYBRID research study.

<u>What do I have to do during my radiotherapy treatment?</u>

Your treatment will be given once a week for six weeks. We will ask you to empty your bladder immediately before each treatment. Once the radiographer has helped you to get into position and made sure that you are comfortable, we will take a scan in the treatment room. This will take about 2 minutes.

For patients receiving the same treatment plan each time, this scan will be used to make sure that the bladder is in the area which will receive the highest dose of radiotherapy. If you are receiving adaptive radiotherapy we will use the information from the pre-treatment scan to study the bladder and choose the best plan to fit your bladder size and position. This will take around five to ten minutes. Once the plan has been selected by a specially trained doctor or radiographer and checked by a second trained person you will receive your radiotherapy treatment.

The treatment only takes a few minutes, but you will need to lie still for approximately 20 minutes whilst the machine moves around to deliver the radiotherapy from different angles. You will not feel anything, as it is similar to having an x-ray.

<u>How many times will I need to visit the hospital during and after my treatment?</u>

You will be seen regularly by your Doctor and/or nurse during and after treatment so that the side effects and effectiveness of the treatment can be measured.

- During your radiotherapy treatment you will be seen by your Doctor and/or nurse every week to record and treat any side effects that you may be experiencing and they will take a small sample of blood before treatment starts and on the second, fourth and sixth treatment visit.
- After your treatment you will be seen 4 weeks and 3, 6, 12 and 24 months after the end of your radiotherapy to record and treat any side effects, if present, and check how well the treatment has controlled your cancer. You will be asked to have a cystoscopy (inspection of your bladder with a telescope) to check your bladder 3, 6 and 12 months after your radiotherapy. If you are unable to have any of these tests we would ask that you have a CT scan and provide a sample of urine so your doctor can test it for the presence of cancer cells. If your cancer is found to have returned when these checks are done, your doctor will discuss available treatment options with you.

<u>Will I be asked to do anything else?</u>

The main reason we are carrying out the HYBRID study is to look at the side effects of the radiotherapy treatment. If you decide to take part in HYBRID, we would like you to complete short questionnaires to describe any side effects that you may experience.



For peer review only - http://bmjopen.bmj.com/site/about/guidelines.xhtml

*(Form to be printed on headed paper)*

This is an optional part of the study but completed questionnaires will help us to understand more about the side effects of this radiotherapy treatment from your point of view. Completing a questionnaire should take no longer than 20 minutes.

If you agree to take part, we will ask you to fill in a questionnaire before you start radiotherapy, at the end of your radiotherapy treatment and then twice more, at 3 and 6 months afterwards. We know from other patients that they feel such surveys are very important, but you do not have to complete them if you do not want to.

**What are the possible side effects of treatment?**
Patients who have radiotherapy commonly experience some side effects. These can occur in anyone receiving radiotherapy to the bladder whether or not they are in HYBRID. No one can predict whether you will have some, all or none of the side effects, or how severe they may be. They are usually mild and short lived but can sometimes be more serious. Please let your doctor or nurse know about any side effects that you are concerned about so they can advise you what to do. Their telephone numbers are at the end of this information sheet (p7). There is also 24 hour support available from your hospital, to provide access to immediate medical care in the event of any serious problems.

**Not all people will experience all of these side effects and we can give you medications to treat any side effects that you do experience.**

You will be able to carry out most of your normal activities during radiotherapy, but you may feel more tired than normal and may need to rest more.

Side effects can develop during radiotherapy that may include:

- diarrhoea (around 3 in 10 people)
- needing to urinate more often (around 3 in 10 people)
- bleeding, pain or discomfort on passing urine (around 2 in 10 people)
- passing stools more frequently or with pain (around 1 in 10 people)

Most people return to normal after radiotherapy but a few may develop long term effects. These are usually mild but can occasionally be serious and can require treatment.

Side effects which can develop after radiotherapy include:

- a need to urinate more often or more urgently (around 2 in 10 people)
- bowel changes due to scarring or bleeding (around 5 in 100 people)
- vaginal scarring (around 3 in 10 women)
- problems with getting and maintaining erections (around 2 in 10 men)
- infertility (around 5 in 10 people)

**Do I have to take part?**
No, it is up to you to decide whether to take part or not. If you decide to take part, you will be given this information sheet to keep and will be asked to sign a consent form. You are free to change your mind and withdraw from the study at any time without giving a reason. If you do choose to withdraw, your doctor will discuss with you the best treatment option available for you at that time.


For peer review only - http://bmjopen.bmj.com/site/about/guidelines.xhtml

*(Form to be printed on headed paper)*

**What are the alternatives to this study?**
Participation in this study will not affect the usual standard of care you receive. There are no standard recommended treatments for patients who cannot have daily radiotherapy or surgery for bladder cancer. If you do not take part in HYBRID your doctor will discuss any alternative options with you.

**What are the possible benefits of taking part?**
Weekly radiotherapy may be a more effective treatment for the cancer than any alternative treatments. Everyone in the study will receive a scan before their treatment. This may make the radiotherapy more accurate than if it was given without the scan. If you are in the adaptive radiotherapy group you will receive radiotherapy treatment with the smallest possible safety margin each time and this may reduce the risk of non-bladder side effects.

You may be seen at your hospital more often, and have more cystoscopies or CT scans after you finish radiotherapy than you would have had if you were not in HYBRID. Your doctor will explain whether this is the case at your hospital. This may be beneficial in that any side effects or return of the cancer can be found and treated more quickly than they would otherwise.

**What are the possible disadvantages of taking part?**
The effects of this type of weekly radiotherapy treatment are not completely known. Early studies suggest the side effects of the treatment are similar to daily treatment. One of the purposes of this study is to confirm these reports. It is possible that the side effects of weekly radiotherapy might be worse than for daily treatment, but this will be monitored for all HYBRID participants and the study will be stopped if people are experiencing bad side effects.

The selection and confirmation of a treatment plan will extend the length of each radiotherapy treatment by about 5 to 10 minutes for patients receiving adaptive radiotherapy.

If you will have more cystoscopies or CT scans after you finish radiotherapy than you would have if you were not in HYBRID you will need to attend hospital more often than you would otherwise. If you have more CT scans than you would if you did not take part in HYBRID you will be exposed to more radiation than you would otherwise.

Before participating you should consider if this will affect any insurance you have and seek advice if necessary.

**How will confidentiality be maintained?**
Your medical notes will be seen by authorised members of the research team at your hospital, so that they can collect information needed for the HYBRID study. When you join the study, your name, date of birth, postcode, hospital number and NHS or Community Health Index (CHI) number will be passed to the Institute of Cancer Research Clinical Trials and Statistics Unit (ICR-CTSU) where the study is being coordinated. You will be given a unique registration number, which will be used together with your initials and date of birth on forms that the research staff will send to the trials office. All information about you will be coded with the registration number and will be stored securely. It will be treated


For peer review only - http://bmjopen.bmj.com/site/about/guidelines.xhtml

*(Form to be printed on headed paper)*

as strictly confidential and nothing that might identify you will be revealed to any third party.

Scientific employees of ICR-CTSU, and those conducting the study with them, including the national radiotherapy quality assurance team, may need to examine your medical records to ensure the study is being run properly and that the information collected on the forms is correct, but your confidentiality will be protected at all times.

We will contact your hospital over the years to find out how you are getting on. Ideally we would like to do this for life, but patients often change address and/or GP or lose touch with their hospital. If this happens we would like to use national records which are kept on everyone's health status to find out how you are. One of these is held at the General Register Office (GRO). We will need to give them enough information to identify you. This is usually your name, date of birth and NHS number (or Community Health Index and/or hospital number in Scotland). Any details we receive from any source are confidential and will only be used for the purposes of the HYBRID study. Please initial the consent form to show that we have your permission to do this – if you do not agree, we will not seek this information.

All the information that is sent to the ICR-CTSU will be kept until 20 years after the HYBRID study has ended.

Data sharing

The organisers of this study would like to be able to combine information we collect about patients in this study with information collected for other studies, if in the future it could advance our knowledge of the treatment of cancer. If this happens, information about you may be passed to other legitimate researchers, but they would not be able to identify you from the information provided.

**What will happen to the results of the research study?**

Independent experts will review the progress of the research, and the results will be published in a respected medical journal once we are sure they are reliable. No information that could identify you will be included and you will not be identified in any report or publication.

We will summarise the results for participants once they are available. Your hospital will be able to give you a copy and results will also be available on Cancer Research UK's patient website (www.cancerhelp.org.uk).

**What if relevant new information becomes available?**

Sometimes we get new information about the treatment being studied. If this happens, your doctor will tell you and discuss whether you should continue in the study. If you decide not to carry on, your doctor will make arrangements for your care to continue.

**Will I be paid for taking part in this study?**

No. Neither you nor your doctor will be paid for taking part in this study.

**What if there is a problem?**

Any complaint about the way you have been dealt with during this study, or any possible harm you might suffer, will be addressed. Your progress will be watched closely and you



For peer review only - http://bmjopen.bmj.com/site/about/guidelines.xhtml

*(Form to be printed on headed paper)*

will be offered whatever help is available to cope with any side effects. Occasionally some patients need a short stay in hospital for side effects to be treated, and on rare occasions these can be serious. If this were to happen, full details of what has happened will be reviewed carefully by the Doctor who has overall responsibility for the HYBRID trial. It is unlikely that anything will go wrong with your treatment or care, but if you wish to complain about any aspect of the way you have been approached or treated during the course of the study you can do so using the normal NHS complaints procedure. Concerns should be raised by speaking to a member of staff at your hospital or by talking to the local Patient Advice and Liaison Service (PALS) which has been established in every NHS Trust.

NHS bodies are liable for clinical negligence and other negligent harm to individuals covered by their duty of care. In the event that something does go wrong and you are harmed during the research and this is due to someone‘s negligence then you may have grounds for a legal action for compensation against the NHS Trust but you may have to pay your legal costs. Alternative indemnity arrangements apply to private clinics.

**What if I don't want to carry on with the study?**
You are free to withdraw from the study at any time. You do not have to give a reason and your future treatment and care will not be affected. If you change your mind about having the treatment in this study, we would still like to collect information about how you are getting on. The information we need is routinely recorded in your medical records at your standard hospital visits and you would not need to do anything.

**Who is organising and funding the research?**
HYBRID is organised by leading doctors at the Royal Marsden Hospital in London and Sutton together with the Institute of Cancer Research in Sutton, Surrey. The research is approved and funded by Cancer Research UK. The National Health Service Research and Development Executive will pay for any extra nursing and administrative costs incurred by the hospitals.

**Who reviewed this study?**
All research in the NHS is looked at by an independent group of people, called a Research Ethics Committee, to protect participants' safety, rights, wellbeing and dignity. HYBRID has been reviewed and approved by London Surrey Borders Ethics Committee on behalf of all hospitals throughout the UK. It has also been reviewed and approved by Cancer Research UK and
reviewed and endorsed by patient and carer representatives from the NCRI Consumer Liaison Group (www.ncri.org.uk).

**What happens now?**
You will have some time to think about the study and make your decision. Your doctor or nurse will be happy to answer any questions. You may wish to discuss it with your family or friends. Once you have reached your decision please let your doctor or nurse know. You will be asked to sign a consent form and will be given a copy to keep together with this information sheet. Please keep this information sheet and copies of the signed consent form. Your GP will be told that you are taking part in the HYBRID study. If at any time you have any questions about the study you should contact your hospital consultant.

**Further information**



For peer review only - http://bmjopen.bmj.com/site/about/guidelines.xhtml

*(Form to be printed on headed paper)*

Macmillan Cancer support is a registered charity and helps with all the things that people affected by cancer want and need, from specialist health care and information to practical, emotional and financial support (www.macmillan.org.uk). You can also learn more about clinical trials on the Cancer Research UK's patient website (www.cancerhelp.org.uk).

**Contact details**
If at any time you have any questions about the study you should contact your local study team:
Local consultant's name:
Local research nurse/radiographer:
Address:
Telephone:
24 hour contact number:

Thank you for your interest in our research.

For peer review only



For peer review only - http://bmjopen.bmj.com/site/about/guidelines.xhtml

*(Form to be printed on headed paper)*

MREC Number: 13/LO/1350
HYBRID trial ID:

**CONSENT FORM**

**HYBRID:**
A multicentre randomised phase II study of **Hy**pofractionated **B**ladder **R**adiotherapy with or without **I**mage guided a**d**aptive-predictive planning

Name of Researcher taking consent:

**Please write your initials in the box to the right of each statement if you agree, and please sign at the bottom**

1. I confirm that I have read and understand the patient information sheet version 1.1 dated 24 Sept 2013 for the above study. I have had the opportunity to consider the information, ask questions and have had these answered satisfactorily. ☐
2. I understand that my participation is voluntary and that I am free to withdraw at any time without giving any reason, without my medical care or legal rights being affected. ☐
3. If I withdraw from the study, I consent to my doctor providing authorised researchers with basic clinical information that would be routinely collected and written in my medical records. ☐
4. I understand that sections of any of my medical notes may be looked at by responsible individuals from the research team, from ethics committees, or from the NHS Trust, where it is relevant to my taking part in research. I give permission for these individuals to have access to my records. ☐
5. I consent to the Institute of Cancer Research using information held by the NHS and national databases to follow up my health status. ☐
6. Data sharing: I grant advance authorisation for the possible future sharing of information collected about me with other organisations, with the understanding that I will not be identifiable from this information *(optional)*. ☐
7. I agree to my GP being informed of my participation in the study. ☐
8. I agree to participate in the side effects questionnaire study. *(If the answer to this question is 'NO', you may still take part in HYBRID)* ☐
9. I agree to take part in HYBRID. ☐

| ……………………………… | ……………… | ……………………………… |
|---|---|---|
| Name of participant | Date | Signature |
| Name of person taking consent (if different from researcher) | Date | Signature |
| Researcher (PI) | Date | Signature |

1 copy for participant; 1 copy for Principal Investigator; 1 copy to be kept with hospital notes



For peer review only - http://bmjopen.bmj.com/site/about/guidelines.xhtml

# HYBRID

## A multicentre randomised phase II study of **HY**pofractionated **B**ladder **R**adiotherapy with or without **I**mage guided a**D**aptive planning

**PROTOCOL**

Version: 3.1

Dated: 13/08/2015

| | |
|---|---|
| Chief Investigator: | Professor Robert Huddart |
| Sponsor: | The Institute of Cancer Research |
| Approval: | Cancer Research UK: Clinical Trials Awards & Advisory Committee (CTAAC) |
| Funders: | Cancer Research UK |
| Coordinating Trials Unit: | ICR Clinical Trials and Statistics Unit (ICR-CTSU) |
| | The Institute of Cancer Research |

| | |
|---|---|
| Main REC Reference Number: | 13/LO/1350 |
| ICR-CTSU Protocol Number: | ICR-CTSU/2013/10039 |
| ISRCTN: | ISRCTN18815596 |
| ClinicalTrials.gov Identifier | NCT01810757 |
| CRUK Reference Number: | CRUK/12/055 |

The HYBRID trial has been scientifically approved
by Cancer Research UK's Clinical Trials Awards & Advisory Committee (CTAAC)
The HYBRID trial is part of the National Institute for
Health Research Clinical Research Network Trial Portfolio

ICR The Institute of Cancer Research

CANCER RESEARCH UK

This protocol is a controlled document and should not be copied, distributed or reproduced without the written permission of the ICR-CTSU



For peer review only - http://bmjopen.bmj.com/site/about/guidelines.xhtml

# ADMINISTRATION

## Scientific Coordination

Professor Robert Huddart (Chief Investigator)
Academic Radiotherapy/Division of Radiotherapy and Imaging
The Royal Marsden NHS Foundation Trust/The Institute Of Cancer Research
London
UK

Tel: 0208 661 3457
Robert.Huddart@icr.ac.uk

Shaista Hafeez (Clinical Coordinator)
The Royal Marsden NHS Foundation Trust/The Institute Of Cancer Research
London
UK

Tel: 0208 661 1143
Shaista.Hafeez@rmh.nhs.uk

## Trial Coordination

ICR-CTSU (a UKCRC registered NCRI cancer clinical trials unit) is responsible for the day to day conduct of the trial.

ICR Clinical Trials & Statistics Unit (ICR-CTSU),
Division of Clinical Studies,
The Institute of Cancer Research,
Sir Richard Doll Building,
Cotswold Road,
Sutton, Surrey SM2 5NG

ICR-CTSU Scientific Lead : Emma Hall
Tel: 020 8722 4013
Emma.Hall@icr.ac.uk

ICR-CTSU Statistician: Clare Griffin/Helen Mossop
Tel: 020 8722 4062
HYBRID-icrctsu@icr.ac.uk

ICR-CTSU Senior Trial Manager: Rebecca Lewis
Tel: 020 8722 4081
HYBRID-icrctsu@icr.ac.uk

HYBRID Trial Manager: Joanna Illambas
Tel: 020 8722 4295
HYBRID-icrctsu@icr.ac.uk

Any questions relating to this protocol should be addressed in the first instance to the HYBRID Trial Manager within ICR-CTSU:

Email: HYBRID-icrctsu@icr.ac.uk
General enquiries: 020 8722 4238
Fax: 020 8770 7876

 For peer review only - http://bmjopen.bmj.com/site/about/guidelines.xhtml

For peer review only

## PROTOCOL DEVELOPMENT GROUP

| | | |
|---|---|---|
| Professor Robert Huddart | Chief Investigator | The Institute of Cancer Research/Royal Marsden NHSFT |
| Dr Emma Hall | Deputy Director | ICR-CTSU, The Institute of Cancer Research, London |
| Dr Ann Henry | Consultant Oncologist | Leeds University Teaching Hospitals |
| Dr Vincent Khoo | Consultant Oncologist | Royal Marsden NHS Foundation Trust |
| Dr John Staffurth | Consultant Oncologist | Velindre NHS Trust |
| Dr Isabel Syndikus | Consultant Oncologist | Clatterbridge Centre for Oncology |
| Dr Vibeke Hansen | Physicist | Royal Marsden NHS Foundation Trust |
| Dr Helen McNair | Research Radiographer | Royal Marsden NHS Foundation Trust |
| Dr Shaista Hafeez | Research Fellow | Institute of Cancer Research/ Royal Marsden NHSFT |
| Clare Griffin | Statistician | Institute of Cancer Research |
| Rebecca Lewis | Senior Trials Manager | Institute of Cancer Research |

The Trial Management Group (TMG) will be constituted from members of the Protocol Development Group and Principal Investigators from a subset of participating centres. A copy of the current membership of the TMG can be obtained from the HYBRID Trial Manager at ICR-CTSU.

Protocol Authorised by:

| Name & Role | Signature | Date |
|---|---|---|
| Professor Robert Huddart<br>(Chief Investigator) | 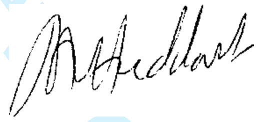 | 13/08/2015 |

This protocol describes the HYBRID trial and provides information about procedures for entering participants into this trial. The protocol should not be used as a guide for the treatment of patients outside of this trial.

Every care was taken in the preparation of this protocol, but corrections or amendments may be necessary. Protocol amendments will be circulated to participating sites as they occur, but sites entering patients for the first time are advised to contact ICR-CTSU to confirm they have the most recent version.



For peer review only - http://bmjopen.bmj.com/site/about/guidelines.xhtml

CONTENTS

1. INTRODUCTION ........ 10
1.1. Background ........ 10
1.1.1. Standard treatment for muscle invasive bladder cancer ........ 10
1.1.2. Management of muscle invasive bladder cancer in patients unfit for standard treatment ... 10
1.1.3. Evidence for hypofractionated radiotherapy in bladder cancer ........ 10
1.2. Challenges to ensuring accurate bladder radiotherapy delivery ........ 11
1.3. Image guided radiotherapy (IGRT) ........ 11
1.3.1. Evidence for adaptive-planned image guided radiotherapy (IGRT) ........ 11
1.4. Known risks and benefits of adaptive hypofractionated radiotherapy ........ 12
1.4.1. Potential benefits ........ 12
1.4.2. Potential risks ........ 12
1.5. Study rationale ........ 13
2. TRIAL OBJECTIVES ........ 13
2.1. Primary objective ........ 13
2.2. Secondary objectives ........ 13
2.3. Exploratory objectives ........ 13
3. TRIAL DESIGN ........ 13
4. STUDY ENDPOINTS ........ 14
4.1. Primary endpoint ........ 14
4.2. Secondary endpoints ........ 14
4.3. Exploratory endpoints ........ 14
5. PATIENT SELECTION & ELIGIBILITY ........ 15
5.1. Number of participants ........ 15
5.2. Source of participants ........ 15
5.3. Inclusion criteria ........ 15
5.4. Exclusion criteria ........ 15
5.5. Lifestyle guidelines ........ 15
6. SCREENING ........ 15
6.1. Screening log ........ 15
6.2. Procedure for obtaining informed consent ........ 16
6.3. Participation in other research ........ 16
7. RANDOMISATION ........ 16
8. TRIAL ASSESSMENTS ........ 17
8.1. Pre-randomisation assessments ........ 17
8.2. Pre-treatment assessments ........ 17
8.3. On-treatment assessments ........ 17
8.4. Post radiotherapy assessments ........ 17
8.4.1. 4 weeks from last radiotherapy fraction ........ 17


For peer review only - http://bmjopen.bmj.com/site/about/guidelines.xhtml

8.4.2. 3 months from last radiotherapy fraction ... 17
8.4.3. 6 and 12 months (from last radiotherapy fraction) ... 17
8.4.4. 24 months (from last radiotherapy fraction) ... 18
8.4.5. Annually thereafter ... 18
8.5. Procedure at disease progression/recurrence ... 18
8.6. Withdrawal from treatment or follow-up ... 18
9. SCHEDULE OF ASSESSMENTS ... 19
10. TREATMENT ... 20
10.1. Standard pre-trial treatment ... 20
10.2. Treatment timelines ... 20
10.3. Radiotherapy planning and delivery ... 20
10.4. Treatment scheduling and gaps ... 20
10.5. Supportive care guidelines ... 20
10.6. Concomitant therapy ... 20
11. RADIOTHERAPY QUALITY ASSURANCE (QA) ... 21
12. SAFETY REPORTING ... 21
12.1. Adverse event (AE) ... 21
12.2. Serious adverse event (SAE) ... 21
12.3. Serious Adverse Reaction (SAR) ... 22
12.3.1. Definitions of causality ... 22
12.4. Related Unexpected SAE ... 22
12.5. Reporting Adverse Events to ICR-CTSU ... 22
12.6. Reporting Serious Adverse Events to ICR-CTSU ... 23
12.7. Serious Adverse Events exempt from expedited reporting ... 23
12.8. Review of Serious Adverse Events ... 23
12.9. Expedited Reporting of Related Unexpected SAEs ... 24
12.10. Follow up of Serious Adverse Events ... 24
12.11. Annual reporting of safety considerations ... 24
13. STATISTICAL CONSIDERATIONS ... 24
13.1. Statistical design and sample size justification ... 24
13.2. Treatment allocation ... 25
13.3. Primary endpoint definition ... 25
13.4. Secondary endpoint definitions ... 25
13.4.1. Local disease control at 3 months ... 25
13.4.2. Control rate of presenting symptoms ... 25
13.4.3. Patient reported outcomes (PRO) ... 25
13.4.4. Late toxicity ... 25
13.4.5. Time to local disease progression ... 25

For peer review only


For peer review only - http://bmjopen.bmj.com/site/about/guidelines.xhtml

13.4.6. Overall survival ... 26
13.4.7. Proportion of fractions benefiting from adaptive planning ... 26
13.4.8. Appropriate identification and correction of fractions requiring adaptive planning ... 26
13.5. Exploratory endpoints ... 26
13.6. Analysis plan ... 26
13.7. Interim analyses and stopping rules ... 27
14. TRIAL MANAGEMENT ... 27
14.1. Trial Management Group (TMG) ... 27
14.2. Trial Steering Committee (TSC) ... 27
14.3. Independent Data Monitoring Committee (IDMC) ... 27
15. RESEARCH GOVERNANCE ... 28
15.1. Sponsor responsibilities ... 28
16. TRIAL ADMINISTRATION & LOGISTICS ... 28
16.1. Site activation ... 28
16.2. Investigator training ... 28
16.3. Data acquisition ... 28
16.4. Central data monitoring ... 28
16.5. On-site monitoring ... 29
16.6. Completion of the study and definition of study end date ... 29
16.7. Archiving ... 29
17. PATIENT PROTECTION AND ETHICAL CONSIDERATIONS ... 29
17.1. Trial approvals ... 29
17.2. Trial conduct ... 29
17.3. Informed consent ... 29
17.4. Patient confidentiality ... 29
17.5. Data Protection Act (DPA) ... 30
17.6. Liability ... 30
18. FINANCIAL MATTERS ... 30
19. PUBLICATION POLICY ... 30
20. ASSOCIATED STUDIES ... 30
20.1. Patient reported outcome measures study ... 30
21. REFERENCES ... 31
A1. WHO performance status ... 33
A2. RTOG/EORTC late radiation morbidity scoring schema ... 33
A3. PATIENT REPORTED OUTCOMES STUDY ... 34
A3.1 Background ... 34
A3.2 Hypothesis ... 34
A3.3 Quality of life measures ... 34



For peer review only - http://bmjopen.bmj.com/site/about/guidelines.xhtml

A3.4 Study design ... 34
A3.5 Timing of data collection ... 35
A3.6 Compliance ... 35
A3.7 Statistical considerations ... 35
A4. GLOSSARY ... 36

For peer review only


For peer review only - http://bmjopen.bmj.com/site/about/guidelines.xhtml

## HYBRID TRIAL SUMMARY

| | |
|---|---|
| PROTOCOL TITLE | A multicentre randomised phase II study of **Hy**pofractionated **B**ladder **R**adiotherapy with or without **I**mage guided a**D**aptive planning |
| TARGET DISEASE | Muscle invasive bladder cancer |
| STUDY OBJECTIVES | The primary objective of HYBRID is to assess whether adaptive radiotherapy techniques when delivered at multiple centres can lead to a reduction in the level of acute non-genitourinary side effects experienced by patients with muscle invasive bladder cancer unsuitable for daily radical radiotherapy. |
| STUDY DESIGN | Multicentre phase II randomised controlled trial |
| TRIAL POPULATION | Patients with muscle invasive bladder cancer who are not suitable for cystectomy or daily radiotherapy |
| RECRUITMENT TARGET | 62 |
| TRIAL TREATMENT | Hypofractionated radiotherapy - all patients will be planned to receive six 6 Gray (Gy) fractions of radiotherapy delivered weekly (total dose: 36Gy) and will be randomised between either standard or adaptive planning. |
| PRIMARY ENDPOINT | Acute non-genitourinary grade 3 or greater toxicity (assessed using Common Terminology Criteria for Adverse Events (CTCAE) v.4) |
| SECONDARY ENDPOINTS | • Local disease control at 3 months<br>• Control rate of presenting symptoms (change from pre-radiotherapy CTCAE grades)<br>• Patient reported outcomes<br>• Late toxicity<br>• Time to local disease progression<br>• Overall survival<br>• Proportion of fractions benefiting from adaptive planning |
| EXPLORATORY ENDPOINTS | • Appropriate identification and correction of fractions requiring adaptive planning<br>• Dose Volume Histogram analysis of clinical target volume (CTV) coverage using anisotropic margins<br>• Concordance of clinician and patient reported toxicity measures |
| FOLLOW UP | Participants will be assessed for acute toxicity at each treatment visit and will complete a Patient Reported Outcomes (PRO) questionnaire at fraction 6. Participants will subsequently be assessed at the following intervals:<br>**4 weeks from end of radiotherapy:**<br>Assessment of acute toxicity (CTCAE v.4)<br>**3 months from end of radiotherapy:**<br>Assessment of acute toxicity (CTCAE v.4) and biopsy of tumour bed |


For peer review only - http://bmjopen.bmj.com/site/about/guidelines.xhtml

under general anaesthetic (if not possible, flexible cystoscopy with visual inspection of tumour bed and urine cytology). PRO questionnaire.

**6 & 12 months from end of radiotherapy:**

Late toxicity assessment (CTCAE v.4 and RTOG) and flexible cystoscopy to visually assess local control (if not possible, pelvic CT scan and urine cytology). PRO questionnaire at 6 month visit only.

**24 months from end of radiotherapy:**

Late toxicity assessment (CTCAE v.4 and RTOG)

Assessment of disease control by clinical examination and flexible cystoscopy if possible.

For peer review only

 For peer review only - http://bmjopen.bmj.com/site/about/guidelines.xhtml

## TRIAL SCHEMA

62 patients with pT2-T4a N0 M0 bladder carcinoma unsuitable for standard daily radiotherapy

↓

Due to receive six 6Gy fractions of radiotherapy delivered weekly (total dose: 36Gy over 6 weeks)

↓

RANDOMISE

1:1

**Group 1:**
**Standard planning (control)**

Planning CT scan post void. Clinical target volume (CTV): whole bladder plus any area of extravesical spread.

One 3D conformal plan will be generated with 1.5cm expansion margin.

Pre-treatment Cone Beam CT will be used to verify CTV coverage.

**Group 2:**
**Adaptive planning (experimental)**

Planning CT scan post void. CTV: whole bladder plus any area of extravesical spread.

Three 3D conformal plans will be generated:
1. Small; 2. Medium; 3. Large

Pre-treatment Cone Beam CT will be used to select appropriate plan, this will be confirmed by a second trained observer.

↓

**Follow up**

**On treatment:**

- Acute toxicity assessment at each fraction (Common Terminology Criteria for Adverse Events (CTCAE) v.4)
- Patient reported outcomes (modified Inflammatory Bowel Disease Questionnaire (IBDQ), King's Health Questionnaire (KHQ) and EQ5D) at fraction 6

**4 weeks after last treatment:**

- Acute toxicity assessment (CTCAE v.4)

**3 months after last treatment:**

- Acute toxicity assessment (CTCAE v.4)
- GA cystoscopy with tumour bed biopsy. (If not possible, flexible cystoscopy with visual inspection of tumour bed and urine cytology)
- Patient reported outcomes (IBDQ, KHQ, EQ5D)

**6 and 12 months:**

- Late toxicity assessment (CTCAE v.4 and RTOG)
- Flexible cystoscopy (or if not possible, urine cytology and CT scan of pelvis)
- Patient reported outcomes at 6 months only (IBDQ, KHQ, EQ5D)

**24 months:**

- Late toxicity assessment (CTCAE v.4 and RTOG)
- Assessment of disease control (clinical examination with flexible cystoscopy if possible)


For peer review only - http://bmjopen.bmj.com/site/about/guidelines.xhtml

# 1. INTRODUCTION

## 1.1. Background

### 1.1.1. Standard treatment for muscle invasive bladder cancer

Bladder cancer is the seventh most common UK cancer with 10,324 cases diagnosed in 2010 (1) . Muscle invasive bladder cancer accounts for 25% of new tumours and is associated with poor survival (<50% at 5 years (2)). For patients of good performance status, standard management would involve surgical excision of the bladder (radical cystectomy) or a course of radical radiotherapy given daily over 4-7 weeks (3).

Incidence of muscle invasive bladder cancer increases with age and many patients are not fit enough for major surgery, with its associated high treatment related mortality (4-6). National Institute for Health and Clinical Excellence (NICE) guidelines (3) recommend that: 'Radical radiotherapy is appropriate for patients who are not sufficiently fit for surgery or who wish to avoid cystectomy'. In the UK, radical radiotherapy for muscle invasive bladder cancer is delivered daily either to a total dose of 64 Gray (Gy) in 32 fractions (f) over 6.5 weeks or 55 Gy in 20f over 4 weeks.

Radiotherapy is an established treatment for muscle invasive bladder cancer, providing long term local control and allowing the patient to preserve their intrinsic bladder function (2, 7, 8). Technological advances such as three dimensional conformal planning now permit radiation dose to be shaped around the target, avoiding organs at risk (9). By employing reduced safety margins around the target volume, dose to organs at risk can be further limited. These highly precise methods rely on the target being in the same position each time radiotherapy is delivered.

### 1.1.2. Management of muscle invasive bladder cancer in patients unfit for standard treatment

Data collected as part of the SPARE trial (CRUK/07/011, ISRCTN61126465) suggest that around 70% of patients presenting with muscle invasive bladder cancer are unsuitable for standard radical therapy (surgery or daily radiotherapy) (10). This population presents a management dilemma, with an unmet clinical need. Despite relatively poor performance status many such patients would have normal life expectancy of several years but left untreated would experience (or be at risk of experiencing) significant disease related symptoms such as haematuria, urinary frequency, dysuria, pelvic pain, urinary incontinence and urinary obstruction.

There is relatively little published literature on radical treatment options for patients unfit for standard daily treatment. In current practice, patients are normally treated with hypofractionated radiotherapy where fewer but larger fractions are given at each visit. Despite this palliative intent, a proportion of patients survive for two or more years (11), thus treating with a higher biological effective dose with the aim of achieving local control could be expected to improve both survival and quality of life.

### 1.1.3. Evidence for hypofractionated radiotherapy in bladder cancer

The only multicentre randomised study of hypofractionated radiotherapy in muscle invasive bladder cancer (MRCBA09) investigated palliative regimens with low effective biological doses, randomising 500 patients between 35Gy in 10f over 2 weeks or 21Gy in 3f over 1 week (11). The trial included patients of poorer prognosis than those who will be included in HYBRID; despite this both treatment groups achieved symptom control or improvement in over 65% of participants and local control was achieved in 18/33 (55%) and 14/37 (38%) participants in the 35Gy and 21Gy groups respectively.

Other groups have reported single centre retrospective series of hypofractionated weekly radiotherapy, most often using 6Gy per fraction to a dose of 30-36 Gy over 5-6 weeks(12-14). This fractionation schedule delivers a higher biological dose to the tumour than the BA09 schedules with late toxicity theoretically similar to that seen for a standard 2Gy/f schedule (11). The 2Gy equivalent dose using an $\alpha/\beta$ ratio of 10 for 35Gy/10f, 21Gy/3f and 36Gy/6f are 39.75, 29.75 and 48Gy respectively. Retrospective reports suggest that the 36Gy/6f schedule is well tolerated, with acute toxicity less than that of standard fractionated treatment and acceptable (but variable) late toxicity. The best estimate taken from across these studies

 For peer review only - http://bmjopen.bmj.com/site/about/guidelines.xhtml

might be Radiation Therapy Oncology Group (RTOG) grade 3-4 late toxicity rate of 5-10%. However, because of the retrospective collection of many of these data, reports may be subject to significant bias and potential underreporting of adverse outcomes, so prospective verification is important to confirm toxicity experienced by patients. Three month local control rates of >60% have been reported and again would benefit from prospective verification. Survival is similar to that which would be expected in this population, but all studies had a proportion of long term survivors. An attraction of a schedule where treatment is delivered weekly is that close monitoring of acute toxicity can be undertaken and if necessary radiotherapy can be modified to fit the patient's tolerance of the treatment. Prospective multicentre assessment of the 36Gy in 6 fractions weekly schedule has not been performed.

### 1.2. Challenges to ensuring accurate bladder radiotherapy delivery

A course of standard radiotherapy is planned using a CT scan taken when the patient has an empty bladder. Currently, each fraction of radiotherapy is delivered based on the target position on a snapshot CT image taken at the time of planning and knowledge of the bladder position with each treatment fraction is unknown. It is assumed that this initial scan is representative of bladder position throughout the course of treatment and one plan is generated by the radiotherapy physics department for use throughout treatment. Safety margins of 1.5-2cm are added to generate the planning target volume (PTV) to account for uncertainty introduced by microscopic disease not visible on the CT scan, errors in patient set up and day-to-day variation in bladder filling.

The bladder is a mobile, deformable structure however and bladder volume can vary markedly during a course of radiotherapy, despite delivering treatment to a perceived empty bladder (15-21). Movement of the bladder wall by more than 1.5cm has been documented in up to 60% of patients, resulting in inadequate coverage by radiotherapy fields in 33% (19). A study at the Royal Marsden Hospital (RMH) (22) reported that up to 57% of treatment may be delivered with some element of geographic miss (where the radiotherapy does not "hit" the tumour volume), despite employing safety margins of 1.5cm around the empty bladder (23). Geographical miss leads to the possibility of reduced tumour control, but larger margins would increase the treated volume and the amount of normal tissue exposed to high dose radiation, potentially leading to increased toxicity. Set-up uncertainties may be reduced through ensuring consistency of patient position, but bladder volume variation and displacement may still exist. An alternative method of assessing this and correcting for it is required.

### 1.3. Image guided radiotherapy (IGRT)

Recent advances enable images of soft tissue to be obtained within the radiotherapy treatment room using cone beam CT (CBCT) and other technologies. Although of lower resolution than the original planning CT scan, these can be used both to match bony anatomy automatically and to visualise bladder position, thus helping to ensure that the PTV is correctly delivered. The bladder is predicted to be one of the key tumour sites to achieve major benefits from IGRT techniques (13, 19) and in 2012 the National Radiotherapy Implementation Group recommended that volumetric imaging should be used for treatment verification for every patient receiving hypofractionated radiotherapy to the bladder (24).

#### 1.3.1. Evidence for adaptive-planned image guided radiotherapy (IGRT)

Studies of CBCT have ascertained the extent of geographic miss in bladder cancer patients (16, 18, 21, 23). In an imaging study in the patient population included in HYBRID, 42/83 (51%) fractions had some displacement of the cone beam target volume (PTVcb) outside of the planned PTV. An average of 8% (and up to a maximum of 35%) of the bladder PTV was missed (23). This study included retrospective treatment planning which showed that an adaptive radiotherapy approach would have achieved complete coverage in 73% of treatment fractions, with substantial improvements seen in the remainder. Geographic misses have a particularly high impact on hypofractionated schedules as one fraction of treatment delivered off target represents a significant proportion (15-20%) of the total dose. However increased margins would cause exposure of more normal tissue to large doses per fraction and could lead to increased toxicity, particularly in this less fit patient group. The challenge is to use this imaging technology and to implement IGRT for patient benefit. Two potential methodologies have been suggested:

 For peer review only - http://bmjopen.bmj.com/site/about/guidelines.xhtml

- Composite plan - offline assessments are used to produce a composite plan covering the range of possible bladder positions which are then covered by a smaller margin. This technique is not applicable in the context of hypofractionated radiotherapy as too few fractions are delivered to produce the composite plan.
- 'Plan of the day' methodology - at the planning stage, a series of treatment plans, rather than one, are created to cover a range of bladder filling and positional variability. A CBCT taken prior to treatment can be compared against the original planning scan to ascertain the possibility of any geographic miss after set up correction. If variation in bladder volume is demonstrated, an alternative plan can be selected to ensure the whole target is treated. This methodology reduces geographic misses, often allows selection of plans with smaller margins and is deliverable by radiography staff (23).

The 'plan of the day' concept has been evaluated in a non-randomised single centre phase II feasibility study at the RMH (Adaptive predictive planning for hypofractionated bladder radiotherapy: APPLY; ISRCTN80815524). Twenty-five patients unfit for radical daily radiotherapy were recruited to this study of hypofractionated adaptive radiotherapy (as radical or palliative treatment). Three treatment plans were generated for each participant; small, medium (standard) and large. 24 out of 25 patients completed their planned treatments and adaptive plans were selected for delivery of 55% of fractions (49% small, 6% large). Compared to standard planning this resulted in a 45% average reduction in PTV. Concordance between online and blinded offline plan selection was 91% (126/139), and there was 99% coverage of the CTV by the 95% isodose curve (25). With a median follow-up of 8.4 months in the 20 patients with localised disease at presentation, 9 (45%) patients were well with no sign of bladder carcinoma recurrence and 11 (55%) had died, 6 from bladder carcinoma and 5 from other causes. 5/20 (25%), 1/20 (5%) and 2/20 (10%) patients experienced CTCAE grade 3 acute genitourinary, gastrointestinal and other toxicities, respectively. 14 patients were assessable 6 months or more post-radiotherapy and of these, 4 and 1 patients experienced late grade 2 genitourinary and gastrointestinal toxicity, respectively. There were no grade 3 or higher late toxicities reported (26).

Adaptive planning does not reduce the volume of bladder irradiated but it is expected to reduce the volume of non-bladder tissue irradiated. As such, it is the non-genitourinary toxicity that is of primary interest with the expectation that there will be less toxicity associated with adaptive planning. Grade 3 and above is considered to be the toxicity level of interest as toxicities below this grade can be controlled with minimal intervention and have little impact on activities of daily living. Grade 3 toxicities are severe or medically significant but not immediately life-threatening, cause hospitalisation and can be disabling, limiting the self care capabilities of patients.

## 1.4. Known risks and benefits of adaptive hypofractionated radiotherapy

### 1.4.1. Potential benefits

It is anticipated that the use of adaptive radiotherapy techniques will improve the accuracy of treatment. This should lead to benefits for patients in terms of both reduced exposure of normal tissue to high doses of radiotherapy and an associated reduction in non-genitourinary side effects and also a reduced risk of geographic misses.

### 1.4.2. Potential risks

Hypofractionated radiotherapy uses larger doses per fraction and this may alter the side effect profile compared to standard radiotherapy. Toxicity data will be reviewed by the Independent Data Monitoring Committee throughout the trial and any concerns will be raised with the Trial Management Group as appropriate. Adaptive radiotherapy is more complex due to the need to select the most appropriate plan and thus extends treatment delivery time – treatment delivery time will be collected as part of the HYBRID dataset.

In addition, the use of CBCT leads to radiation exposure which is additional to that of standard radiotherapy delivered without the use of IGRT techniques. The risks of any resulting carcinogenic effect from CBCT are


For peer review only - http://bmjopen.bmj.com/site/about/guidelines.xhtml

considered minimal as the exposure represents <1% of the therapeutic radiation dose and the life expectancy of this group of patients is likely to be less than 10 years.

### 1.5. Study rationale

Patients with muscle invasive bladder cancer who are unable to receive daily radiotherapy often experience genitourinary symptoms which interfere with their daily life due to the burden of disease within their bladder. Although unsuitable for daily radiotherapy, these patients may otherwise have a life expectancy of several years. Treatment with hypofractionated radiotherapy at a dose of 36 Gy in 6 fractions would provide these patients the opportunity to attain local control of their tumour, with an associated reduction in symptoms. When hypofractionation is used, the precise delivery of each treatment is arguably even more important than in standard fractionation schedules, both to ensure maximal tumour control and minimise toxicity, as each treatment represents 15-20% of the total dose.

Improving radiotherapy quality is of clear importance in bladder cancer treatment. It is now important to assess whether the pilot work relating to adaptive radiotherapy conducted as part of single centre studies can be successfully translated into radiotherapy practice across the UK and to prospectively assess the benefits for patients as part of a multicentre randomised trial.

HYBRID is a multicentre study of the adaptive radiotherapy methodology in patients receiving weekly bladder radiotherapy and will provide the opportunity to standardise treatment for this patient group, allow the collection of prospective multicentre data on the 36 Gy in 6 fractions regimen, test the feasibility of delivering adaptive methodology in a number of NHS sites and assess whether this methodology provides patients with the opportunity to reduce radiotherapy related side effects.

## 2. TRIAL OBJECTIVES

### 2.1. Primary objective

The primary objective of HYBRID is to assess whether adaptive radiotherapy techniques when delivered at multiple centres can lead to a reduction in the level of acute non-genitourinary toxicity experienced by patients with muscle invasive bladder cancer unsuitable for daily radical radiotherapy.

### 2.2. Secondary objectives

Secondary objectives of HYBRID are to establish the local disease control rates of hypofractionated bladder radiotherapy as measured at 3 months, and assess time to local disease progression and the overall survival time of patients who have received hypofractionated radiotherapy. HYBRID will also investigate the control rate of presenting symptoms, the effect of hypofractionated treatment on late radiotherapy side effects and assess patient reported outcomes. The proportion of fractions benefiting from adaptive planning will also be assessed.

### 2.3. Exploratory objectives

HYBRID will measure the appropriate identification and correction of fractions requiring adaptive planning, the dose volume histogram analysis of CTV coverage using anisotropic margins and will explore the concordance of clinician and patient reported toxicity measures.

## 3. TRIAL DESIGN

HYBRID is a multicentre randomised controlled phase II trial in patients with localised muscle invasive bladder cancer who are unsuitable for daily radical radiotherapy treatment.

All patients will be planned to receive six 6Gy fractions of image guided radiotherapy delivered weekly (total dose: 36Gy) and will be randomised to standard or adaptive planning.

Participants allocated to the standard planning group will have one radiotherapy plan generated and this will be used to deliver all 6 treatments, with a cone beam CT scan prior to treatment delivery which can be used by the local investigator to adjust treatment delivery according to local practice.

 For peer review only - http://bmjopen.bmj.com/site/about/guidelines.xhtml

Participants allocated to adaptive planning will have three radiotherapy plans generated; small, medium and large. A cone beam CT taken prior to each treatment delivery will be used to select the most appropriate plan of the day.

Acute toxicity data will be collected throughout treatment and at 4 weeks and 3 months from the end of radiotherapy. Local control will be assessed by cystoscopy at 3, 6 and 12 months. Late toxicity and survival data will be collected at 6, 12 and 24 months, after this time only basic routine follow-up data will be collected. Participants will be asked to complete a patient reported outcomes questionnaire prior to treatment, at the end of treatment and at 3 and 6 months.

# 4. STUDY ENDPOINTS

## 4.1. Primary endpoint

- Acute non-genitourinary grade 3 or greater toxicity (CTCAE v.4) occurring during radiotherapy and up to 3 months following treatment completion

## 4.2. Secondary endpoints

- Local disease control rate at 3 months
- Control rate of presenting symptoms – change in CTCAE grades from pre-radiotherapy to 3 months following treatment completion
- Patient reported outcomes
- Late toxicity
- Time to local disease progression
- Overall survival
- Proportion of fractions benefiting from adaptive planning
- Appropriate identification and correction of fractions requiring adaptive planning by retrospective independent central review of scan and treatment data

## 4.3. Exploratory endpoints

- Dose Volume Histogram analysis of CTV coverage using anisotropic margins
- Concordance of clinician and patient reported toxicity measures at each time point where both were assessed

For peer review only

 For peer review only - http://bmjopen.bmj.com/site/about/guidelines.xhtml

## 5. PATIENT SELECTION & ELIGIBILITY

### 5.1. Number of participants

The aim is to recruit 62 participants, 31 to each treatment allocation.

### 5.2. Source of participants

Participants will be recruited from approximately 10 participating sites in the UK. Patients will be approached about participation in HYBRID if they are considered at multi-disciplinary team meetings to be unfit for daily radical radiotherapy and fulfil the eligibility criteria.

### 5.3. Inclusion criteria

1. Written informed consent
2. Age ≥18 years
3. Histologically or cytologically confirmed bladder carcinoma
4. Bladder cancer staged T2-T4a N0 M0
5. Unsuitable for radical cystectomy or daily fractionated radiotherapy for any reason (including performance status, co-morbidity, patient refusal)
6. Expected survival >6 months
7. WHO performance status 0-3 (Appendix 1)
8. Willing to undergo post treatment cystoscopy
9. Able to attend for post treatment follow up

### 5.4. Exclusion criteria

1. Nodal or metastatic disease
2. Concurrent malignancy within 2 years of randomisation (not including non melanomatous skin carcinoma, previous non muscle invasive bladder tumours, NCCN low risk prostate cancer (T1/T2a, Gleason 6 PSA <10), in situ carcinoma of any site)
3. Previous pelvic radiotherapy
4. Urinary catheter in-situ
5. Any other contra-indication to radiotherapy (e.g. inflammatory bowel disease)

### 5.5. Lifestyle guidelines

It is highly unlikely that the patient population included in HYBRID will be at risk of pregnancy or fathering a child. However if this is a possibility for any individual patient, this should be discussed and the patient should be advised to use barrier protection and avoid conception for 12 months after treatment.

## 6. SCREENING

### 6.1. Screening log

All participating centres will be required to keep a detailed log of all patients with muscle invasive bladder cancer discussed at multi-disciplinary team meetings who are considered unsuitable for cystectomy or daily radiotherapy treatment. This log will capture the following information:

- Date patient identified
- Number of patients approached/accepting/declining participation/ineligible
- Screening outcome


For peer review only - http://bmjopen.bmj.com/site/about/guidelines.xhtml

- Trial ID (if applicable)
- Reasons for ineligibility / not approaching / declining as applicable

This information will be used to monitor recruitment activity. No patient identifiable data will be collected at this stage.

### 6.2. Procedure for obtaining informed consent

The Principal Investigator (or designated individual) must ensure that each trial patient is fully informed about the nature and objectives of the trial and possible risks associated with participation. Participants should be given the current REC approved HYBRID patient information sheet for their consideration. Patients should only be asked to consent to the study after they have had sufficient time to consider the trial, and the opportunity to ask any further questions.

No protocol required assessments should be conducted until the HYBRID consent form has been signed and dated by both the patient and the Investigator, unless they are performed routinely as part of standard patient care.

Patients who consent to HYBRID will be asked to consent to participate in the Patient Reported Outcomes (PRO) sub-study. Patients should be made aware that participation in the PRO sub-study is entirely voluntary. Refusal to participate in the PRO sub-study will not result in ineligibility to participate in the main clinical trial and will not impact the medical care received.

Confirmation of the patient's consent and the informed consent process must be documented in the patient's medical notes. A copy of the signed consent form should be provided to the patient and the original retained in the investigator site file, which must be available for verification by ICR-CTSU study staff.

### 6.3. Participation in other research

Patients who fulfil the eligibility criteria will be given the opportunity to participate in HYBRID even if they have participated in other research prior to recruitment.

HYBRID participants will not be permitted to participate in any trials whilst they are being treated within HYBRID or for 3 months afterwards.

Participation in other research will be considered on a case by case basis by the Trial Management Group.

## 7. RANDOMISATION

Patients must be randomised centrally by the trials unit (ICR-CTSU) before trial treatment can commence.

Patients should be randomised by telephoning ICR-CTSU on:

**020 8643 7150**

09.00-17.00 (UK time) Monday to Friday

Randomisation should take place ideally within 4 and within a maximum of 6 weeks prior to the planned start date of radiotherapy. An eligibility and randomisation checklist must be completed prior to randomisation.

The following information will be required at randomisation:

- Name of hospital, consultant and person randomising patient
- Confirmation that patient has given written informed consent for trial and for any sub-studies;
- Confirmation that patient is eligible for the trial by completion of the eligibility checklist
- Patient's full name, hospital number, date of birth, postcode and NHS/CHI number


For peer review only - http://bmjopen.bmj.com/site/about/guidelines.xhtml

The caller will be given the patient's unique randomisation number (Trial ID) and treatment allocation (see section 14.2).

ICR-CTSU will send written confirmation of trial entry to the data management contact at the recruiting centre.

# 8. TRIAL ASSESSMENTS

## 8.1. Pre-randomisation assessments

The following assessments should be conducted prior to randomisation:

- Histological confirmation of bladder cancer
- Radiological assessment of muscle invasive bladder cancer. Ideally within 4 weeks and within a maximum of 6 weeks prior to randomisation. To include a minimum of CT of abdomen and pelvis with chest x-ray (CT of chest, abdomen and pelvis; or MRI pelvis and CT chest and abdomen are also acceptable, according to local practice).

## 8.2. Pre-treatment assessments

The following assessments should be conducted within 14 days prior to the start of treatment:

- Assessment of baseline symptoms (CTCAE v. 4)
- Full blood count, urea and electrolytes
- Patient reported outcomes (IBDQ, KHQ and EQ5D)

## 8.3. On-treatment assessments

The following assessments should be conducted weekly (ideally prior to delivery of radiotherapy)

- Acute toxicity assessment (CTCAE v.4)

  At fractions 2, 4 and 6

- Full blood count, urea and electrolytes

  **At fraction 6:**

- Patient reported outcomes (IBDQ, KHQ and EQ5D)

## 8.4. Post radiotherapy assessments

### 8.4.1. 4 weeks from last radiotherapy fraction

- Acute toxicity assessment (CTCAE v.4)

### 8.4.2. 3 months from last radiotherapy fraction

- Acute toxicity assessment (CTCAE v.4)
- Cystoscopy under general anaesthetic, with tumour bed biopsy (if not possible, flexible cystoscopy with visual inspection of tumour bed and urine cytology. If flexible cystoscopy is not possible please contact ICR-CTSU for advice).
- Patient reported outcomes (IBDQ, KHQ and EQ5D)

### 8.4.3. 6 and 12 months (from last radiotherapy fraction)

- Late toxicity assessment (CTCAE v.4 and RTOG (Appendix 2))
- Flexible cystoscopy with visual inspection of tumour bed (if not possible, urine cytology and CT scan of pelvis)
- Patient reported outcomes at 6 months only (IBDQ, KHQ and EQ5D)


For peer review only - http://bmjopen.bmj.com/site/about/guidelines.xhtml

#### 8.4.4. 24 months (from last radiotherapy fraction)

- Late toxicity assessment (CTCAE v.4 and RTOG)
- Assessment of disease status, by clinical examination and flexible cystoscopy if possible.

#### 8.4.5. Annually thereafter

Patients will not be required to undergo any trial specific investigations; however data will be requested annually from standard follow up visits relating to:

- Assessment of disease status
- Survival

### 8.5. Procedure at disease progression/recurrence

Participants should be treated according to local clinical judgement at disease progression/recurrence.

### 8.6. Withdrawal from treatment or follow-up

Participants may withdraw from trial treatment at any time at their own request, or they may be withdrawn at the discretion of the Principal Investigator. Reasons for withdrawal may include:

- Disease progression
- Unacceptable toxicity
- Co-morbidities

Participants who discontinue treatment should continue to be followed up. If a patient withdraws from further follow-up a trial deviation form should be submitted to ICR-CTSU stating whether the patient has withdrawn consent for information to be sent to the ICR-CTSU or whether they simply no longer wish to attend trial follow up visits. In the very rare event that a patient requests that their data is removed from the study entirely, the implications of this should be discussed with the patient first to ensure that this is their intent and, if confirmed, ICR-CTSU should be notified in writing. If this request is received after results have been published the course of action will be agreed between the Sponsor and Independent Data Monitoring and Trial Steering Committees.

For peer review only


For peer review only - http://bmjopen.bmj.com/site/about/guidelines.xhtml

## 9. SCHEDULE OF ASSESSMENTS

| Visit/Assessment | Pre-randomisation | Up to 14 days pre-treatment | On treatment (before each fraction) | 4 weeks after completion of radiotherapy | 3 months after completion of radiotherapy | 6 months after completion of radiotherapy | 12 months after completion of radiotherapy | 24 months after completion of radiotherapy | Annually thereafter |
|---|---|---|---|---|---|---|---|---|---|
| Histological confirmation of bladder cancer | X | | | | | | | | |
| Radiological assessment of bladder cancer (minimum CT abdomen and pelvis and chest x-ray) | X[1] | | | | | | | | |
| Acute toxicity assessment (CTCAE v.4) | | X | X | X | X | | | | |
| Full blood count, urea and electrolytes | | X | X[2] | | | | | | |
| Patient reported outcomes questionnaire (IBDQ, KHQ and EQ5D) | | X | X[3] | | X | X | | | |
| Cystoscopy under general anaesthetic with tumour bed biopsy *(if not possible, flexible cystoscopy with visual inspection of tumour bed and urine cytology)* | | | | | X | | | | |
| Late toxicity assessment (CTCAE v.4 and RTOG) | | | | | | X | X | X | |
| Flexible cystoscopy with visual inspection of tumour bed *(if not possible, urine cytology and pelvic CT scan)* | | | | | | X | X | | |
| Assessment of disease status | | | | | | | | X | X |

Footnotes

1. Baseline radiological assessment should take place ideally within 4 weeks and within a maximum of 6 weeks prior to randomisation
2. Full blood count, urea and electrolytes prior to fractions 2, 4 and 6 only
3. PRO questionnaire at fraction 6 only



For peer review only - http://bmjopen.bmj.com/site/about/guidelines.xhtml

# 10. TREATMENT

Details of radiotherapy planning are given in the accompanying HYBRID radiotherapy planning and delivery guidelines, supplied by ICR-CTSU, which must be used as the primary source for radiotherapy planning within HYBRID.

## 10.1. Standard pre-trial treatment

All participants should have a transurethral resection of bladder tumour (if possible) or tumour biopsy prior to trial entry.

## 10.2. Treatment timelines

Radiotherapy should commence within 6 weeks following randomisation (to allow sufficient time for planning).

## 10.3. Radiotherapy planning and delivery

All participants will be planned to receive six 6Gy fractions of radiotherapy delivered weekly (total dose: 36Gy). Participants will be allocated at random to either standard or adaptive planning. Radiotherapy planning and outlining should be carried out in accordance with the guidelines in the current version of the radiotherapy planning and delivery guidelines, available in the HYBRID site investigator file and on request from ICR-CTSU (HYBRID-icrctsu@icr.ac.uk).

## 10.4. Treatment scheduling and gaps

Treatment can start on any day of the week and each fraction should be given on the same day of the week at weekly intervals +/- 2 day(s).

A gap of up to 9 (standard 7 days plus 2) days is acceptable in the event of machine service or breakdown. If the treatment machine is unavailable for more than 2 days, please contact the HYBRID trial manager.

## 10.5. Supportive care guidelines

In the event of patient catheterisation during the course of treatment it is expected that the participant will continue and complete radiotherapy in accordance with their allocated treatment group. As the bladder requires emptying prior to treatment delivery, the catheter must be on free flow in circumstances where there is a leg bag or voided in circumstances where there is a flip-valve.

Participants' symptoms should be managed according to local practice, although the following are suggestions for patient care:

Anaemia: Patients should be maintained by transfusion with haemoglobin above 11 grams. Iron deficiency should be treated with iron supplementation.

Dysuria/Frequency: Check for evidence of infection and treat if present with appropriate antibiotics, anticholinergics (eg oxybutynin, tolterodine), NSAIDs, analgesics.

Diarrhoea: Loperamide or opioid

Proctitis: steroid suppository +/- local anaesthetics (e.g. sheriproct, proctosedyl)

## 10.6. Concomitant therapy

All medication considered necessary for the patients' welfare and which is not expected to interfere with the evaluation of the treatment may be given at the discretion of the investigator.

Concomitant chemotherapy and/or Carbogen Nicotinamide are not considered standard of care for this patient population but may be used cautiously at investigators' discretion, however ICR-CTSU should be contacted in advance to gain approval from the Trial Management Group.



For peer review only - http://bmjopen.bmj.com/site/about/guidelines.xhtml

## 11. RADIOTHERAPY QUALITY ASSURANCE (QA)

A comprehensive QA programme for the HYBRID trial will be designed and implemented by the NCRI Radiotherapy Clinical Trials Quality Assurance (NCRI RTTQA) group. This will include pre-trial and on-trial components. Selection of appropriate treatment plans for the adaptive planning group will be independently monitored as part of the ongoing radiotherapy QA process. A member of the NCRI RTTQA group will visit all participating centres during the treatment delivery to the first patient receiving adaptive radiotherapy at each site. Subsequent plan selection will be subject to retrospective blind review by members of the NCRI RTTQA group, and the outcome of this will be fedback to centres in order to maintain and reinforce standard selection parameters across all participating sites.

### 11.1. Pre-trial quality assurance programme

The following will need to be completed by participating centres prior to site activation.

1. Facility questionnaire
2. Process document
3. Benchmark outlining case
4. Benchmark planning case
5. IGRT competency programme - on-line training package, practical workshop and independent competency check

### 11.2. On-trial quality assurance programme

1. On site independent plan selection review. This will be completed for the first adaptive radiotherapy participant at each investigator site by the NCRI RTTQA group prior to the site recruiting a subsequent participant.
2. Remote retrospective plan selection review for all adaptive radiotherapy patients
3. Plan and image data collection

## 12. SAFETY REPORTING

### 12.1. Adverse event (AE)

Any untoward medical occurrence in a patient or clinical trial subject administered a research procedure; events do not necessarily have a causal relationship with the procedure.

### 12.2. Serious adverse event (SAE)

An SAE is any untoward medical occurrence that occurs after the commencement of study treatment and:

- results in death;
- is life-threatening;
- requires hospitalisation or prolongation of existing hospitalisation;
- results in persistent or significant disability or incapacity;
- consists of a congenital anomaly or birth defect;
- is otherwise considered medically significant by the investigator; or
- is any non-genitourinary adverse event grade 3 or higher (CTCAE v4) occurring up to 3 months after completion of radiotherapy


For peer review only - http://bmjopen.bmj.com/site/about/guidelines.xhtml

Important adverse events that are not immediately life-threatening or do not result in death or hospitalisation, but may jeopardise the subject or require intervention to prevent one of the other outcomes listed in the definition above, may also be considered serious.

Progression of the indicated disease and death due to progression of the indicated disease are not considered SAEs.

Pregnancy or aid in the conception of a child whilst participating in a trial is not itself considered an SAE but should be followed up for congenital anomalies.

## 12.3. Serious Adverse Reaction (SAR)

A serious adverse reaction is an SAE that is suspected as having a causal relationship to the research procedure, as assessed by the investigator responsible for the care of the patient. A suspected causal relationship is defined as possibly, probably or definitely related (see definitions of causality table).

### 12.3.1. Definitions of causality

| Relationship | Description |
|---|---|
| Unrelated | There is no evidence of any causal relationship with the trial procedure |
| Unlikely | There is little evidence to suggest there is a causal relationship (e.g. the event did not occur within a reasonable time after the trial procedure. There is another reasonable explanation for the event (e.g. the patient's clinical condition, other concomitant treatment) |
| Possible | There is some evidence to suggest a causal relationship (e.g. because the event occurs within a reasonable time after the trial procedure. However, the influence of other factors may have contributed to the event (e.g. the patient's clinical condition, other concomitant treatments) |
| Probable | There is evidence to suggest a causal relationship, and the influence of other factors is unlikely |
| Definitely | There is clear evidence to suggest a causal relationship, and other possible contributing factors can be ruled out |
| Not assessable | There is insufficient or incomplete evidence to make a clinical judgement of the causal relationship. |

## 12.4. Related Unexpected SAE

A serious adverse reaction that is assessed by the Chief Investigator or nominative representative as unexpected.

## 12.5. Reporting Adverse Events to ICR-CTSU

Any toxicity, sign or symptom that occurs after randomisation should be considered an AE.

All AEs must be reported on the relevant toxicity, sign or symptom CRF.

The severity of AEs should be graded according to CTCAE v4 criteria. For each toxicity/sign/symptom, the highest grade observed since the last visit should be reported.

Whenever one or more toxicity/sign/symptom corresponds to a disease or a well-defined syndrome only the main disease/syndrome should be reported.


For peer review only - http://bmjopen.bmj.com/site/about/guidelines.xhtml

### 12.6. Reporting Serious Adverse Events to ICR-CTSU

Any SAE (except those listed below) that occurs from the start of radiotherapy and up to 30 days following the last day of radiotherapy must be reported.

**In addition, any non-genitourinary adverse event grade 3 or higher (CTCAE v.4) and occurring up to 3 months after completion of radiotherapy must be reported.**

All SAEs should be reported to ICR-CTSU within 24 hours of the Principal Investigator (or designated representative) becoming aware of the event, by completing the HYBRID SAE form and faxing to:

The ICR-CTSU safety desk
Fax no: **0208 722 4368**
For the attention of the HYBRID Trial team

As much information as possible, including the Principal Investigator's assessment of causality, must be reported to ICR-CTSU in the first instance. Additional follow up information should be reported as soon as it is available.

All SAE forms must be completed, signed and dated by the Principal Investigator or designated representative.

The Site SAE log should be completed and the SAE form filed in the Site Investigator File.

### 12.7. Serious Adverse Events exempt from expedited reporting

#### 12.7.1. Expected genitourinary events:

The following adverse events are exempt from expedited reporting if grade **≤3**:

- Haematuria
- Dysuria/frequency
- Bladder spasms or pain
- Urinary tract infection
- Urinary/clot retention

#### 12.7.2. Expected non-genitourinary events

The following adverse events are exempt from expedited reporting if grade **≤2**:

- Admission for transfusion secondary to bleeding from bladder tumour or anaemiaNausea/vomitingDiarrhoea

### 12.8. Abdominal painReview of Serious Adverse Events

The Chief Investigator (or designated representative) will assess all reported SAEs for causality and expectedness (NB. The Chief Investigator cannot down-grade the Principal Investigator's assessment of causality.)

Sites should respond as soon as possible to requests from the Chief Investigator or designated representative (via ICR-CTSU) for further information that may be required for final assessment of an SAE.


For peer review only - http://bmjopen.bmj.com/site/about/guidelines.xhtml

### 12.9. Expedited Reporting of Related Unexpected SAEs

If an SAE is identified as being related and unexpected by the Chief Investigator it will be reported by ICR-CTSU to the main REC within 15 days of being notified of the event.

The Principal Investigators at all actively recruiting sites will be informed of any related unexpected SAEs occurring within the trial at regular intervals.

### 12.10. Follow up of Serious Adverse Events

SAEs should be followed up until clinical recovery is complete or until the condition has stabilised. SAE outcomes should be reported to ICR-CTSU using the relevant section of the SAE form as soon as the Principal Investigator becomes aware of the outcome.

### 12.11. Annual reporting of safety considerations

Any safety concerns will be reported to the main REC by ICR-CTSU as part of the annual progress report at the end of the reporting year. This will be defined as the anniversary of the date when the study received a favourable opinion from the Main REC.

## 13. STATISTICAL CONSIDERATIONS

### 13.1. Statistical design and sample size justification

The sample size is based on the primary endpoint of acute non-genitourinary grade 3 or greater toxicity (toxicity being defined as adverse events related to study treatment). An A'Hern exact phase II design has been used to rule out an upper limit of toxicity for each planning method separately. Using data from the APPLY study it is expected that the acute non-genitourinary CTCAE grade 3+ rate will be 10% in patients receiving adaptive planning. The study has been designed to rule out a 30% upper limit of grade 3+ non-genitourinary toxicity within each planning method. With 80% power and 5% alpha (one-sided) this requires 28 patients in each group. If 5 or more patients within either planning method experience a non-GU grade 3+ toxicity then toxicity will be assumed to be too high. To be assessable for acute toxicity patients are required to have received at least 1 fraction of radiotherapy. If any patients stop treatment early for a reason clearly unrelated to toxicity the TSC will advise on evaluability. Recruitment will continue until there are 28 evaluable patients in each planning method group. A 10% non-evaluable rate has been accounted for in the target sample size of 62 patients. Recruitment beyond 62 patients, if more than 10% are non-evaluable, will be reviewed by the TSC.

There are a number of important secondary endpoints which the study will have sufficient statistical power to address. The overall toxicity of a 6Gy 6 fraction schedule will be assessed by combining the standard and adaptive planning groups. Using data from BC2001 and the APPLY study it is estimated that the any grade 3+ acute toxicity rate will be 25%. With 62 patients, there will be 80% power (5% alpha) to rule out an upper limit of 40% any grade 3+ acute toxicity with hypofractionated radiotherapy.

Local disease control will be assessed by combining the standard and adaptive planning groups to assess the effectiveness or otherwise of 36Gy/6f. The expected rate of local disease control at 3 months is 60%. If the true rate is 40% weekly hypofractionated RT would be judged ineffective. With 62 patients there is 87% power (5% one-sided alpha) to rule out less than 40% control rate, allowing for 25% non-evaluable patients at 3 months.

In pilot studies it is estimated that about 50% of treatments would benefit from adaption (either smaller or larger margins than standard). If >25% of all fractions treated or if >1fraction/patient require intervention, we anticipate clinical benefit for online correction. To determine a rate of less than 25% if the true rate is 50% with 86% power would require 29 patients to be treated in the adaptive planning group.


For peer review only - http://bmjopen.bmj.com/site/about/guidelines.xhtml

Another key secondary endpoint is the appropriate identification and correction of fractions requiring adaptive planning. The sample size provides sufficient power to investigate this question. If the true agreement between online and offline protocols is 85% and we wish to prove it is >75%, using a single-stage exact binomial phase II design, then for 90% power 139 fractions are required. If each patient receives 5 fractions on average, then 28 patients are needed. There is some allowance for the possibility that patients receive fewer than 5 fractions with 31 patients receiving adaptive planning (average treatment of 4.5 fractions each).

### 13.2. Treatment allocation

Participants will be randomised between standard and adaptive treatment delivery on a 1:1 basis.

Treatment allocation is by minimisation with a random element; balancing factors will be listed in the statistical analysis plan.

### 13.3. Primary endpoint definition

Acute non genitourinary toxicity will be assessed and reported using the CTCAE toxicity scale. Any grade 3 or greater treatment-related toxicities occurring within the first 3 months of radiotherapy completing will be included in assessment of the primary endpoint. Relatedness to study treatment will be subject to independent review blinded to planning method. If there are discrepancies between the PI, CI and independent assessment of relatedness, the independent assessment will take precedent for the purpose of enumeration of primary endpoint events. To be assessable for acute toxicity patients are required to have received at least 1 fraction of radiotherapy.

### 13.4. Secondary endpoint definitions

#### 13.4.1. Local disease control at 3 months

This will be assessed by general anaesthetic cystoscopy and with a tumour bed biopsy at 3 months. Patients will be classified as either having evidence of residual tumour at 3 months or not. Data will be presented for all patients combined, regardless of treatment allocation. Patients will only be included in the denominator if they were able to have a disease assessment at 3 months.

#### 13.4.2. Control rate of presenting symptoms

This will be assessed by looking at change in symptom scores from pre to post radiotherapy. Pre-radiotherapy symptoms will be recorded using the CTCAE toxicity scale and compared with CTCAE scores at 3 months from the completion of radiotherapy. The number of patients with post-radiotherapy scores equal to or lower than their baseline score will be used to calculate the control rate of presenting symptoms. Data will be presented separately for the two randomisation groups

#### 13.4.3. Patient reported outcomes (PRO)

This will be assessed using the modified Inflammatory Bowel Disease Questionnaire (IBDQ), the King's Health Questionnaire and the EQ5D. PRO will be assessed pre-radiotherapy, at fraction 6 and, 3 and 6 months from completion of radiotherapy. The IBDQ bowel-related symptoms at 3 months from completion of radiotherapy are of primary interest. Data will be presented separately for the two randomisation groups.

#### 13.4.4. Late toxicity

This will be assessed using CTCAE and RTOG scoring criteria at 6, 12 and 24 months from completion of radiotherapy. Data will be presented separately for the two randomisation groups.

#### 13.4.5. Time to local disease progression

This will be measured from randomisation to the first occurrence of local disease as identified by cystoscopy or cytology and CT.


For peer review only - http://bmjopen.bmj.com/site/about/guidelines.xhtml

#### 13.4.6. Overall survival

This will include deaths from any cause and time will be measured from randomisation

#### 13.4.7. Proportion of fractions benefiting from adaptive planning

This will be assessed by the number of small or large plans being selected rather than the medium plan for patients in the adaptive planning group. The denominator will be the total number of fractions received in the adaptive planning group.

#### 13.4.8. Appropriate identification and correction of fractions requiring adaptive planning

This will require an independent reviewer to select an appropriate plan for each fraction for each patient. The concordance between the actual and independent reviewer plan selection will be presented.

### 13.5. Exploratory endpoints

Dose Volume Histogram analysis of CTV coverage using anisotropic margins will be assessed.

The level of agreement between clinician reported toxicity and PRO will also be explored at each time point where both assessments are used.

### 13.6. Analysis plan

The numbers and proportions of patients with acute non-genitourinary CTCAE v4 toxicity grade 3 or greater in each planning method will be presented together with 95% one-sided exact confidence intervals (the 90% two-sided confidence interval will also be presented). The primary analysis population will be patients who received 1 fraction of radiotherapy. Sensitivity analyses will also be conducted including all randomised patients in the denominator and also only patients who received at least 3 fractions of radiotherapy.

Acute toxicity figures will also be presented by planning method for any grade 3 or greater non-genitourinary adverse event (i.e. including treatment related and not), and for any genitourinary grade 3 or greater adverse event (treatment related and all events) and any grade 3 or greater adverse event (treatment related and all events) for the two planning methods combined. The distribution of non-genitourinary and genitourinary adverse events/toxicity will be presented as numbers and proportion at each time point they are assessed.

The local control rate at 3 months will be presented for all patients (standard and adaptive combined) together with a 95% confidence interval (CI). The local control rate will be presented as a proportion with patients only included in the denominator if they were able to have an assessment at 3 months. The principal analysis will be based on all data reported on the 3 month assessment form will be used with no restrictions on time windows.

Late toxicity will be summarised by frequencies and proportions at each time point by treatment group. Kaplan-Meier methods may be used to present time-to-event data e.g. time to first occurrence of a grade 2 or greater event.

Kaplan-Meier methods will be used to analyse time to local disease progression and overall survival with data presented for randomised groups combined. Data will not be presented by treatment group as there is insufficient statistical power to detect clinically meaningful differences and this is not the aim of this phase II study. Time will be measured from randomisation and patients with no event will be censored on the data of last clinical assessment.

The proportion of fractions requiring clinical intervention will be calculated as the number out of all fractions treated, and reported with an exact binomial confidence interval.


For peer review only - http://bmjopen.bmj.com/site/about/guidelines.xhtml

PRO scores will be generated by combining individual items to produce subscale scores for each domain of the questionnaires using standard algorithms. The frequency and percentages at each time point will be presented for each PRO questionnaire. Data will be presented separately for each randomised group.

Pre-planned exploratory sub-group analyses will be conducted to investigate potential differences in toxicity in the following sub-groups, if there are sufficient numbers within each subgroup:

- Histology: TCC vs any other tumour types
- Staging: T2 vs T3/T4
- Performance status: 0/1 vs 2/3

Further details of analysis methods will be specified in a Statistical Analysis Plan in accordance with ICR-CTSU Standard Operating Procedures.

### 13.7. Interim analyses and stopping rules

This is a phase II trial and there will be no formal early stopping rules for efficacy or toxicity. As per the single stage design, if 5 or more patients report treatment related non-genitourinary grade 3 or greater acute toxicities in one planning group then randomisation will be suspended and the Independent Data Monitoring Committee (IDMC) will review the data and be asked to advise on continuation of recruitment to both planning groups.

The IDMC will closely monitor accumulating safety and efficacy data at regular intervals (to be determined by recruitment rates but likely to be 6-12 monthly) and will advise if there are any safety signals. An initial safety review will take place when 3 month data are available for 5 patients (who have received at least 3 fractions of RT) in each group.

## 14. TRIAL MANAGEMENT

### 14.1. Trial Management Group (TMG)

A Trial Management Group (TMG) will be set up and will include the Chief Investigator, ICR-CTSU Scientific Lead, Co-investigators and identified collaborators, the Trial Statistician and Trial Manager. Principal Investigators and key study personnel will be invited to join the TMG as appropriate to ensure representation from a range of sites and professional groups. Where possible, membership will include a lay/consumer representative. The TMG will meet at regular intervals, and at least annually. Notwithstanding the legal obligations of the sponsor and Chief Investigator, the TMG have operational responsibility for the conduct of the trial. The Committee's terms of reference, roles and responsibilities will be defined in a charter issued by ICR-CTSU.

### 14.2. Trial Steering Committee (TSC)

The HYBRID trial will be overseen by the ICR-CTSU Bladder Trials Steering Committee (TSC) which includes an independent Chairman (not involved directly in the trial other than as a member of the TSC) and not less than two other independent members. The TSC will meet annually. The TSC will provide expert independent oversight of the trial on behalf of the sponsor and funder. The Committee's terms of reference, roles and responsibilities will be defined in charter issued by ICR-CTSU.

### 14.3. Independent Data Monitoring Committee (IDMC)

An IDMC will be instigated to monitor the progress of the trial and will include at least three independent members, one of whom will be a medical statistician. The Committee's terms of reference, roles and responsibilities will be defined in a charter issued by ICR-CTSU. In the absence of a separate independent assessor(s), a clinical member(s) of the IDMC will perform the


For peer review only - http://bmjopen.bmj.com/site/about/guidelines.xhtml

independent review of relatedness to study treatment of grade 3 or greater acute non-genitourinary adverse events.

The IDMC will meet in confidence at regular intervals, and at least annually. A summary of findings and any recommendations will be produced following each meeting. This summary will be submitted to the TMG and TSC, and if required, the main REC.

The IDMC reserve the right to release any data on outcome or side-effects through the TSC to the TMG (and if appropriate to participants) if it determines at any stage that the combined evidence from this and other studies justifies it.

## 15. RESEARCH GOVERNANCE

### 15.1. Sponsor responsibilities

The sponsor of this clinical trial is the Institute of Cancer Research (ICR).

Responsibilities of participating sites are defined in an agreement between the individual participating site and the Sponsor

## 16. TRIAL ADMINISTRATION & LOGISTICS

### 16.1. Site activation

Before activating the trial, participating sites are required to sign an agreement accepting responsibility for all trial activity which takes place within their site.

Sites may commence recruitment once the site agreement has been signed by all required signatories, the required trial documentation is in place (as specified by ICR-CTSU) and a site initiation (visit or teleconference) has taken place. Site initiation visits will be conducted at sites where the Principal Investigator has requested one or where ICR-CTSU deems it is appropriate.

### 16.2. Investigator training

Each centre will complete the comprehensive pre-trial section of the quality assurance programme prior to commencing recruitment, as detailed in section 12. In addition to this, prior to trial initiation, a practical workshop will be held to educate Principal Investigators, radiographers and physicists in adaptive radiotherapy techniques. The quality assurance programme will continue throughout the trial, with investigator training as required.

### 16.3. Data acquisition

Electronic (e) Case Report Forms (CRF) will be used for the collection of trial data. ICR-CTSU will provide guidance to sites to aid the completion of the eCRFs. The Trial Management Group reserves the right to amend or add to the eCRF template as appropriate. Such changes do not constitute a protocol amendment, and revised or additional forms should be used by sites in accordance with the guidelines provided by ICR-CTSU.

### 16.4. Central data monitoring

Once data has been entered on the eCRF by the site personnel, ICR-CTSU will review it for compliance with the protocol, and for inconsistent or missing data. Should any missing data or data anomalies be found, queries will be raised for resolution by the site.

Any systematic inconsistencies identified through central data monitoring may trigger an on-site monitoring visit.


For peer review only - http://bmjopen.bmj.com/site/about/guidelines.xhtml

### 16.5. On-site monitoring

If a monitoring visit is required, ICR-CTSU will contact the site to arrange the visit. Once a date has been confirmed, the site should ensure that full patient notes of participants selected for source data verification are available for monitoring.

ICR-CTSU staff conducting on-site monitoring will review essential documentation and carry out source data verification to confirm compliance with the clinical trial agreement and trial protocol. If any problems are detected during the course of the monitoring visit, ICR-CTSU will work with the Principal Investigator or delegated individual to resolve issues and determine appropriate action.

### 16.6. Completion of the study and definition of study end date

The study end date is deemed to be the date of last data capture.

### 16.7. Archiving

Essential trial documents should be retained according to local policy and for a sufficient period for possible inspection by the regulatory authorities (at least 5 years after the date of last data capture). Documents should be securely stored and access restricted to authorised personnel.

## 17. PATIENT PROTECTION AND ETHICAL CONSIDERATIONS

### 17.1. Trial approvals

This trial has been formally assessed for risk by ICR-CTSU.

The trial has received ethical approval from a research ethics committee for multi-centre trials and global R&D approval via the NIHR Coordinated System for gaining NHS Permission. Before entering patients, the Principal Investigator at each site is responsible for submitting Site Specific Information and gaining local Research and Development approval of this protocol.

### 17.2. Trial conduct

This trial will be conducted in accordance with the Research Governance Framework for Health and Social Care and the principles of GCP.

### 17.3. Informed consent

Patients should be asked to sign the current main REC approved HYBRID consent form at trial entry after receiving both verbal and written information about the trial, having been given sufficient time to consider this information. All consent forms must be countersigned by the Principal Investigator or a designated individual. A signature log of delegated responsibilities, listing the designated individuals and the circumstances under which they may countersign consent forms, must be maintained at the participating site. This log, together with original copies of all signed patient consent forms, should be retained in the Site Investigator File and must be available for inspection. The current main REC approved HYBRID patient information sheets should be provided in addition to any standard patient information sheets that are provided by the site and used in routine practice.

### 17.4. Patient confidentiality

Patients will be asked to consent to their full name being collected at registration in addition to their date of birth, hospital number, postcode and NHS number or equivalent to allow linkage with routinely collected NHS data.

Each investigator should keep a separate log of all participants' Trial IDs, names, addresses and hospital numbers. The investigator must retain trial documents (e.g. participants' written consent forms) in strict confidence. The investigator must ensure the participants' confidentiality is maintained at all times.

For peer review only


For peer review only - http://bmjopen.bmj.com/site/about/guidelines.xhtml

Representatives of the sponsor, ICR-CTSU, other coinvestigators, members of the hospital R&D team and regulatory authorities may require access to participants' notes for quality assurance and audit purposes. ICR-CTSU will maintain the confidentiality of participants at all times and will not reproduce or disclose any information by which participants could be identified.

### 17.5. Data Protection Act (DPA)

ICR-CTSU will comply with all aspects of the DPA 1998. Any requests from participants for access to their data held at ICR-CTSU will be referred to the Data Protection Officer at the ICR.

### 17.6. Liability

Indemnity for participating hospitals is provided by the usual NHS indemnity arrangements. Inclusion of private patients will be subject to the site ensuring appropriate insurance and indemnity arrangements are in place.

## 18. FINANCIAL MATTERS

This trial is investigator designed and led and has been approved by the Clinical Trials Advisory & Awards Committee (CTAAC) of Cancer Research UK.

ICR has received funding from Cancer Research UK for the central coordination of the trial. In the UK, the trial meets the criteria for R&D support as outlined in the Statement of Partnership on Non-Commercial R&D in the NHS in England. The trial is part of the National Institute for Health Research (NIHR) portfolio. Research Network resources should therefore be made available for the trial to cover UK specific research costs.

## 19. PUBLICATION POLICY

The main trial results will be published in a peer-reviewed journal, on behalf of all collaborators. The manuscript will be prepared by a writing group, consisting of members of the TMG and selected participating clinicians. All participating clinicians will be acknowledged in the publication.

Any presentations and publications relating to the trial must be authorised by the TMG. Authorship of any secondary publications e.g. those relating to sub-studies, will reflect the intellectual and time input into these studies.

No investigator may present or attempt to publish data relating to the HYBRID trial without prior permission from the TMG.

## 20. ASSOCIATED STUDIES

### 20.1. Patient reported outcome measures study

Patient reported outcomes will be a secondary endpoint in the main trial and will be analysed as described in the statistical analysis plan.

Further details are provided in Appendix 3.

For peer review only



For peer review only - http://bmjopen.bmj.com/site/about/guidelines.xhtml

## 21. REFERENCES

1 UK CR. Bladder cancer; 2013 Jan 2013.
2 Rodel C. Combined-Modality Treatment and Selective Organ Preservation in Invasive Bladder Cancer: Long-Term Results. Journal of Clinical Oncology. 2002;20(14):3061-71.
3 NICE. Guidance on Cancer Services Improving Outcomes in Urological Cancers The Manual. online: NICE; 2002.
4 Kotwal S, Choudhury A, Johnston C, Paul AB, Whelan P, Kiltie AE. Similar treatment outcomes for radical cystectomy and radical radiotherapy in invasive bladder cancer treated at a United Kingdom specialist treatment center. Int J Radiat Oncol Biol Phys. 2008 Feb 1;70(2):456-63.
5 Chahal R, Sundaram SK, Iddenden R, Forman DF, Weston PM, Harrison SC. A study of the morbidity, mortality and long-term survival following radical cystectomy and radical radiotherapy in the treatment of invasive bladder cancer in Yorkshire. Eur Urol. 2003 Mar;43(3):246-57.
6 NCIN. Cancer Incidence and Mortality By Cancer Network, UK, 2005. 2010.
7 Dunst J, Diestelhorst A, Kuhn R, Muller AC, Scholz HJ, Fornara P. Organ-sparing treatment in muscle-invasive bladder cancer. Strahlenther Onkol. 2005 Oct;181(10):632-7.
8 Shipley WU, Kaufman DS, Zehr E, et al. Selective bladder preservation by combined modality protocol treatment: long-term outcomes of 190 patients with invasive bladder cancer. Urology. 2002 Jul;60(1):62-7; discussion 7-8.
9 Huddart R, Norman A, Shahidi M, Dearnaley D, Horwich A. The effect of reducing the high dose volume on toxicity of radiotherapy in the treatment of bladder cancer. British Journal of Cancer. 1999;80(Supplement 2):A3.10.
10 Huddart RA, Hall E, Lewis R, Birtle A. Life and death of spare (selective bladder preservation against radical excision): reflections on why the spare trial closed. BJU Int. 2010 Sep;106(6):753-5.
11 Duchesne GM, Bolger JJ, Griffiths GO, et al. A randomized trial of hypofractionated schedules of palliative radiotherapy in the management of bladder carcinoma: results of medical research council trial BA09. Int J Radiat Oncol Biol Phys. 2000 May 1;47(2):379-88.
12 Jose CC, Price A, Norman A, et al. Hypofractionated radiotherapy for patients with carcinoma of the bladder. Clin Oncol (R Coll Radiol). 1999;11(5):330-3.
13 McLaren DB, Morrey D, Mason MD. Hypofractionated radiotherapy for muscle invasive bladder cancer in the elderly. Radiother Oncol. 1997 May;43(2):171-4.
14 Rostom AY, Tahir S, Gershuny AR, Kandil A, Folkes A, White WF. Once weekly irradiation for carcinoma of the bladder. Int J Radiat Oncol Biol Phys. 1996 May 1;35(2):289-92.
15 Fokdal L, Honore H, Hoyer M, Meldgaard P, Fode K, von der Maase H. Impact of changes in bladder and rectal filling volume on organ motion and dose distribution of the bladder in radiotherapy for urinary bladder cancer. Int J Radiat Oncol Biol Phys. 2004 Jun 1;59(2):436-44.
16 Lotz HT, Pos FJ, Hulshof MC, et al. Tumor motion and deformation during external radiotherapy of bladder cancer. Int J Radiat Oncol Biol Phys. 2006 Apr 1;64(5):1551-8.
17 Meijer GJ, Rasch C, Remeijer P, Lebesque JV. Three-dimensional analysis of delineation errors, setup errors, and organ motion during radiotherapy of bladder cancer. Int J Radiat Oncol Biol Phys. 2003 Apr 1;55(5):1277-87.
18 Muren LP, Smaaland R, Dahl O. Organ motion, set-up variation and treatment margins in radical radiotherapy of urinary bladder cancer. Radiother Oncol. 2003 Dec;69(3):291-304.
19 Sur RK, Clinkard J, Jones WG, et al. Changes in target volume during radiotherapy treatment of invasive bladder carcinoma. Clin Oncol (R Coll Radiol). 1993;5(1):30-3.
20 Turner SL, Swindell R, Bowl N, et al. Bladder movement during radiation therapy for bladder cancer: implications for treatment planning. Int J Radiat Oncol Biol Phys. 1997 Sep 1;39(2):355-60.
21 Pos F, Remeijer P. Adaptive management of bladder cancer radiotherapy. Semin Radiat Oncol. 2010 Apr;20(2):116-20.
22 Lalondrelle S, Huddart R. Improving radiotherapy for bladder cancer: an opportunity to integrate new technologies. Clin Oncol (R Coll Radiol). 2009 Jun;21(5):380-4.


For peer review only - http://bmjopen.bmj.com/site/about/guidelines.xhtml

23 Lalondrelle S, Huddart R, Warren-Oseni K, et al. Adaptive-predictive organ localization using cone-beam computed tomography for improved accuracy in external beam radiotherapy for bladder cancer. Int J Radiat Oncol Biol Phys. 2011 Mar 1;79(3):705-12.
24 National Radiotherapy Implementation Group Report Image Guided Radiotherapy (IGRT) Guidance for implementation and use.; 2012.
25 McDonald F, Lallondrelle S, Taylor H, et al. Clinical implementation of adaptive hypo-fractionated bladder radiotherapy for improvement in normal tissue irradiation. Submitted for publication. 2013.
26 McDonald F, Lalondrelle S, Taylor H, et al. Adaptive-predictive organ localisation [A-POLO] in the clinic: Updated results in hypo-fractionated bladder radiotherapy GU ASCO; 2011; 2011.
27 Andreyev HJ, Wotherspoon A, Denham JW, Hauer-Jensen M. Defining pelvic-radiation disease for the survivorship era. Lancet Oncol. Apr;11(4):310-2.
28 Guyatt G, Mitchell A, Irvine EJ, et al. A new measure of health status for clinical trials in inflammatory bowel disease. Gastroenterology. 1989 Mar;96(3):804-10.
29 Olopade FA, Norman A, Blake P, et al. A modified Inflammatory Bowel Disease questionnaire and the Vaizey Incontinence questionnaire are simple ways to identify patients with significant gastrointestinal symptoms after pelvic radiotherapy. Br J Cancer. 2005 May 9;92(9):1663-70.
30 Wedlake LJ, Thomas K, Lalji A, et al. Predicting Late Effects of Pelvic Radiotherapy: Is There a Better Approach? Int J Radiat Oncol Biol Phys. 2010 Mar 16:March 16.
31 Reese P, Pleil A, Okano G, Kelleher C. Multinational study of reliability and validity of the King's Health Questionnaire in patients with overactive bladder. Qual Life Res. 2003 2003/06/01;12(4):427-42.
32 Brooks R. EuroQol: the current state of play. Health policy (Amsterdam, Netherlands). 1996 Jul;37(1):53-72.

For peer review only


For peer review only - http://bmjopen.bmj.com/site/about/guidelines.xhtml

### A1. WHO performance status

| Grade | Performance Status |
|---|---|
| 0 | Able to carry out all normal activity without restriction. |
| 1 | Restricted in physically strenuous activity but ambulatory and able to carry out light work. |
| 2 | Ambulatory and capable of all self-care but unable to carry out any work; up and about more than 50% of waking hours. |
| 3 | Capable of only limited self-care; confined to bed or chair more than 50% of waking hours. |
| 4 | Completely disabled; cannot carry on any self-care; totally confined to bed or chair. |

For peer review only

### A2. RTOG/EORTC late radiation morbidity scoring schema

| 0 | 1 | 2 | 3 | 4 | 5 |
|---|---|---|---|---|---|
| BLADDER | | | | | |
| None | Slight epithelial atrophy<br>Minor telangiectasia (microscopic haematuria) | Moderate frequency<br>Generalized telangiectasia<br>Intermittent macroscopic haematuria | Severe frequency and dysuria<br>Severe generalized telangiectasia (often with petechiae)<br>Frequent haematuria<br>Reduction in bladder capacity (<150 cc) | Necrosis/ Contracted bladder (capacity <100 cc)<br>Severe haemorrhagic cystitis | Death due to toxicity |
| SMALL/LARGE INTESTINE | | | | | |
| None | Mild diarrhoea<br>Mild cramping<br>Bowel movement 5 times daily<br>Slight rectal discharge or bleeding | Moderate diarrhoea and colic<br>Bowel movement >5 times daily<br>Excessive rectal mucus or intermittent bleeding | Obstruction or bleeding requiring surgery | Necrosis/ Perforation Fistula | Death due to toxicity |


For peer review only - http://bmjopen.bmj.com/site/about/guidelines.xhtml

## A3. PATIENT REPORTED OUTCOMES STUDY

### A3.1 Background

The primary endpoint of HYBRID is clinician reported acute non-genitourinary toxicity within 3 months from completion of radiotherapy.

Patient reported outcomes (PRO) are a key secondary endpoint within HYBRID. The aim will be to collect detailed information about the impact of hypofractionated bladder radiotherapy on participants' daily lives, with a focus on side effects being experienced but also including a measure of general wellbeing.

The objective of the PRO sub-study within HYBRID is to compare the impact of adaptive planned hypofractionated radiotherapy on side effects as reported by the participants. This will help to support any differences in toxicity established within the primary endpoint of clinician reported toxicity. In addition, PRO data will be compared with clinician reported toxicity to give an indication of the concordance of the two measures.

### A3.2 Hypothesis

It is hypothesised that participants in the adaptive planning group will report fewer severe non-genitourinary toxicities than those in the standard planning group.

### A3.3 Quality of life measures

Patient reported outcomes will be measured using the modified Inflammatory Bowel Disease Questionnaire (IBDQ), King's Health Questionnaire (KHQ) and EQ-5D.

Lower gastrointestinal symptoms caused by radiation induced toxicity are similar to those in common bowel disorders unrelated to cancer therapies (27). The modified IBDQ (28) is the preferred patient reported measurement tools used by gastroenterologists specialising in pelvic radiotherapy-related side-effects (29). The modified IBDQ is a 32-item questionnaire consisting of four dimensions: bowel-related symptoms, systematic function (e.g. fatigue, sleep pattern), social function (e.g. ability to attend work and social events) and emotional status (e.g. anger, depression, irritability). The bowel subset of the modified IBDQ, the 'IBDQ-B' questionnaire has been used previously in a specialist service evaluating radiotherapy-related gastrointestinal side-effects. These patient reported questionnaires are easy to complete and are a sensitive indicator of radiotherapy toxicity (30).

Urinary side-effects experienced by participants will be captured using the KHQ, which has been validated for use in patients with overactive bladder (31) and captures details of the severity of symptoms and the impact of urinary incontinence on day to day living.

Participants will also be asked to complete the EQ5D questionnaire, a brief standardised instrument which provides a simple descriptive profile of health status (32).

### A3.4 Study design

Patients are eligible for the PRO study if they fulfil the HYBRID eligibility criteria. Participants will be asked in the patient information sheet to consent to regular completion of PRO questionnaires. Patients who decline to take part in the HYBRID PRO study will remain eligible for the main trial. PRO is a secondary endpoint in the main trial and the primary timepoint of interest is 3 months after completion of radiotherapy.


For peer review only - http://bmjopen.bmj.com/site/about/guidelines.xhtml

### A3.5 Timing of data collection

Participants will be asked to complete a questionnaire in clinic within two weeks prior to the start of treatment. Further questionnaires will be completed in clinic, at fraction six of treatment delivery and 3 and 6 months from the end of treatment.

### A3.6 Compliance

Missing data may hamper interpretation of PRO. Missing data may arise because participants do not complete the questionnaires at the appropriate time (unit non-response), or because patients may miss questions within the questionnaires (item non-response). In a population of patients with low performance status such as those included in HYBRID, there is potential for non-response and informative censoring (with data not missing at random). During the study, compliance with PRO questionnaire completion will be monitored by the trial oversight committees.

### A3.7 Statistical considerations

Patient reported outcome analyses will be used to supplement results of clinician assessed treatment toxicity, therefore a formal sample size calculation has not been performed. An analysis plan will be developed in consultation with the TMG with key endpoints identified from each questionnaire. Standard algorithms will be used to derive scores and handle missing data in quality of life questionnaires. Quality of life data will be presented at individual time-points and analyses to account for the longitudinal nature of the data may be used.

For peer review only


For peer review only - http://bmjopen.bmj.com/site/about/guidelines.xhtml

## A4. GLOSSARY

| | |
|---|---|
| AE | Adverse Event |
| APPLY | Adaptive predictive planning for hypofractionated bladder radiotherapy |
| CBCT | Cone beam CT |
| CI | Chief Investigator |
| CI | Confidence interval |
| CIS | Carcinoma in Situ |
| CRF | Case Report Form |
| CT | Computed tomography |
| CTCAE | Common Terminology Criteria for Adverse Events |
| CTV | Clinical target volume |
| DCF | Data Capture Form |
| DVH | Dose Volume Histogram |
| EORTC | European Organisation for Research and Treatment of Cancer |
| f | Fraction |
| FBC | Full Blood Count |
| GI | Gastrointestinal |
| GTV | Gross tumour volume |
| GU | Genitourinary |
| Gy | Gray |
| HR | Hazard Ratio |
| IBDQ | Inflammatory Bowel Disease Questionnaire |
| ICR | The Institute of Cancer Research |
| ICR-CTSU | The Institute of Cancer Research Clinical Trials and Statistics Unit |
| IDMC | Independent Data Monitoring Committee |
| IGRT | Image guided radiotherapy |
| KHQ | King's Health Questionnaire |
| MDT | Multi-disciplinary team |
| MIBC | Muscle invasive bladder cancer |
| MRI | Magnetic resonance imaging |
| NCRI | National Cancer Research Institute |
| NCRI RTTQA | NCRI Radiotherapy Clinical Trials Quality Assurance group |
| NICE | National Institute for Health and Clinical Excellence |
| NSAID | Non-steroidal anti-inflammatory drug |
| PI | Principal Investigator |
| PIS | Patient Information Sheet |
| PRO | Patient Reported Outcomes |
| PTV | Planning target volume |
| QA | Quality assurance |
| R&D | Research and Development |
| REC | Research Ethics Committee |
| RMH | Royal Marsden Hospital |
| RT | Radiotherapy |
| RTOG | Radiation Therapy Oncology Group |
| SAE | Serious Adverse Event |
| SAR | Serious Adverse Reaction |
| TMG | Trial Management Group |
| TSC | Trial Steering Committee |
| WHO | World Health Organisation |


For peer review only - http://bmjopen.bmj.com/site/about/guidelines.xhtml

For peer review only

This protocol is a controlled document and should not be copied, distributed or reproduced without the written permission of the ICR-CTSU

STANDARD PROTOCOL ITEMS: RECOMMENDATIONS FOR INTERVENTIONAL TRIALS

SPIRIT 2013 Checklist: Recommended items to address in a clinical trial protocol and related documents*

| Section/item | Item No | Description | Addressed on page number |
|---|---|---|---|
| **Administrative information** | | | |
| Title | 1 | Descriptive title identifying the study design, population, interventions, and, if applicable, trial acronym | p1 manuscript (p1 protocol) |
| Trial registration | 2a | Trial identifier and registry name. If not yet registered, name of intended registry | p7 manuscript (p1 protocol) |
| | 2b | All items from the World Health Organization Trial Registration Data Set | n/a |
| Protocol version | 3 | Date and version identifier | V 3.1 dated 13/8/2015 |
| Funding | 4 | Sources and types of financial, material, and other support | p21 manuscript (p21 protocol) |
| Roles and responsibilities | 5a | Names, affiliations, and roles of protocol contributors | p1 manuscript |
| | 5b | Name and contact information for the trial sponsor | p1 manuscript (p1 protocol) |
| | 5c | Role of study sponsor and funders, if any, in study design; collection, management, analysis, and interpretation of data; writing of the report; and the decision to submit the report for publication, including whether they will have ultimate authority over any of these activities | p26-28, 30 protocol |

For peer review only

| | | | |
|---|---|---|---|
| | 5d | Composition, roles, and responsibilities of the coordinating centre, steering committee, endpoint adjudication committee, data management team, and other individuals or groups overseeing the trial, if applicable (see Item 21a for data monitoring committee) | p15 manuscript (p27-29 protocol) |
| **Introduction** | | | |
| Background and rationale | 6a | Description of research question and justification for undertaking the trial, including summary of relevant studies (published and unpublished) examining benefits and harms for each intervention | p5-6 manuscript (p10-12 protocol) |
| | 6b | Explanation for choice of comparators | p11 protocol |
| Objectives | 7 | Specific objectives or hypotheses | p5-6 manuscript (p13 protocol) |
| Trial design | 8 | Description of trial design including type of trial (eg, parallel group, crossover, factorial, single group), allocation ratio, and framework (eg, superiority, equivalence, noninferiority, exploratory) | p7 manuscript (p13 protocol) |
| **Methods: Participants, interventions, and outcomes** | | | |
| Study setting | 9 | Description of study settings (eg, community clinic, academic hospital) and list of countries where data will be collected. Reference to where list of study sites can be obtained | p7 manuscript (p15 protocol) |
| Eligibility criteria | 10 | Inclusion and exclusion criteria for participants. If applicable, eligibility criteria for study centres and individuals who will perform the interventions (eg, surgeons, psychotherapists) | p7 manuscript (p15 protocol) |
| Interventions | 11a | Interventions for each group with sufficient detail to allow replication, including how and when they will be administered | p7-12 manuscript (p17-20 p15 protocol and additional radiotherapy planning and delivery protocol) |



For peer review only - http://bmjopen.bmj.com/site/about/guidelines.xhtml

| | | | |
|---|---|---|---|
| | 11b | Criteria for discontinuing or modifying allocated interventions for a given trial participant (eg, drug dose change in response to harms, participant request, or improving/worsening disease) | p15 manuscript (p27 protocol) |
| | 11c | Strategies to improve adherence to intervention protocols, and any procedures for monitoring adherence (eg, drug tablet return, laboratory tests) | p12-14 manuscript (p28-29 protocol and additional radiotherapy planning and delivery protocol) |
| | 11d | Relevant concomitant care and interventions that are permitted or prohibited during the trial | p27 protocol |
| Outcomes | 12 | Primary, secondary, and other outcomes, including the specific measurement variable (eg, systolic blood pressure), analysis metric (eg, change from baseline, final value, time to event), method of aggregation (eg, median, proportion), and time point for each outcome. Explanation of the clinical relevance of chosen efficacy and harm outcomes is strongly recommended | p7 manuscript (p14 protocol) |
| Participant timeline | 13 | Time schedule of enrolment, interventions (including any run-ins and washouts), assessments, and visits for participants. A schematic diagram is highly recommended (see Figure) | Table 1 manuscript (Table p19 of protocol) |
| Sample size | 14 | Estimated number of participants needed to achieve study objectives and how it was determined, including clinical and statistical assumptions supporting any sample size calculations | p14 manuscript (p24 protocol) |
| Recruitment | 15 | Strategies for achieving adequate participant enrolment to reach target sample size | p15-16 protocol |

**Methods: Assignment of interventions (for controlled trials)**

Allocation:

| | | | |
|---|---|---|---|
| Sequence generation | 16a | Method of generating the allocation sequence (eg, computer-generated random numbers), and list of any factors for stratification. To reduce predictability of a random sequence, details of any planned restriction (eg, blocking) should be provided in a separate document that is unavailable to those who enrol participants or assign interventions | p16, 25 protocol |

| | | | |
|---|---|---|---|
| Allocation concealment mechanism | 16b | Mechanism of implementing the allocation sequence (eg, central telephone; sequentially numbered, opaque, sealed envelopes), describing any steps to conceal the sequence until interventions are assigned | p16, 25 protocol |
| Implementation | 16c | Who will generate the allocation sequence, who will enrol participants, and who will assign participants to interventions | p16, 25 protocol |
| Blinding (masking) | 17a | Who will be blinded after assignment to interventions (eg, trial participants, care providers, outcome assessors, data analysts), and how | n/a |
| | 17b | If blinded, circumstances under which unblinding is permissible, and procedure for revealing a participant's allocated intervention during the trial | n/a |
| **Methods: Data collection, management, and analysis** | | | |
| Data collection methods | 18a | Plans for assessment and collection of outcome, baseline, and other trial data, including any related processes to promote data quality (eg, duplicate measurements, training of assessors) and a description of study instruments (eg, questionnaires, laboratory tests) along with their reliability and validity, if known. Reference to where data collection forms can be found, if not in the protocol | p28 protocol |
| | 18b | Plans to promote participant retention and complete follow-up, including list of any outcome data to be collected for participants who discontinue or deviate from intervention protocols | p18 protocol |
| Data management | 19 | Plans for data entry, coding, security, and storage, including any related processes to promote data quality (eg, double data entry; range checks for data values). Reference to where details of data management procedures can be found, if not in the protocol | p28 protocol |
| Statistical methods | 20a | Statistical methods for analysing primary and secondary outcomes. Reference to where other details of the statistical analysis plan can be found, if not in the protocol | P14 manuscript (p24, p35 protocol) |
| | 20b | Methods for any additional analyses (eg, subgroup and adjusted analyses) | P14 manuscript (p27 protocol) |
| | 20c | Definition of analysis population relating to protocol non-adherence (eg, as randomised analysis), and any statistical methods to handle missing data (eg, multiple imputation) | P14 manuscript (p28, p35 protocol) |



For peer review only - http://bmjopen.bmj.com/site/about/guidelines.xhtml

**Methods: Monitoring**

| | | | |
|---|---|---|---|
| Data monitoring | 21a | Composition of data monitoring committee (DMC); summary of its role and reporting structure; statement of whether it is independent from the sponsor and competing interests; and reference to where further details about its charter can be found, if not in the protocol. Alternatively, an explanation of why a DMC is not needed | P15 manuscript (p27, p28 protocol) |
| | 21b | Description of any interim analyses and stopping guidelines, including who will have access to these interim results and make the final decision to terminate the trial | P15 manuscript (p27 protocol) |
| Harms | 22 | Plans for collecting, assessing, reporting, and managing solicited and spontaneously reported adverse events and other unintended effects of trial interventions or trial conduct | P15 manuscript (p21-24 protocol) |
| Auditing | 23 | Frequency and procedures for auditing trial conduct, if any, and whether the process will be independent from investigators and the sponsor | n/a |

**Ethics and dissemination**

| | | | |
|---|---|---|---|
| Research ethics approval | 24 | Plans for seeking research ethics committee/institutional review board (REC/IRB) approval | P15 manuscript (P1, p29 protocol) |
| Protocol amendments | 25 | Plans for communicating important protocol modifications (eg, changes to eligibility criteria, outcomes, analyses) to relevant parties (eg, investigators, REC/IRBs, trial participants, trial registries, journals, regulators) | P15 manuscript (p2 protocol) |
| Consent or assent | 26a | Who will obtain informed consent or assent from potential trial participants or authorised surrogates, and how (see Item 32) | P16 protocol |
| | 26b | Additional consent provisions for collection and use of participant data and biological specimens in ancillary studies, if applicable | n/a |
| Confidentiality | 27 | How personal information about potential and enrolled participants will be collected, shared, and maintained in order to protect confidentiality before, during, and after the trial | p29-30 protocol |
| Declaration of interests | 28 | Financial and other competing interests for principal investigators for the overall trial and each study site | p21 manuscript |


For peer review only - http://bmjopen.bmj.com/site/about/guidelines.xhtml

| | | | |
|---|---|---|---|
| Access to data | 29 | Statement of who will have access to the final trial dataset, and disclosure of contractual agreements that limit such access for investigators | p21 manuscript (P30 protocol) |
| Ancillary and post-trial care | 30 | Provisions, if any, for ancillary and post-trial care, and for compensation to those who suffer harm from trial participation | P30 protocol |
| Dissemination policy | 31a | Plans for investigators and sponsor to communicate trial results to participants, healthcare professionals, the public, and other relevant groups (eg, via publication, reporting in results databases, or other data sharing arrangements), including any publication restrictions | P30 protocol |
| | 31b | Authorship eligibility guidelines and any intended use of professional writers | p21 manuscript (P30 protocol) |
| | 31c | Plans, if any, for granting public access to the full protocol, participant-level dataset, and statistical code | n/a |
| **Appendices** | | | |
| Informed consent materials | 32 | Model consent form and other related documentation given to participants and authorised surrogates | Included as supplementary |
| Biological specimens | 33 | Plans for collection, laboratory evaluation, and storage of biological specimens for genetic or molecular analysis in the current trial and for future use in ancillary studies, if applicable | n/a |

*It is strongly recommended that this checklist be read in conjunction with the SPIRIT 2013 Explanation & Elaboration for important clarification on the items. Amendments to the protocol should be tracked and dated. The SPIRIT checklist is copyrighted by the SPIRIT Group under the Creative Commons "Attribution-NonCommercial-NoDerivs 3.0 Unported" license.

For peer review only
